# Safety and pharmacokinetics of the Fc-modified HIV-1 human monoclonal antibody VRC01LS: A Phase 1 open-label clinical trial in healthy adults

**DOI:** 10.1371/journal.pmed.1002493

**Published:** 2018-01-24

**Authors:** Martin R. Gaudinski, Emily E. Coates, Katherine V. Houser, Grace L. Chen, Galina Yamshchikov, Jamie G. Saunders, LaSonji A. Holman, Ingelise Gordon, Sarah Plummer, Cynthia S. Hendel, Michelle Conan-Cibotti, Margarita Gomez Lorenzo, Sandra Sitar, Kevin Carlton, Carolyn Laurencot, Robert T. Bailer, Sandeep Narpala, Adrian B. McDermott, Aryan M. Namboodiri, Janardan P. Pandey, Richard M. Schwartz, Zonghui Hu, Richard A. Koup, Edmund Capparelli, Barney S. Graham, John R. Mascola, Julie E. Ledgerwood, the VRC 606 Study Team

**Affiliations:** 1 Vaccine Research Center, National Institute of Allergy and Infectious Diseases, National Institutes of Health, Bethesda, Maryland, United States of America; 2 Vaccine Clinical Research Branch, Division of AIDS, National Institute of Allergy and Infectious Diseases, National Institutes of Health, Rockville, Maryland, United States of America; 3 Department of Microbiology and Immunology, Medical University of South Carolina, Charleston, South Carolina, United States of America; 4 Biostatistics Research Branch, Division of Clinical Research, National Institute of Allergy and Infectious Diseases, National Institutes of Health, Bethesda, Maryland, United States of America; 5 School of Medicine and Skaggs School of Pharmacy and Pharmaceutical Sciences, University of California San Diego, San Diego, California, United States of America; Heinrich Pette Institute, GERMANY

## Abstract

**Background:**

VRC01 is a human broadly neutralizing monoclonal antibody (bnMAb) against the CD4-binding site of the HIV-1 envelope glycoprotein (Env) that is currently being evaluated in a Phase IIb adult HIV-1 prevention efficacy trial. VRC01LS is a modified version of VRC01, designed for extended serum half-life by increased binding affinity to the neonatal Fc receptor.

**Methods and findings:**

This Phase I dose-escalation study of VRC01LS in HIV-negative healthy adults was conducted by the Vaccine Research Center (VRC) at the National Institutes of Health (NIH) Clinical Center (Bethesda, MD). The age range of the study volunteers was 21–50 years; 51% of study volunteers were male and 49% were female. Primary objectives were safety and tolerability of VRC01LS intravenous (IV) infusions at 5, 20, and 40 mg/kg infused once, 20 mg/kg given three times at 12-week intervals, and subcutaneous (SC) delivery at 5 mg/kg delivered once, or three times at 12-week intervals. Secondary objectives were pharmacokinetics (PK), serum neutralization activity, and development of antidrug antibodies. Enrollment began on November 16, 2015, and concluded on August 23, 2017. This report describes the safety data for the first 37 volunteers who received administrations of VRC01LS. There were no serious adverse events (SAEs) or dose-limiting toxicities. Mild malaise and myalgia were the most common adverse events (AEs). There were six AEs assessed as possibly related to VRC01LS administration, and all were mild in severity and resolved during the study. PK data were modeled based on the first dose of VRC01LS in the first 25 volunteers to complete their schedule of evaluations. The mean (±SD) serum concentration 12 weeks after one IV administration of 20 mg/kg or 40 mg/kg were 180 ± 43 μg/mL (*n* = 7) and 326 ± 35 μg/mL (*n* = 5), respectively. The mean (±SD) serum concentration 12 weeks after one IV and SC administration of 5 mg/kg were 40 ± 3 μg/mL (*n* = 2) and 25 ± 5 μg/mL (*n* = 9), respectively. Over the 5–40 mg/kg IV dose range (*n* = 16), the clearance was 36 ± 8 mL/d with an elimination half-life of 71 ± 18 days. VRC01LS retained its expected neutralizing activity in serum, and anti-VRC01 antibody responses were not detected. Potential limitations of this study include the small sample size typical of Phase I trials and the need to further describe the PK properties of VRC01LS administered on multiple occasions.

**Conclusions:**

The human bnMAb VRC01LS was safe and well tolerated when delivered intravenously or subcutaneously. The half-life was more than 4-fold greater when compared to wild-type VRC01 historical data. The reduced clearance and extended half-life may make it possible to achieve therapeutic levels with less frequent and lower-dose administrations. This would potentially lower the costs of manufacturing and improve the practicality of using passively administered monoclonal antibodies (mAbs) for the prevention of HIV-1 infection.

**Trial registration:**

ClinicalTrials.gov NCT02599896

## Abstract 2

John Mascola and colleagues study the safety and pharmacokinetics of VRC01LS, a broadly neutralizing anti-HIV-1 antibody designed to have an extended half-life in serum, in healthy people.

## Author summary

**Why was this study done?:**

- New HIV-1 infections continue to occur despite improvements in traditional prevention strategies. Novel interventions to prevent acquisition of HIV-1 infection may help to turn the tide of the global pandemic.
- Human monoclonal antibodies capable of neutralizing many strains of HIV-1 have been discovered. These antibodies, referred to as broadly neutralizing antibodies (bnAbs), can be produced as biologic medications and their ability to protect people from HIV-1 infection is being investigated.
- This study evaluated a new form of an HIV-1 antibody with the potential to remain in the blood for a longer period of time than traditional antibodies. If the antibodies stay in the blood longer, then people may be protected for longer periods of time and require less frequent dosing.

**What did the researchers do and find?:**

- We modified a promising antibody called VRC01 so that it stays in the blood for a longer period of time. The modified antibody, VRC01LS, works by taking advantage of how the body recycles its own antibodies, so that the body prevents VRC01LS from being broken down.
- We gave VRC01LS to healthy adults for the first time, to see how safe it was and to understand how long it stays in the body compared to unmodified VRC01.
- In this study, VRC01LS was found to be safe. There were no serious health consequences for participants, although some people were bothered by mild muscle aches and feeling tired.
- Compared to historical data for VRC01, VRC01LS stays in the blood more than four times longer than VRC01.
- The VRC01LS in people’s blood retained the ability to neutralize the HIV-1 virus, as it has been shown to do in the laboratory.

**What do these findings mean?:**

- Our findings mean that the modifications made to VRC01 to produce VRC01LS may be useful when incorporated into other, similar antibodies that are being developed in the hope of producing medications to prevent HIV-1 infection.
- Our findings also mean that fewer doses of these HIV-1 antibodies may be required for protection, thus reducing product cost.

## Introduction

Advances in our understanding of the humoral immune responses against HIV-1 have led to the appreciation that broadly reactive neutralizing antibodies arise during the course of infection in some individuals [1–4]. Detailed studies of such donors, including the application of technologies to recover immunoglobulin genes from antigen-specific sorted or cultured B cells, have led to the isolation and characterization of numerous broadly neutralizing monoclonal antibodies (bnMAbs) targeting HIV-1 envelope glycoprotein (Env). These bnMAbs target distinct antigenic sites on the native HIV-1 Env trimer shared by multiple viral clades, including the CD4-binding site, the variable region 1 and 2 (V1V2) apex, the glycan-V3 region, the membrane-proximal external region (MPER), and the interface region between glycoprotein (gp)120 and gp41 [1–5]. Several human bnMAbs have been developed and tested in Phase I studies, including VRC01 and 3BNC117 to the CD4-binding site and 10–1074 to the glycan-V3 region [6–9]. The potency and breadth of the current generation of bnMAbs have led to interest in their clinical use both for prophylaxis and treatment of HIV-1 infection [10–12].

VRC01 is a member of a class of bnMAbs that target the CD4-binding site of HIV-1 Env, thereby disrupting viral entry into host cells [6,13–16]. VRC01 is capable of neutralizing 85%–90% of circulating HIV-1 isolates [17,18] and has a well-defined safety and tolerability profile, with known pharmacokinetic (PK) parameters in both HIV-infected [7] and uninfected [6] human populations. VRC01 is also currently being evaluated in an international Phase IIb adult HIV-1 prevention efficacy trial (clinicaltrials.gov NCT02568215, NCT 02716675). Notably, the trial is being conducted with VRC01 antibody infused intravenously, every 8 weeks.

Mutations to the constant (Fc) region of a monoclonal antibody (mAb) can be made to alter circulating half-life by taking advantage of the natural immunoglobulin G (IgG) homeostatic activities of the neonatal Fc receptor (FcRn). When IgG in the serum is endocytosed from the circulation by endothelial cells, the low pH endosomal environment promotes binding between the IgG Fc region and the FcRn. At physiological pH, bound IgG is re-released into the serum through an endosomal recycling mechanism, whereas unbound IgG is degraded within the cell [19,20]. The serum half-life is therefore closely related to the degree to which IgG binds to the FcRn [14]. In designing a mAb for which a longer half-life is desirable, an increased binding affinity to FcRn can be achieved through engineering amino acid substitutions into the FcRn binding site on the antibody. Changes to specific amino acids of the antibody Fc region are known to increase FcRn binding affinity, including Thr250, Met252, Ser254, Thr256, Thr307, Glu380, Met428, His433, and Asn434 [21–27]. Making multiple changes may have a synergistic effect. Motavizumab-YTE, an antibody to glycoprotein F of respiratory syncytial virus (RSV), is an example of an investigational mAb that underwent Fc modification in order to optimize FcRn binding, which resulted in an extended serum half-life [26,28]. This YTE change (M252Y/S254T/T256E) was engineered into another mAb against RSV, termed MEDI8897, and its half-life was extended in a similar manner as seen with motazvizumab [29].

VRC01LS is a human immunoglobulin G subclass 1 (IgG1) HIV-1 bnMAb that is identical to VRC01, with the exception of two amino acid changes (M428L and N434S) in the Fc region intended to extend serum half-life. The LS mutations result in enhanced IgG-FcRn binding but do not affect binding to the Fc-gamma receptor and thus do not impair Fc-mediated effector functions, such as antibody dependent cellular cytotoxicity (ADCC) [30]. A previously reported LS change to the mAb bevacizumab, targeting the vascular endothelial growth factor A, resulted in an 11-fold increase in FcRn binding affinity above the wild-type antibody [31]. We previously demonstrated that in nonhuman primates (NHPs), the LS mutation to VRC01 extended half-life in both serum and mucosal tissue compared to parental VRC01: its serum half-life was 2.5-fold greater and it persisted for up to 70 days in rectal tissues, whereas VRC01 was no longer detectable after 28 days [30]. Based on the results of these preclinical studies, the VRC01LS drug product was developed by the Vaccine Research Center (VRC), National Institute of Allergy and Infectious Diseases (NIAID) at the National Institutes of Health (NIH) for evaluation in humans. The VRC 606 Phase I clinical trial represents the first evaluation of the safety, tolerability, PK, and neutralizing potential of VRC01LS administration in healthy adults.

## Methods

### Study design and procedures

VRC 606 (clinicaltrials.gov NCT02599896) was a single-site Phase I open-label dose-escalation study examining the safety and PK parameters of VRC01LS in healthy, HIV-uninfected adults, aged 18–50 years. The study was conducted at the NIH Clinical Center by the VRC Clinical Trials Program, NIAID, NIH, Bethesda, Maryland. The Investigational New Drug application was sponsored by NIAID Division of AIDS. The protocol was reviewed and approved by the NIAID Institutional Review Board, and US Department of Health and Human Services guidelines for conducting clinical research were followed. All volunteers gave written informed consent prior to participation.

**Fig 1:**
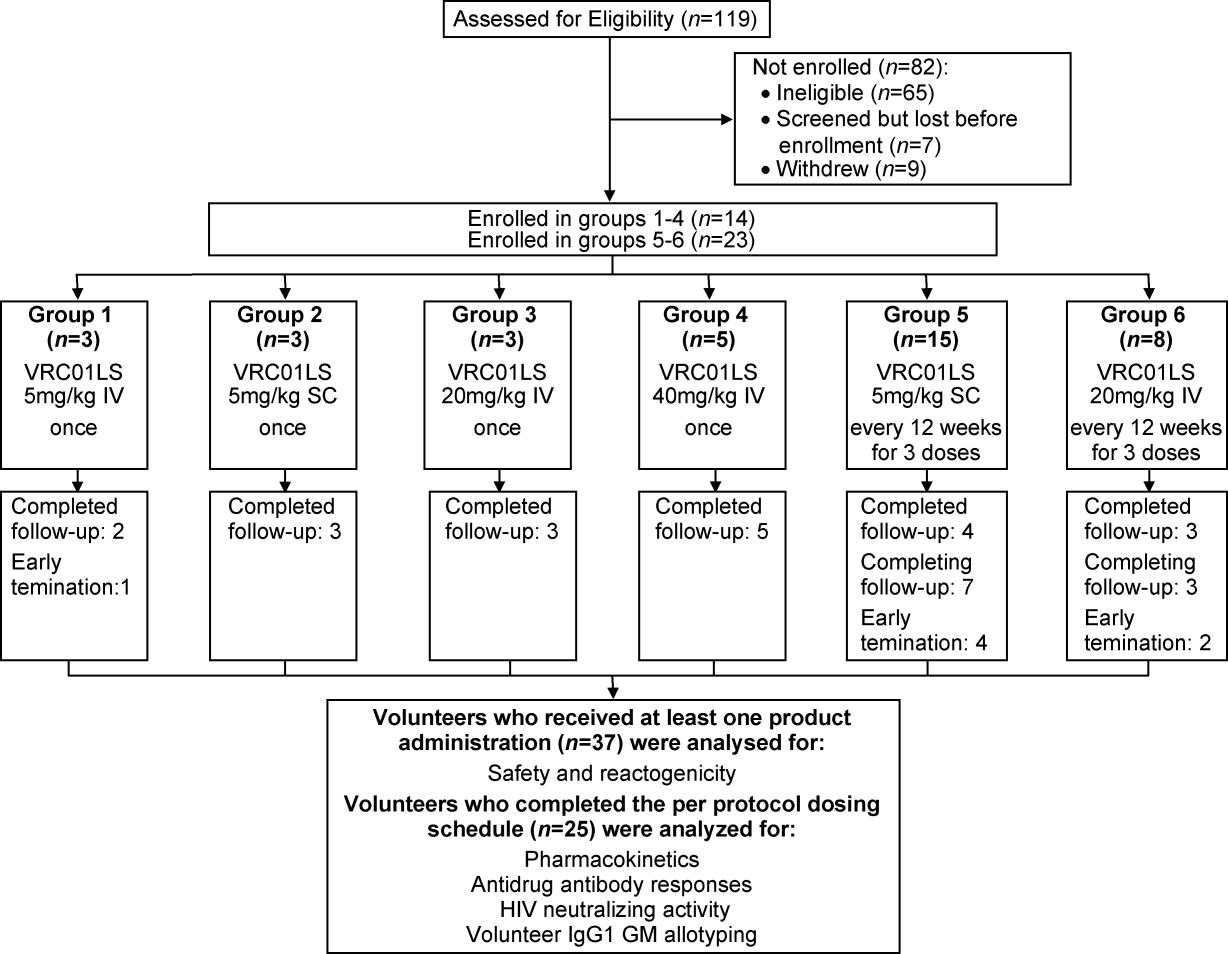
CONSORT flow diagram of the VRC 606 trial. Thirty-seven volunteers were allocated to 6 groups. All volunteers who received at least one VRC01LS administration were analyzed for safety and reactogenicity. All volunteers who completed the per-protocol dosing schedule by the time of manuscript submission (*n* = 25) were additionally analyzed for pharmacokinetic parameters, serum neutralization activity, anti-drug antibodies, and IgG1 GM allotype. These 25 volunteers were all the volunteers in groups 1–4, the first six volunteers in group 5, and the first five volunteers in group 6. GM, genetic marker; IgG1, immunoglobulin G subclass 1; IV, intravenous; SC, subcutaneous; VRC, Vaccine Research Center.

The dosages in the trial were determined based on previous trials with VRC01 [6,7]. Three groups received a single dose of intravenous (IV) VRC01LS (group 1: 5 mg/kg, group 3: 20 mg/kg, and group 4: 40 mg/kg). One group received a single dose of subcutaneous (SC) VRC01LS (group 2: 5 mg/kg). Two repeat dosing groups (group 5: 5 mg/kg SC and group 6: 20 mg/kg IV) received three product administrations 12 weeks apart (Fig 1). Groups 1, 2, and 5 were enrolled simultaneously. Groups 1 and 2 were block randomized in a 1:1 ratio using the electronic AdvantageEDC system (EMMES Corp, Rockville, MD). Sample size was consistent with a small Phase I trial design and calculated to capture adverse events (AEs).

Interim safety reviews were conducted by the Protocol Safety Review Team at protocol-specified intervals for the two dose escalations. All product administrations were monitored by a study clinician. Safety laboratory tests were obtained prior to product administration, throughout the study, and at least 4 weeks post final product administration. Volunteers kept a diary card of solicited systemic symptoms for 3 days after each dose and clinicians objectively assessed the local site on the day of product administration, the day after product administration, and one week after product administration. All AEs were recorded through 56 days following product administration, while serious adverse events (SAEs) and new chronic medical conditions were recorded for the duration of the study. AEs were coded using the Medical Dictionary for Regulatory Activities (MedDRA) and severity was graded using Version 2.0 of the “DAIDS Table for Grading the Severity of Adult and Pediatric Adverse Events” (November 2014). Volunteers were followed for 24 weeks following their final study product administration.

### Study product

VRC01 is a human IgG1 of allotype GM3. VRC01LS was modified from VRC01. The LS designation specifies methionine to leucine and asparagine to serine (M428L/N434S) changes within the C-terminus of the heavy chain constant region far outside of the antigen-binding site [31]. VRC01LS was produced in a Chinese hamster ovary (CHO) mammalian cell line under current Good Manufacturing Practice (cGMP) by VRC/NIAID/NIH at the VRC Pilot Plant operated under contract by the Vaccine Clinical Materials Program (VCMP), Leidos Biomedical Research, Inc., Frederick, MD. VRC01LS vials were filled at a concentration of 100 mg/mL. The formulation buffer was composed of 25 mM sodium citrate, 50 mM sodium chloride, and 150 mM L-arginine hydrochloride at pH 5.8. Vials were single use only and did not contain preservative.

A pharmacist prepared individual IV doses for volunteers by adding the calculated volume of VRC01LS needed to achieve the assigned mg/kg dose to a 103-mL bag of 0.9% sodium chloride. IV infusions were administered over 15–30 minutes using a volumetric pump. SC doses were administered by direct needle and syringe injection in the abdomen at a volume up to 2.5 mL per injection site.

### Volunteer IgG1 GM allotyping

Volunteers were assessed for their genetic marker (GM) 3/17 allotypes to determine if allotype-specific effects influenced the PK of VRC01LS. IgG1 markers GM 3/f and 17/z (arginine to lysine) were determined as previously described [32] by a predesigned TaqMan genotyping assay from Applied Biosystems, Inc., employing the following primers and probes: forward primer: 5′ CCCAGACCTACATCTGCAACGTGA-3′, reverse primer: 5′ CTGCCCTGGACTGGGACTGCAT-3′, reporter 1 (GM 17-specific): VIC-CTCTCACCAACTTTCTTGT-NFQ, and reporter 2 (GM 3-specific): FAM-CTCTCACCAACTCTCTTGT-NFQ.

### PK analysis

Quantification of VRC01LS concentrations was performed as previously described [6]. Briefly, VRC01LS serum concentration quantification was performed in 96 well plates on a Beckman Biomek based automation platform, utilizing the anti-idiotype mAb 5C9. Immulon-4HXB microtiter plates were coated with 5C9 overnight before blocking. Threefold dilutions were tested in duplicate wells covering the range of 1:100–1:24,300. Horseradish peroxidase-labeled goat antihuman antibody and 3,3′5,5′–tetramethylbenzidine (TMB substrate) were added for detection, and color development was halted through the addition of sulphuric acid. Plates were read at 450 nm within 30 minutes using a Molecular Devices Paradigm plate reader. Sample concentrations were quantitated using a four-parameter logistic curve regression of a standard curve of VRC01LS covering a range of 0.98–1,000 ng/ml.

PK analysis was performed using both noncompartmental and compartmental methods. Extensive PK samples were collected following IV and SC administration over 12 weeks after the first dose. PK results following the first dose in volunteers in multiple-dose groups 5 and 6 were combined with those from the single-dose groups. PK results for groups 5 and 6 following subsequent doses are still being collected and therefore are not included in this report. For the noncompartmental analysis, the area under the curve (AUC) for the VRC01LS concentration versus time curve was calculated over the first dose interval (weeks 0–12) using the trapezoidal method. The maximal concentration (C_max_) and time to maximal concentration (T_max_) were taken from the observed data. The PK data were also fit to a two-compartment model using the computer program NONMEM 7.3 (ICON, Dublin) to estimate clearance and the elimination half-life (t_1/2β_). Due to the small number of volunteers, population PK parameters were assumed to be proportional to body weight and no formal covariate assessment of population PK parameters was performed. Individual volunteer compartmental PK parameters were generated through a post hoc empiric Bayesian approach using the population VRC01LS parameter estimates and their variances.

### Neutralizing antibody assay

Viral neutralization by trial participant serum samples were measured using single-round HIV-1 Env pseudovirus infection of TZM-bl target cells. Env-pseudotyped viruses were generated by transfection of 293T/17 cells with optimized ratios of envelope-expressing plasmid and backbone vector (pSG3DEnv). A panel of three viruses was tested for neutralizing activity spanning a range of 80% inhibitory concentrations (IC_80_) for bnMAb VRC01. Negative and positive controls were included in all assays. The tested viruses were Q23.17 (subtype A), PVO.04 (subtype B), and MW965.26 (subtype C). Serum neutralization assays were performed in 384-well plates using a Beckman Biomek liquid handling system, as previously described [33]. Experimental results were displayed as the serum dilution that produced 80% neutralization (ID_80_) against the viruses tested. Predicted ID_80_ values were calculated based on the concentration of VRC01LS present in the sera and the established inhibitory concentration (IC_80_) of the antibody against each virus.

### Antidrug antibody analysis

A Meso Scale Discovery (MSD) electrochemiluminescence (ECL) homogenous bridging assay was developed to identify antidrug antibody (ADA) elicited by VRC01LS infusion, as previously described [6]. Briefly, diluted volunteer serum samples were incubated with equimolar amounts of SULFO-TAG–and biotin-labeled VRC01LS overnight, which bound any ADA present in the sera. Complexes resulting from ADA present in the volunteer serum were captured on a precoated streptavidin MSD plate and detected through the SUFO-TAG–labeled VRC01LS using an MSD sector imager 2400. An ECL threshold was established as previously described [34]. Values above the ECL unit threshold underwent an additional assay using the antigen-binding fragment (Fab) of VRC01 as the antigen in a sandwich ELISA format in order to determine whether the ECL result beyond threshold was due to background or genuine ADA against the mAb. Briefly, the VRC01 Fab was coated onto an MSD plate and serial dilutions of volunteer or control sera were analyzed. Data from this assay are represented as AUC for the dilution series and compared to healthy negative controls or ADA positive NHP sera.

## Results

### Study population

A total of 37 volunteers were enrolled in the study between November 16, 2015, and August 23, 2017 (Fig 1 and Table 1): 19 males (51%) and 18 females (49%). Mean volunteer weight was 74 kg, ranging from 46.5 to 96.5 kg. At the time of publication, 30 volunteers had completed their scheduled infusions and 20 had completed the protocol. One volunteer in group 1 completed study product administration but withdrew prior to study completion, citing issues with time commitment. Three volunteers withdrew from group 5. One group 5 volunteer who withdrew received two of three scheduled administrations and was discontinued from further product administration until a medical evaluation for a concurrent illness could be completed. Two group 5 volunteers withdrew after one scheduled administration: one cited injection site pain while the other had hyperpigmentation at the injection site, which later resolved. Two volunteers withdrew from group 6: one volunteer withdrew following one of three product infusions, citing problems with time commitment, and one volunteer was lost to follow-up after completing all three infusions prescribed by the protocol.

**Table 1:** Demographic characteristics of study participants.

*Category*	*Subcategory*	*Group 1 (N = 3)*	*Group 2 (N = 3)*	*Group 3 (N = 3)*	*Group 4 (N = 5)*	*Group 5 (N = 15)*	*Group 6 (N = 8)*	*Overall (N = 37)*
*n (%)*								
Sex	Male	2 (67)	2 (67)	2 (67)	4 (80)	4 (27)	5 (63)	19 (51)
	Female	1 (33)	1 (33)	1 (33)	1 (20)	11 (73)	3 (38)	18 (49)
Age (years)*	21–30	2 (67)	1 (33)	3 (100)	2 (40)	7 (47)	6 (75)	21 (57)
	31–40	1 (33)	2 (67)	0 (0)	3 (60)	4 (27)	1 (13)	11 (30)
	41–50	0 (0)	0 (0)	0 (0)	0 (0)	4 (27)	1 (13)	5 (14)
Race	American Indian/Alaskan Native	0 (0)	0 (0)	0 (0)	0 (0)	1 (7)	0 (0)	1 (3)
	Asian	0 (0)	1 (33)	0 (0)	0 (0)	0 (0)	1 (13)	2 (5)
	Black or African American	0 (0)	1 (33)	0 (0)	2 (40)	2 (13)	1 (13)	6 (16)
	White	3 (100)	1 (33)	1 (33)	3 (60)	11 (73)	5 (63)	24 (65)
	Multiracial	0 (0)	0 (0)	2 (67)	0 (0)	1 (7)	1 (13)	4 (11)
Ethnicity	Non-Hispanic/Latino	2 (67)	3 (100)	3 (100)	5 (100)	14 (93)	7 (88)	34 (92)
	Hispanic/Latino	1 (33)	0 (0)	0 (0)	0 (0)	0 (0)	1 (13)	2 (5)
	Unknown/Not Reported	0 (0)	0 (0)	0 (0)	0 (0)	1 (7)	0 (0)	1 (3)
Mean Weight	kg (s.d.)	72.7 (3.2)	71.5 (1.5)	63.0 (8.9)	71.5 (9.2)	76.9 (15)	74.6 (19)	73.8 (13)
Education^†^	Secondary	0 (0)	0 (0)	0 (0)	1 (20)	0 (0)	1 (13)	2 (5)
	College/University	2 (67)	2 (67)	2 (67)	4 (80)	8 (53)	5 (63)	23 (62)
	Advanced Degree	1 (33)	1 (33)	1 (33)	0 (0)	7 (47)	2 (25)	12 (32)

*There were no participants aged 18–20.

^†^There were no participants with only a primary education level.

s.d., standard deviation.

### Safety

To date, a total of 70 VRC01LS administrations have occurred during the trial. VRC01LS was safe and well tolerated, and there were no SAEs. Post administration symptoms were mild or moderate for both objective local and systemic reactogenicity, assessed at their prescribed time points (Tables 2 and 3).

**Table 2:** Maximum local reactogenicity up to day 7 after VRC01LS administration*.

*Symptom Intensity*	*IV Administration*				*SC Administration*	
	*Group 1 (5 mg/kg IV)*	*Group 3 (20 mg/kg IV)*	*Group 4 (40 mg/kg IV)*	*Group 6 (20 mg/kg IV by repeat dosing)*	*Group 2 (5 mg/kg SC)*	*Group 5 (5 mg/kg SC by repeat dosing)*
	*(N = 3)*	*(N = 3)*	*(N = 5)*	*(N = 8)*	*(N = 3)*	*(N = 15)*
*n (%)*						
Pain/Tenderness						
None	3(100.0)	2(66.7)	5(100.0)	7(87.5)	1(33.3)	4(26.7)
Mild	0(0.0)	1(33.3)	0(0.0)	1(12.5)	2(66.7)	10(66.7)
Moderate	0(0.0)	0(0.0)	0(0.0)	0(0.0)	0(0.0)	1(6.7)
Bruising						
None	3(100.0)	3(100.0)	5(100.0)	7(87.5)	3(100.0)	14(93.3)
Mild	0(0.0)	0(0.0)	0(0.0)	1(12.5)	0(0.0)	1(6.7)
Swelling						
None	3(100.0)	3(100.0)	5(100.0)	8(100.0)	3(100.0)	13(86.7)
Mild	0(0.0)	0(0.0)	0(0.0)	0(0.0)	0(0.0)	2(13.3)
Redness						
None	3(100.0)	3(100.0)	5(100.0)	8(100.0)	2(66.7)	14(93.3)
Mild	0(0.0)	0(0.0)	0(0.0)	0(0.0)	1(33.3)	1(6.7)
Pruritus						
None	3(100.0)	3(100.0)	5(100.0)	8(100.0)	3(100.0)	15(100.0)
Any Local Symptom						
None	3(100.0)	2(66.7)	5(100.0)	6(75.0)	1(33.3)	3(20.0)
Mild	0(0.0)	1(33.3)	0(0.0)	2(25.0)	2(66.7)	11(73.3)
Moderate	0(0.0)	0(0.0)	0(0.0)	0(0.0)	0(0.0)	1(6.7)

*There were no severe reactions throughout the trial.

IV, intravenous; SC, subcutaneous.

**Table 3:** Self-reported systemic reactogenicity up to 3 days after VRC01LS administration*.

*Symptom Intensity*	*IV Administration*				*SC Administration*	
	*Group 1 (5 mg/kg IV)*	*Group 3 (20 mg/kg IV)*	*Group 4 (40 mg/kg IV)*	*Group 6 (20 mg/kg IV by repeat dosing)*	*Group 2 (5 mg/kg SC)*	*Group 5 (5 mg/kg SC by repeat dosing)*
	*(N = 3)*	*(N = 3)*	*(N = 5)*	*(N = 8)*	*(N = 3)*	*(N = 15)*
*n (%)*						
Malaise						
None	1(33.3)	2(66.7)	4(80.0)	8(100.0)	3(100.0)	9(60.0)
Mild	2(66.7)	1(33.3)	1(20.0)	0(0.0)	0(0.0)	6(40.0)
Myalgia						
None	1(33.3)	3(100.0)	5(100.0)	7(87.5)	2(66.7)	13(86.7)
Mild	2(66.7)	0(0.0)	0(0.0)	1(12.5)	1(33.3)	2(13.3)
Headache						
None	3(100.0)	2(66.7)	5(100.0)	8(100.0)	2(66.7)	13(86.7)
Mild	0(0.0)	1(33.3)	0(0.0)	0(0.0)	1(33.3)	2(13.3)
Chills						
None	3(100.0)	3(100.0)	5(100.0)	8(100.0)	3(100.0)	15(100.0)
Nausea						
None	3(100.0)	3(100.0)	5(100.0)	8(100.0)	2(66.7)	12(80.0)
Mild	0(0.0)	0(0.0)	0(0.0)	0(0.0)	1(33.3)	3(20.0)
Temperature						
None	3(100.0)	3(100.0)	5(100.0)	8(100.0)	3(100.0)	15(100.0)
Joint Pain						
None	3(100.0)	3(100.0)	5(100.0)	8(100.0)	3(100.0)	15(100.0)
Any Systemic Symptom						
None	1(33.3)	2(66.7)	4(80.0)	7(87.5)	1(33.3)	9(60.0)
Mild	2(66.7)	1(33.3)	1(20.0)	1(12.5)	2(66.7)	6(40.0)

*There were no moderate or severe reactions throughout the trial.

IV, intravenous; SC, subcutaneous.

Six AEs were assessed as possibly related to VRC01LS administration and all were mild in severity. Two reports of diarrhea, one in group 1 and one in group 5, occurred on the day of VRC01LS administration and resolved the same day. One group 2 volunteer experienced lightheadedness on the day following administration, and symptoms resolved within 24 hours. One AE in group 5 pertained to an injection site reaction with hyperpigmentation that resolved 14 days post administration. Two AEs in group 5 were due to elevated alanine aminotransferase levels (56 and 69 IU/L) on day 14 post injection and both resolved within 15 days after onset. No volunteer had a positive HIV enzyme immunoassay (EIA) response during the study.

### VRC01LS PK

**Fig 2:**
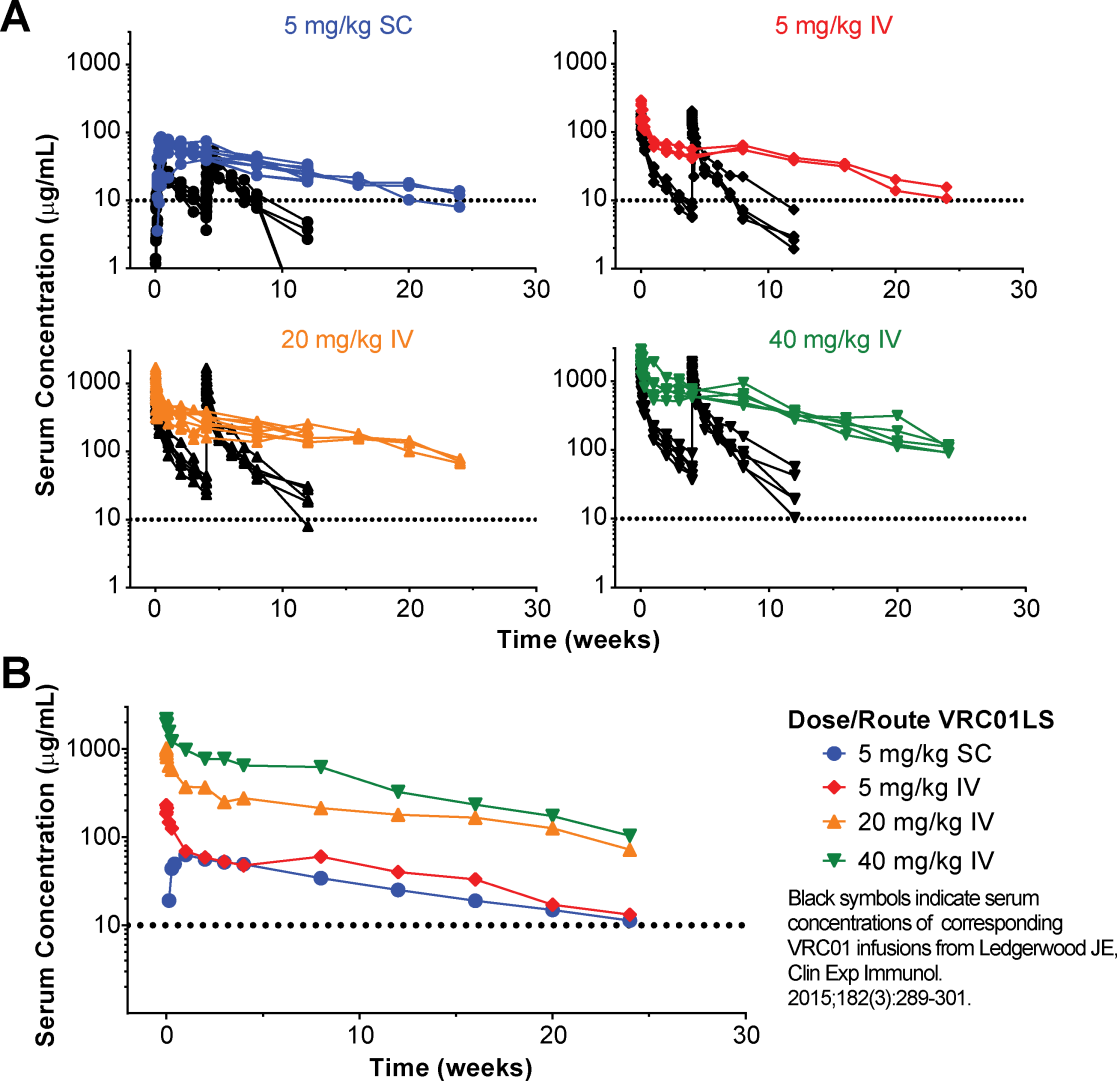
Measurement of antibody serum concentration (μg/mL). (A) Serum VRC01LS concentrations (colored plots) are shown from first measurement through week 24 after a single administration. The infusion dose and route are as specified in the legend. All values are the mean of duplicate samples run in different wells within the same plate. Previously published VRC01 concentrations based on historical data (black plots) after administration at weeks 0 and 4 are shown for comparison. (B) Geometric mean serum VRC01LS concentrations per group over time. The dotted line at 10 μg/mL on each graph is shown as a reference value. IV, intravenous; SC, subcutaneous.

**Fig 3:**
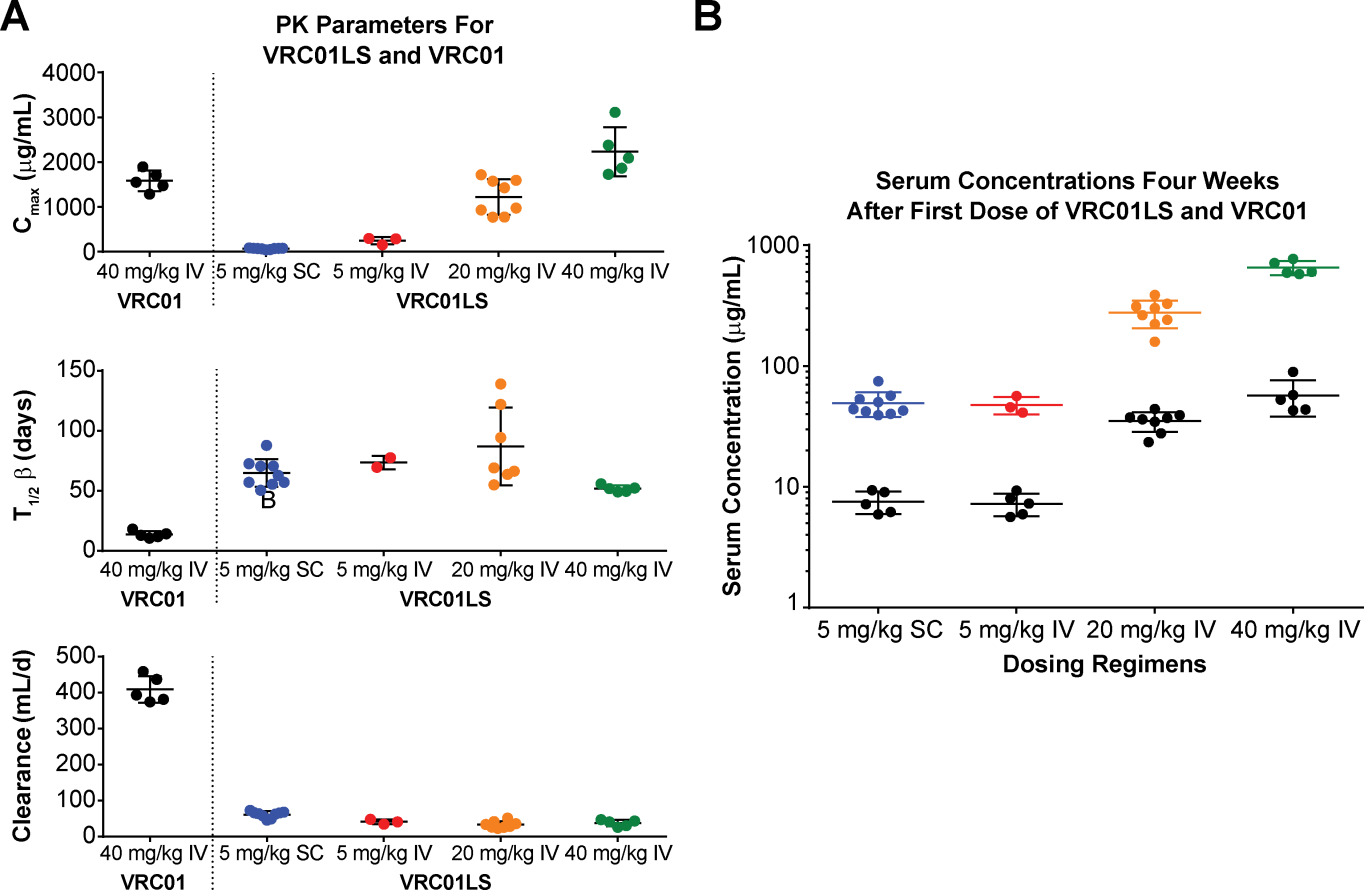
Post infusion serum antibody concentrations and PK parameters by dose and route. (A) C_max_ (top panel) showing expected dose-dependent increases of VRC01LS, t_1/2β_ (middle panel), and antibody clearance (bottom panel), showing similar values for doses and routes. Bars show mean values, with error bars indicating s.d. Previously published VRC01 data following 40 mg/kg infusion are shown for comparison. (B) VRC01LS and VRC01 serum concentrations 4 weeks after a single administration, as indicated on the x-axis. Each value is the mean of duplicate wells in the same plate. VRC01 data (black dots) were derived from historical data. C_max_, maximal concentration; IV, intravenous; PK, pharmacokinetic; SC, subcutaneous; s.d., standard deviation; t_1/2β_, elimination half-life.

Antibody serum concentrations for volunteers who received at least one administration of VRC01LS were analyzed to determine VRC01LS PK properties (Fig 2 and Table 4). A total of 25 volunteers had PK analysis, including the 16 volunteers who received at least one IV infusion and 9 volunteers who received at least one SC administration. Dose-dependent increases in C_max_ and trough levels were observed. The mean (± SD) C_max_ after one IV infusion were 246 ± 78, 1221 ± 397, and 2234 ± 548 μg/mL for 5 mg/kg, 20 mg/kg, and 40 mg/kg, respectively. Mean (± SD) 12-week serum trough concentrations were 40 ± 2.7, 180 ± 43, and 326 ± 35 μg/mL for these groups (Fig 3A and Table 4). Taking all IV administrations together, the overall VRC01LS clearance was 36 ± 8 mL/d with a t_1/2β_ of 71 ± 18 days. The mean (± SD) PK parameters following one 5 mg/kg SC dose were C_max_ of 69 ± 15 μg/mL, 12-week serum trough concentration of 25 ± 5 μg/mL, and t_1/2β_ of 66 ± 24 days (Fig 3 and Table 4). Historical data showed that the t_1/2_ for the wild-type VRC01 is 15 days [6]; thus, VRC01LS displays a t_1/2β_ more than 4-fold longer than VRC01. Serum VRC01 concentrations based on historical data are plotted alongside VRC01LS values in Fig 2 for comparison. Notably, serum concentrations 12 weeks after a single infusion of VRC01LS were higher than after two infusions (weeks 0, 4) of VRC01 (Fig 2). After IV or SC infusion of 5 mg/kg of VRC01LS, serum concentrations above 10 μg/mL were maintained for more than 20 weeks (Fig 2A and 2B). We were able to compare serum concentrations at 4 weeks post a single infusion for VRC01 and VRC01LS. Regardless of infusion dose or route of administration, VRC01LS serum concentrations were approximately 5-fold higher than corresponding levels of VRC01 (Fig 3B).

**Table 4:** VRC01LS mean PK parameter values.

Group and dose	C_max_	T_max_	CL	t_1/2β_	AUC	4-week post infusion concentration (μg/mL)	12-week post infusion concentration (μg/mL)
			Mean (s.d.)				
**IV dosing**							
5 mg/kg (*n* = 3)	246 (78)	0.07 (0.08)	40 (7)	83 (11)	4896 (499)*	48 (8)	40 (2.7)*
20 mg/kg (*n* = 8)	1,221 (397)	0.2 (0.3)	33 (8)	76 (19)	23,368 (5279)*	276 (71)	180 (43)*
40 mg/kg (*n* = 5)	2,234 (548)	0.05 (0.02)	38 (9)	55(7)	57,099 (13679)	651 (86)	326 (35)
Overall IV (*n* = 16)			36 (8)	71 (18)			
**SC dosing**							
5 mg/kg (*n* = 9)	69 (15)	9.0 (7.9)	61 (5)^+^	66 (24)	3,777 (814)	49 (11)	25 (5.3)

Includes PK parameters from volunteers who received one or more doses of VRC01LS.

* AUC and week 12 values are missing one volunteer from the overall group due to missing week 12 PK samples.

^+^ Value following SC administration represents CL/F.

AUC, area under the curve, 0–12 weeks (μg × day/mL); CL, clearance (mL/day); CL/F, apparent clearance; C_max_, maximal serum concentration (μg/mL); IV, intravenous; PK, pharmacokinetic; SC, subcutaneous; s.d., standard deviation; T_max_, time to maximal concentration (days); t_1/2β_, elimination half-life (days).

### VRC01LS neutralizing activity is retained in serum after antibody infusion

**Fig 4:**
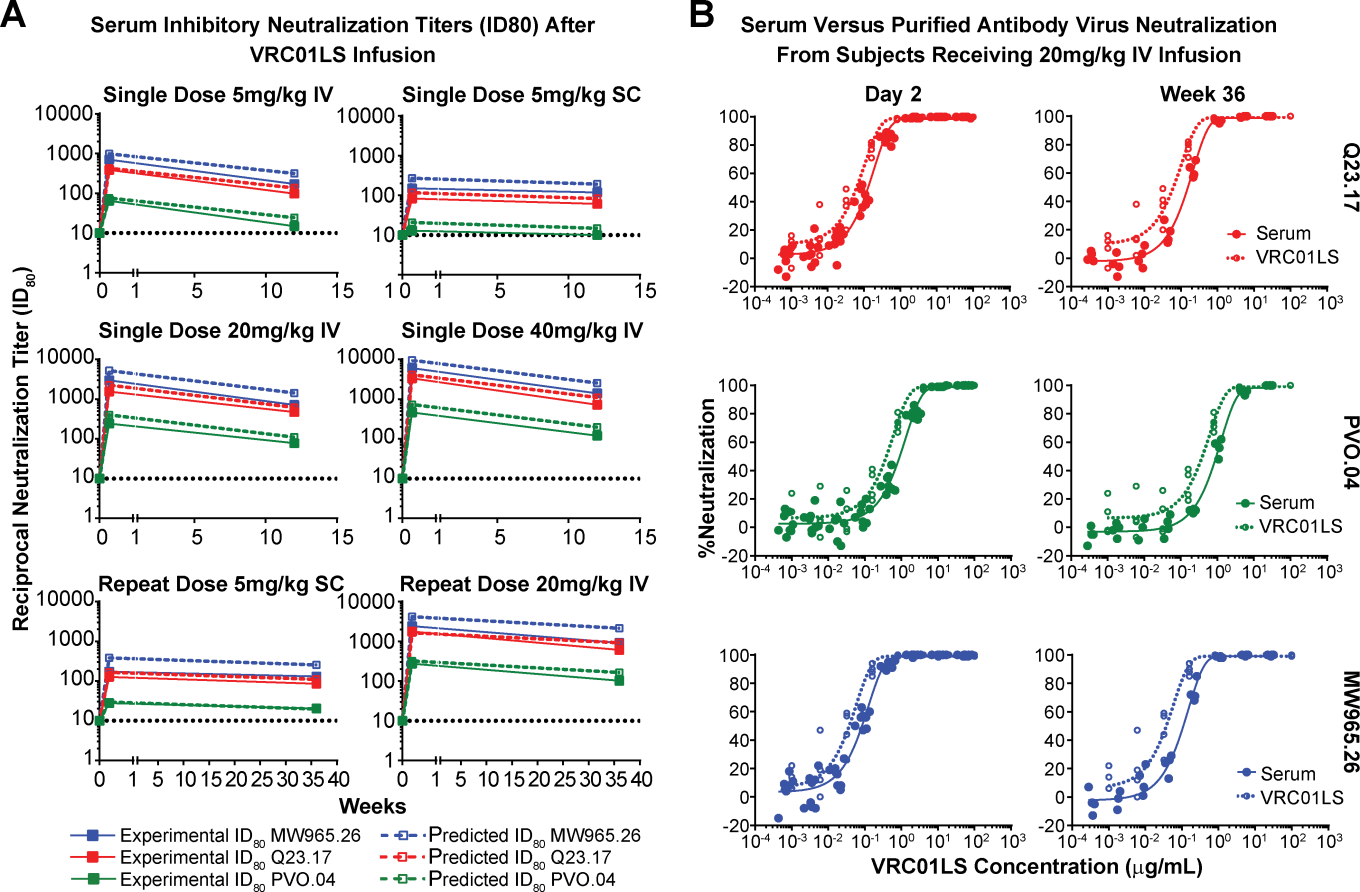
Neutralizing ability of VRC01LS is maintained in serum after infusion. (A) Serum inhibitory neutralization titers (ID_80_) after VRC01LS infusion. Solid lines and closed squares indicate the reciprocal serum dilution that produces 80% virus neutralization for three viral strains. Dashed lines and open squares indicate predicted ID_80_ based on the measured amount of VRC01LS in the serum and the known inhibitory concentration (IC_80_) of VRC01LS for the viruses tested: MW965.26, 0.128 μg/mL; Q23.17, 0.298 μg/mL; and PVO.04, 1.66 μg/mL. All final time points were assessed 12 weeks after the final dose was administered. Values are the mean of duplicate wells run in the same plate. (B) Examples of serum and VRC01LS neutralization curves. Serum from day 2 and week 36 samples (closed circles) were used to generate neutralization curves (solid lines) that are compared to neutralization curves generated with uninfused VRC01LS IgG antibody (open circles and dashed lines) at different dilutions. The x-axis displays both the VRC01LS concentration acquired from serum and in vitro dilutions of uninfused antibody. IC_80_, 80% inhibitory concentration; ID_80_, dilution that produced 80% neutralization; IgG, immunoglobulin G; IV, intravenous; SC, subcutaneous.

Serum neutralization was assessed using a standardized neutralization assay and three Env-pseudoviral strains that differed in their sensitivity to neutralization by VRC01LS [17]. Over the course of the trial, serum inhibitory dilution neutralization titers (ID_80_) were assessed at 0, 2, and 84 days after a single VRC01LS infusion in groups 1–4 and at 0, 2, and 252 days after repeat doses in groups 5 and 6 (Fig 4). In all cases, the measured serum neutralization tracked closely with the predicted serum neutralization based on the measured concentration of VRC01LS (Fig 4A). As expected, serum neutralization activity increased with escalating VRC01LS doses. Thus, VRC01LS retains its expected neutralizing activity in serum, even after 36 weeks in serum. Confirmatory experiments compared serum with a known (measured) concentration of VRC01LS to the VRC01LS IgG, and the neutralization curves were similar (Fig 4B).

### Lack of elicitation of anti-VRC01 antibodies in serum

**Fig 5:**
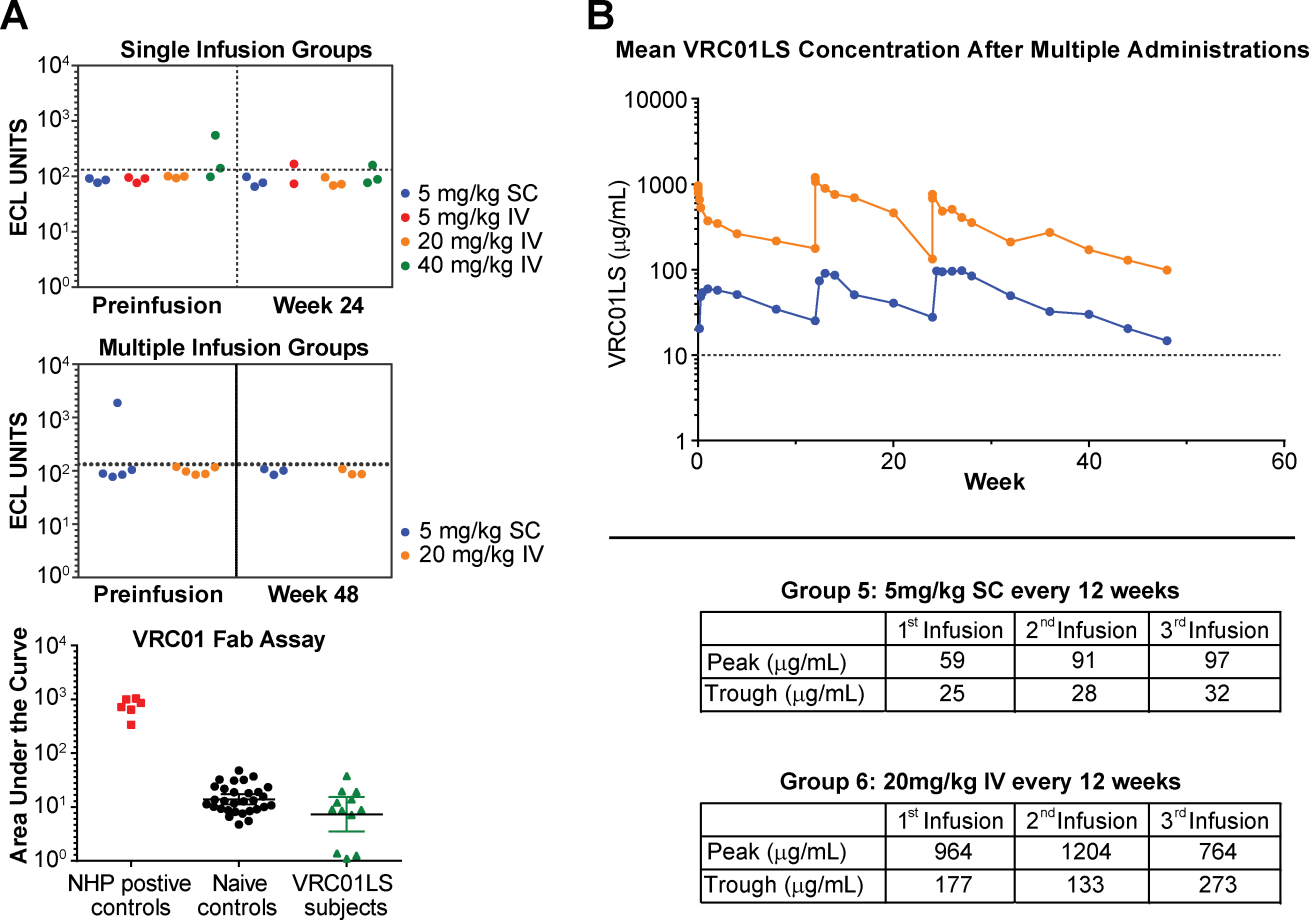
Evaluation of multiple administration of VRC01LS and assessment for anti-VRC01 antibodies. (A) ECL assay to assess for serum antibodies generated against VRC01. Data are shown for single (top panel) and multiple (middle panel) infusion groups. The few samples above the ECL cutoff value (dotted line) underwent additional analysis using VRC01 Fab as the antigen. These samples were compared to positive controls obtained from NHPs known to produce ADA against human antibodies, and negative controls obtained from humans who have never received VRC01 or VRC01LS (bottom panel). All samples were negative for ADA based on these assays. (B) Mean serum VRC01LS concentration following three administrations of 5 mg/kg SC (*n* = 6 volunteers, blue line) or 20 mg/kg IV (*n* = 5 volunteers, orange line). The table insert shows the values for peak concentration assessed within one day after administration and trough concentrations assessed 12 weeks after each administration. The dotted line at 10 μg/mL is shown as a reference value. ADA, antidrug antibody; ECL, electrochemiluminescence; Fab, antigen-binding fragment; IV, intravenous; NHP, nonhuman primate; SC, subcutaneous.

Serum samples were tested for reactivity against VRC01LS and the isolated Fab region of VRC01. Using both assays, anti-VRC01LS antibody responses, also called ADA, were not detected in any volunteer at any time point during the trial (Fig 5). In addition, volunteers who received three infusions of VRC01LS showed no evidence of diminished peak or trough responses (Fig 5B).

### Host volunteer IgG allotype

Study participants were assessed for their IgG1 allotype. Allotypes are designated GM3 or GM17, where GM refers to “genetic marker”, and volunteers can be homozygous or heterozygous. The majority of volunteers, 56%, were heterozygous GM 3/17, with 32% GM 3/3 homozygous and 12% GM 17/17 homozygous. There were no obvious correlations found between the GM allotype of volunteers and measured PK parameters.

## Discussion

An expanded armamentarium of prevention and treatment methods is required to more adequately control the HIV-1 pandemic [35]. Mounting laboratory, preclinical, and clinical experience with HIV-1 bnMAbs has provided a greater understanding of their potency, breadth of reactivity, and potential to prevent or treat HIV-1 infection [6,9,36,37]. This experience is being acquired in tandem with two related international placebo-controlled Phase IIb efficacy trials assessing the impact of passive infusion of VRC01 on the risk of acquiring HIV-1 infection (clinicaltrials.gov NCT02716675, NCT02256631). In this Phase I study, we evaluated a variant of VRC01, designed to have an extended serum half-life, for safety and PK parameters. We observed that VRC01LS was safe and well tolerated and displayed a serum half-life more than four times longer than wild-type VRC01 as indicated by historical data [11,12]. The VRC01LS antibody retained its neutralizing activity in serum for the 48-week duration of this study, and no antibodies to VRC01LS were detected.

This Phase I study design includes 14 volunteers who received a single infusion of VRC01LS (at 5, 20, or 40 mg/kg) and 23 volunteers who received three infusions of VRC01LS 12 weeks apart. For PK analysis, we focused on analyzing the first VRC01LS infusion either from volunteers scheduled to receive only one infusion or from the first infusion from volunteers scheduled to receive multiple doses, in part because the unusually long half-life of this antibody, together with 12-week intervals between administration, require an extended observation time to accurately assess PK in the multiple-dose arms of the study. From this initial analysis, the half-life was 71 days—more than fourfold longer than the previously published wild-type VRC01 antibody half-life. This prolonged half-life was observed at each dose level and with SC administration. Although SC administration resulted in lower peak serum concentrations than IV administration, the serum concentration at week 4 was similar in the groups receiving 5 mg/kg IV and SC infusions. While the serum concentration needed to prevent HIV-1 infection in humans is not yet known, preclinical studies in NHPs suggest that levels above 10 μg/mL of VRC01 can be protective, although this value depends on several variables, including the virus used as part of the challenge experiment [11,30,37,38]. In this context, we observed that a dose of 5 mg/kg, which can be given by SC injection, produced serum levels above 10 μg/mL for up to 24 weeks.

Increased serum antibody concentration represents an important clinical metric when considering HIV-1 infection prevention potential but may not be the sole factor of protection. Tissue levels may also be important. While we did not assess tissue levels of VRC01LS in this study, such a Phase I clinical study is underway (clinicaltrials.gov NCT02797171). In NHPs, we previously demonstrated that the FcRn is present in mucosal tissue (both rectal and vaginal) and that tissue levels of VRC01LS persisted far longer than did those for VRC01 [30]. In an NHP challenge study directly comparing the protection afforded by a single IV infusion of VRC01 and VRC01LS, Gautam and colleagues showed that VRC01LS provided a significantly longer duration of protection. That study [39], as well as our Phase I study here, confirm that VRC01LS retains its full neutralizing activity in serum, even many weeks after infusion. Taken together, results from NHP challenge studies and data from this study offer evidence that VRC01LS produces an extended duration of physiologically relevant levels of antibody and represents a significant improvement over VRC01 in terms of HIV-1 prevention potential.

The PK results that we report from an LS mutation are comparable to PK alterations previously documented in antibodies with the YTE mutation. Motavizumab-YTE was first demonstrated to have a half-life three to four times longer than wild-type motavizumab in monkeys, which was later retained in studies involving humans [28]. MEDI8897, which also contains the YTE change, similarly has a half-life three to four times longer than wild-type MEDI8897 [29]. One distinguishing characteristic of the LS mutation compared to YTE, however, is the effect on binding to Fc-gamma. The YTE mutation reduces affinity for Fc-gamma and thus results in diminished ADCC [26,30]. The LS mutation does not significantly impact Fc-gamma binding and thus retains the Fc-mediated ADCC function of the antibody [30].

There are several potential limitations of the VRC 606 trial. It is a small study typical of Phase I trials, and therefore the conclusions drawn will require further prospective validation. This was the experience of early trials for VRC01, which have since shown similar results in expanded Phase I trials [40,41]. Additionally, this report focuses on data acquired after a single administration of VRC01LS. Data from multiple administration groups will require further reporting, and these data will also require replication in separate Phase I trials. Finally, the potential clinical impact of the extended half-life antibodies will depend on an efficacy signal established by Phase IIb efficacy trials of VRC01.

The increased durability of VRC01LS in serum could allow for administration at intervals of 3–6 months. This will depend, in part, on what is learned from an ongoing study (clinicaltrials.gov NCT02716675 and NCT02568215), in which VRC01 is being administered at either 10 or 30 mg/kg by IV infusion every 8 weeks. Notably, mAbs more potent than VRC01 are under development and, together with long half-life extension mutations, the goal of next-generation antibody products would be SC administration 2–4 times per year to maintain protective antibody levels. Such a goal may be met by using a combination of two mAbs or a single bi- or tri-specific antibody [42] to provide breadth of reactivity to the large majority of diverse HIV-1 strains. Notably, antibody prophylaxis during a finite period of risk, such as breastfeeding, would be faciliated with less frequent dosing. The extended duration would also simplify dosing regimens for persons with ongoing risk, such as HIV-discordant couples seeking to avoid sexual transmission. It would also provide advantages to groups who traditionally have difficulty with adherence to daily medication regimens. HIV-1 mAbs provided every three months could be more readily incorporated into existing care models for other extended-dosing injectable medications, such as women who receive medroxyprogesterone (Depo-Provera) for birth control. In summary, the present study suggests that if VRC01 is shown to be protective in ongoing efficacy trials, then VRC01LS or other long half-life mAbs with improved breadth and potency could provide improved efficacy with lower mAb amounts and less frequent dosing.

## Supporting information

S1 CONSORT Checklist CONSORT checklist. (DOC)

S1 Text Trial protocol. (PDF)

## Abbreviations

ADA: antidrug antibody
ADCC: antibody dependent cellular cytotoxicity
AE: adverse event
AUC: area under the curve
bnAb: broadly neutralizing antibody
bnMAb: broadly neutralizing monoclonal antibody
cGMP: current Good Manufacturing Practice
CHO: Chinese hamster ovary
CL: clearance
C_max_: maximal concentration
ECL: electrochemiluminescence
EIA: enzyme immunoassay
Env: envelope glycoprotein
gp: glycoprotein
Fab: antigen-binding fragment
Fc: constant region
FcRn: neonatal Fc receptor
GM: genetic marker
IC_80_: 80% inhibitory concentration
ID_80_: dilution that produced 80% neutralization
IgG: immunoglobulin G
IgG1: immunoglobulin G subclass 1
IV: intravenous
mAb: monoclonal antibody
MedDRA: Medical Dictionary for Regulatory Activities
MPER: membrane-proximal external region
MSD: Meso Scale Discovery
NHP: nonhuman primate
NIAID: National Institute of Allergy and Infectious Diseases
NIH: National Institutes of Health
PK: pharmacokinetics
RSV: respiratory syncytial virus
SAE: serious adverse event
SC: subcutaneous
s.d.: standard deviation
T_max_: time to maximal concentration
TMB: tetramethylbenzidine
t_1/2β_: elimination half-life
VCMP: Vaccine Clinical Materials Program
VRC: Vaccine Research Center
V1V2: variable region 1 and 2
