# Supplementary material for: Safety and pharmacokinetics of the Fc-modified HIV-1 human monoclonal antibody VRC01LS: A Phase 1 open-label clinical trial in healthy adults
Source: PLoS Med. 2018 Jan 24;15(1):e1002493. doi: 10.1371/journal.pmed.1002493 (PMC5783347; doi:10.1371/journal.pmed.1002493)
Supplement: S1 Text — (PDF) [file pmed.1002493.s002.pdf]

Version 2.0
November 12, 2016

VACCINE RESEARCH CENTER

Protocol VRC 606
(NIH 16-I-0018)
(DAIDS-ES ID #12043)

A PHASE 1, DOSE-ESCALATION STUDY OF THE SAFETY AND PHARMACOKINETICS OF A HUMAN MONOCLONAL ANTIBODY, VRC-HIVMAB080-00-AB (VRC01LS), AND VRC-HIVMAB060-00-AB (VRC01), ADMINISTERED INTRAVENOUSLY OR SUBCUTANEOUSLY TO HEALTHY ADULTS

Study Agent Provided by
Vaccine Research Center/NIAID/NIH, Bethesda, MD

Clinical Trial Sponsored by:
National Institute of Allergy and Infectious Diseases (NIAID)
Vaccine Research Center (VRC)
Bethesda, Maryland

IND Sponsored by:
National Institute of Allergy and Infectious Diseases
Division of AIDS (DAIDS)
Bethesda, Maryland

IND 125,494 - held by DAIDS

Principal Investigator:
Julie E. Ledgerwood, D.O.
Vaccine Research Center, National Institute of Allergy and Infectious Diseases (NIAID)
National Institutes of Health (NIH)
Bethesda, MD 20892

IRB Initial Review Date: September 28, 2015

TABLE OF CONTENTS

Page

ABBREVIATIONS **5**

PRÉCIS **7**

1. INTRODUCTION **8**

1.1 Rationale for the Study 8
1.2 Comparability VRC01LS and VRC01 9
1.3 VRC01LS and VRC01 Specific Laboratory Assessments 10
1.4 Previous human experience 10
1.4.1 VRC01LS Safety Data 10
1.4.2 VRC01 Safety Data 11
1.4.3 Antiviral Effect of VRC01 12
1.5 Pharmacokinetic Parameters 12

2. STUDY AGENTS **13**

2.1 VRC-HIVMAB080-00-AB 13
2.2 VRC-HIVMAB060-00-AB 13
2.3 Preclinical Safety Studies 14
2.4 Nonhuman Primate (NHP) Studies 14

3. STUDY OBJECTIVES **14**

3.1 Primary Objectives 14
3.2 Secondary Objectives 14
3.3 Exploratory Objectives 15

4. STUDY DESIGN **15**

4.1 Study Population 16
4.1.1 Inclusion Criteria 16
4.1.2 Exclusion Criteria 17
4.2 Clinical Procedures and Laboratory Assays 17
4.2.1 Screening 17
4.2.2 Enrollment, Study Days and Visit Numbers 17
4.2.3 Product Administration 18
4.2.4 Solicited Adverse Events and Clinical Follow-up 18
4.2.5 Pharmacokinetics Procedures 19
4.2.6 Mucosal Samples 19
4.2.7 Schedule of Evaluations 20
4.2.8 Concomitant Medications 20
4.3 Criteria for Dose Escalation 20
4.4 Discontinuation of Product Administration 21
4.5 Protocol Criteria for Pausing the Study and Resuming the Study 21

5. SAFETY AND ADVERSE EVENT REPORTING **22**

5.1 Adverse Events 22
5.2 Serious Adverse Events (SAE) 22

5.3 Adverse Event Reporting to the IND Sponsor ... 23
5.3.1 Expedited Adverse Event (EAE) Reporting Criteria ... 23
5.3.2 Attribution Categories ... 24
5.4 Reporting to the Institutional Review Board ... 24
5.4.1 Unanticipated Problem (UP) Definition ... 24
5.4.2 Protocol Deviation Definition ... 25
5.4.3 Non-Compliance Definition ... 25
5.4.4 Expedited Reporting to the NIAID IRB ... 25
5.4.5 Annual Reporting to the NIAID IRB ... 26

6. STATISTICAL CONSIDERATIONS ... 26

6.1 Overview ... 26
6.2 Objectives ... 26
6.3 Size and Accrual ... 26
6.3.1 Randomization of Treatment Assignments ... 27
6.3.2 Sample Size Considerations ... 27
6.4 Statistical Analysis ... 28
6.4.1 Analysis Variables ... 28
6.4.2 Baseline Demographics ... 28
6.4.3 Safety Analysis ... 29
6.4.4 Tolerability Evaluation ... 29
6.4.5 Pharmacokinetics Analysis ... 29
6.4.6 Interim Analyses ... 30

7. PHARMACY PROCEDURES ... 30

7.1 Study Products and Administration Regimen ... 30
7.2 Study Product Storage ... 31
7.3 Preparation of Study Products for Administration ... 31
7.3.1 VRC-HIVMAB080-00-AB and VRC-HIVMAB060-00-AB: Preparation for Administration Intravenously ... 32
7.3.2 VRC-HIVMAB080-00-AB and VRC-HIVMAB060-00-AB: Preparation for Administration Subcutaneously ... 32
7.4 Labeling of Study Agents ... 33
7.5 Study Agent Accountability ... 33
7.6 Study Agent Disposition ... 33

8. HUMAN SUBJECT PROTECTIONS AND ETHICAL OBLIGATIONS ... 33

8.1 Informed Consent ... 33
8.2 Risks and Benefits ... 33
8.2.1 Risks ... 33
8.2.2 Benefits ... 35
8.3 Institutional Review Board ... 35
8.4 Protocol Registration ... 35
8.5 Subject Confidentiality ... 36
8.6 Plan for Use and Storage of Biological Samples ... 36
8.6.1 Use of Samples, Specimens and Data ... 36
8.6.2 Storage and Tracking of Blood Samples and Other Specimens ... 36

8.6.3 Disposition of Samples, Specimens and Data at Completion of the Protocol 36
8.6.4 Loss or Destruction of Samples, Specimens or Data 37
8.7 Subject Identification and Enrollment of Study Participants... 37
8.7.1 Participation of Children.. 37
8.7.2 Participation of NIH Employees 37
8.8 Compensation 37
8.9 Safety Monitoring 38

9. ADMINISTRATIVE AND LEGAL OBLIGATIONS 38

9.1 Protocol Amendments and Study Termination.. 38
9.2 Study Documentation and Storage... 38
9.3 Study Monitoring, Data Collection and Data Monitoring 39
9.3.1 Study Monitoring 39
9.3.2 Data Collection.. 39
9.4 Language.. 39
9.5 Policy Regarding Research-Related Injuries 39

10. REFERENCES.. 40

APPENDICES

I Study Informed Consent Form 43
II Contact Information 54
III Schedule of Evaluations 56
IV Table for Grading Severity of Adverse Events 64

ABBREVIATIONS

Abbreviation	Term
ADA	anti-drug antibody
ADL	activities of daily living
AE	adverse event
AIDS	Acquired Immunodeficiency Syndrome
ALP	alkaline phosphatase
ALT	alanine aminotransferase
AoU	Assessment of Understanding
ART	antiretroviral therapy
AST	aspartate aminotransferase
AUC	area under the curve
β-HCG	human chorionic gonadotropin
BMI	body mass index
CBC	complete blood count
CCR5	CC family of chemokines, receptor type 5
CL	clearance
Cmax	maximum concentration
CRS	cytokine release syndrome
cGMP	current Good Manufacturing Practice
DAERS	DAIDS Adverse Event Reporting System
DAIDS	Division of AIDS
DNA	deoxyribonucleic acid
DSMB	Data and Safety Monitoring Board
EAE	expedited adverse event
EC50	Half-maximal effective concentration
EDTA	Ethylenediaminetetraacetate
ELISA	enzyme-linked immunosorbent assay
Env	envelope
EOI	end of infusion
F	bioavailability
FDA	Food and Drug Administration
GCP	Good Clinical Practice
GLT	(green) lithium heparin tube
HIV	human immunodeficiency virus
HLA	human leukocyte antigen
HRPP	Human Research Protections Program
IB	Investigator's Brochure
IgG1	Immunoglobulin G1
IND	investigational new drug application
IRB	Institutional Review Board
IV	intravenous
kg	kilogram
L	liter

Abbreviation	Term
LIMS	Laboratory Information Management System
λz	terminal slope of concentration vs time profile
MAb	monoclonal antibody
mcg	microgram
mg	milligram
mL	milliliter
mM, mmol	millimole
MSD	Meso Scale Discovery
NIAID	National Institute of Allergy and Infectious Diseases
NIH	National Institutes of Health
NIH CC	National Institutes of Health Clinical Center
NHP	Non-human primate
NVITAL	NIAID Vaccine Immune T-Cell and Antibody Laboratory
OHRP	Office for Human Research Protections
PBMC	peripheral blood mononuclear cells
PBS	phosphate buffered saline
PCR	polymerase chain reaction
PI	Principal Investigator
PK	pharmacokinetic
PSRT	Protocol Safety Review Team
Q	Inter-compartmental clearance
QA	quality assurance
RSC	Regulatory Support Center
SAE	serious adverse event
SC	subcutaneous
SHIV	simian-human immunodeficiency virus
SST	serum separator tube
TCR	tissue cross reactivity
$T_{1/2}$	half-life
Tmax	time of maximal concentration (Cmax)
UNAIDS	Joint United Nations Programme on HIV/AIDS
UP	unanticipated problem
USP	United States Pharmacopeia
Vd	volume of distribution
VRC	Vaccine Research Center
WBC	white blood cell

PRÉCIS

VRC 606: A Phase 1, Dose-Escalation Study of the Safety and Pharmacokinetics of a Human Monoclonal Antibody, VRC-HIVMAB080-00-AB (VRC01LS), and VRC-HIVMAB060-00-AB (VRC01), Administered Intravenously or Subcutaneously to Healthy Adults

Study Design: This is the first study of the VRC-HIVMAB080-00-AB (VRC01LS) monoclonal antibody (MAb) in healthy adults. It is a dose-escalation study to examine safety, tolerability, dose, and pharmacokinetics of VRC01LS. The hypothesis is that VRC01LS will be safe to administer to healthy adults by the intravenous (IV) and subcutaneous (SC) routes. The secondary hypothesis is that VRC01LS will be detectable in human sera with a definable half-life. With protocol amendment, Group 7 and Group 8 were added to the protocol Version 2.0 to evaluate VRC01 and VRC01LS safety and pharmacokinetics in the same study.

Product Description: VRC-HIVMAB080-00-AB (VRC01LS) and VRC-HIVMAB060-00-AB (VRC01) are human MAb targeted to the CD4 binding site of HIV-1. VRC01LS is a modification of the VRC01 MAb (which has been shown to be safe in human studies) with the addition of the “LS”, 2-amino acid mutation designed to improve the half-life of the antibody. VRC01LS and VRC01 were developed and manufactured by VRC/NIAID/NIH under cGMP at the VRC Pilot Plant operated under contract by the Vaccine Clinical Materials Program (VCMP), Leidos Biomedical Research, Inc., Frederick, MD. Vials are provided at 100 mg/mL.

Subjects: Healthy adults, 18-50 years of age.

Study Plan: There are 4 open-label, dose escalation groups (Groups 1-4) to assess VRC01LS administered IV and SC once per subject, 2 open-label groups (Groups 5 and 6) to assess VRC01LS at 5 mg/kg SC or at 20 mg/kg IV administered every 12 weeks for a total of 3 administrations per subject, and 2 open-label groups (Groups 7 and 8) to assess VRC01 at 5 mg/kg SC or at 20 mg/kg IV administered every 4 weeks for a total of 2 administrations per subject. Subjects will be randomized into Group 1 or Group 2 at a 1:1 ratio, or directly enrolled into Groups 3-6 per the dose escalation and safety evaluation plan. Enrollments into Group 7 and Group 8 will be randomized at a 1:1 ratio.

VRC 606 Study Schema

Group	Subjects	Product	Administration Schedule			
			Day 0	Week 4	Week 12	Week 24
1	3	VRC01LS	5 mg/kg IV			
2	3	VRC01LS	5 mg/kg SC			
3	3	VRC01LS	20 mg/kg IV			
4	5	VRC01LS	40 mg/kg IV			
5	15	VRC01LS	5 mg/kg SC		5 mg/kg SC	5 mg/kg SC
6	10	VRC01LS	20 mg/kg IV		20 mg/kg IV	20 mg/kg IV
7	5	VRC01	5 mg/kg SC	5 mg/kg SC		
8	5	VRC01	20 mg/kg IV	20 mg/kg IV		
Total*	49					

*Enrollment up to a total of 60 subjects is permitted in case there are subjects who do not complete the schedule, if additional PK evaluations are needed, or if an enrollment of additional subjects is necessary for safety evaluations (Section 4.3).

Study Duration: Subjects will be followed for 24 weeks after the last study product administration.

1. INTRODUCTION

The global incidence of new human immunodeficiency virus (HIV) infection peaked in the mid-1990s. The incidence of new infections in 2014 is reported by Joint United Nations Programme on HIV/AIDS (UNAIDS) as 2 million new cases, down from 2.9 million in 2005, with an estimated global total of 36.9 million people living with HIV. The reduction of HIV incidence is due to multiple factors that include prevention and treatment programs. The decrease in incidence is an encouraging trend, but the scope and cost of the epidemic remains of great global concern. The wider availability of antiretroviral therapy (ART), mother to child transmission prevention programs, and a diverse array of other prevention programs have all contributed to turning the tide of the epidemic [1]. Each effective form of prevention and treatment is a welcome public health measure.

The National Institute of Allergy and Infectious Diseases (NIAID), National Institutes of Health (NIH) is committed to the development of safe, effective methods to prevent and treat HIV infection and AIDS worldwide. In this regard, the Vaccine Research Center (VRC), NIAID and Division of AIDS (DAIDS), NIAID, are collaborating to evaluate the potential clinical uses of HIV-specific broadly neutralizing human monoclonal antibodies (MAb) [2-4].

The VRC, NIAID, NIH developed VRC01LS, a new generation of highly potent and broadly neutralizing HIV-1 human MAb targeted against the HIV-1 CD4 binding site [5]. The predecessor, VRC01 MAb, currently in clinical trials under IND 113611 for prevention indication and IND 126001 for therapeutic indication, was originally discovered in a subject infected with HIV-1 for more than 15 years and whose immune system controlled the virus without anti-retroviral therapy [6]. VRC01 sequence was modified by site-directed mutagenesis to increase its binding affinity for the neonatal Fc receptor (FcRn) and the resulting antibody was designated VRC01LS. The LS designation specifies methionine to leucine (L) and asparagine to serine (S) (M428L/N434S, referred to as LS) changes within the C-terminus of the heavy chain constant region far outside of the antigen-binding site [7]. Other than the two amino acid difference, VRC01LS is identical to VRC01. As a result of its enhanced FcRn function, VRC01LS has an extended half-life in both serum and mucosal tissue compared to VRC01 and improved protection against primate SHIV infection [5].

VRC-HIVMAB080-00-AB (VRC01LS) is an investigational drug which has not been administered to humans prior to this study. VRC01LS is intended for the prevention of HIV-1 infection in healthy adults initially and later for the prevention of HIV-1 infection in adolescents and in infants at risk for HIV-1 infection through maternal transmission at birth or during breastfeeding. Also VRC01LS may be evaluated for treatment of HIV-1 infected subjects.

1.1 RATIONALE FOR THE STUDY

The initial clinical development proposal for VRC01 is derived from a series of consultations conducted by VRC/NIAID/NIH, including a convened panel of experts that occurred in July 2010, regarding the potential clinical applications of VRC01 and other neutralizing MAb. VRC considers the potential clinical uses for these MAb in three broad areas: 1) prevention of transmission from HIV-1 infected mothers to newborn and breastfeeding infants, 2) prevention of HIV infection by sexual transmission, and 3) therapeutic application in HIV-1 infected individuals. The initial development plan was focused on clinical research that would lead to an efficacy evaluation for the prevention of transmission from HIV-infected mothers to newborn and breastfeeding infants. In

order to evaluate the safety of administering the VRC01 to infants who may or may not be HIV-infected at birth, the studies in infants were preceded by Phase 1 trials in HIV-infected adults (VRC 601) and uninfected adults (VRC 602) that began in 2013 and 2014 respectively.

In the VRC 601 and VRC 602 studies, VRC01 has been assessed as safe and well tolerated at the 5-40 mg/kg dosages administered intravenously (IV) and at 5 mg/kg subcutaneously (SC) in both HIV-infected and HIV-uninfected adult populations. The pharmacokinetic (PK) parameters of passively administered VRC01 have been evaluated. For healthy adults who received VRC01 at the 5-40 mg/kg dosages administered IV (n=18), the clearance was 0.016±0.003 L/h and an overall mean value for the elimination half-life was 15.4±3.9 days for IV administration. At 5 mg/kg SC (n=5), the clearance was 0.029±0.007 L/h, and the mean elimination half-life was 16.6 ± 2.9 days [8]. In addition, VRC01 demonstrated antiviral activity when administered at a single 40 mg/kg IV dose to HIV-1 infected subjects (Section 1.4.3). Work is ongoing to further describe the PK and biological activity of VRC01 after repeat dosing in healthy and HIV-infected adults.

Prolonged half-life for a MAb may allow less frequent administrations while still keeping biological activity. In animal studies, VRC01LS showed a 2.5- to 3-fold increase in half-life in macaques and similar *in vitro* neutralization potency and breadth when compared to VRC01, and it persisted in the rectal mucosa even when it was no longer detectable in the serum.

When administered at a single dose of 10 mg/kg IV to rhesus macaques, VRC01LS persisted up to 70 days in rectal tissues while VRC01 was no longer detectable after 28 days. When administered at a single 'sub-optimal' dose of 0.3 mg/kg IV, VRC01LS was more effective than VRC01 in protecting rhesus macaques from SHIV infection and was found at higher concentrations in rectal tissues [3, 5].

Similarly to VRC01, VRC01LS does not react with phospholipids or nuclear antigens and does not bind to HEp-2 human epithelial cells, therefore demonstrating a lack of autoreactivity as tested *in vitro*. This study will inform the product safety in healthy adults and will assess for the presence of anti-VRC01LS antibody following repeat dosing.

The increased half-life of VRC01LS and persistence at higher concentrations in mucosal tissues correlate with improved protection against SHIV infection *in vivo* in animal studies suggesting a potential clinical application for the prevention or treatment of HIV-1 infection in humans.

The proposed VRC01LS dosages are based on dosages evaluated in VRC studies of VRC01. Preclinical studies with VRC01LS to date also support the proposed human dosages and intervals between doses to achieve passive immunity with VRC01LS as described in Section 2.3.

With the protocol Version 2.0 amendment, Group 7 and Group 8 were added with the goal to evaluate VRC01 and VRC01LS safety and pharmacokinetics in the same study. The doses and administration schedules for Groups 7 and 8 were selected based on the VRC01 PK data [8].

1.2 COMPARABILITY VRC01LS AND VRC01

The molecular characteristics, quality and physical attributes of VRC01LS were compared to VRC01 by testing for anti-phospholipid reactivity, anti-nuclear antigen reactivity, binding to human epithelial cell line (HEp-2), tissue cross reactivity (TCR), and neutralization activity against a panel of HIV isolates. With exception of the extended half-life transferred by the LS amino acid substitutions, the two antibodies, VRC01LS and VRC01, are similar. More details on the comparability testing can be found in Section 2.2 and in the Investigator's Brochure (IB).

1.3 VRC01LS AND VRC01 SPECIFIC LABORATORY ASSESSMENTS

Laboratory assessments in the Phase 1 studies of the VRC01LS and VRC01 MAb include pharmacokinetics (PK), assessment for the development of anti-drug antibody (ADA) following exposure to the product, and functional capacity (neutralization) of the MAb following infusion.

VRC01LS and VRC01 concentration for the PK analysis in this Phase 1 study will be measured by an ELISA using the VRC01 anti-idiotype, Fab-specific 5C9 monoclonal antibody. To create this antibody, B cells were obtained from a mouse immunized with VRC01 Fab, and 5C9 was cloned from a single B cell that was sorted by flow cytometry using the VRC01 scFv probe. The 4-parameter logistic curve regression of a standard curve of VRC01 covering the range from 0.031 to 1.0 mcg/mL is utilized in this assay to quantitate the sample concentrations based upon the average of sample dilutions within the range of the assay.

Assessment for development of ADA in subjects will be performed using the Meso Scale Discovery (MSD) platform based on electrochemiluminescence. The developed ADA assay uses the biotin-labeled VRC01LS or VRC01 immobilized on a streptavidin-coated MSD plate as the capture molecule, and the SULFO-TAG labeled VRC01LS or VRC01 as the reporter molecule. This assay is independent of the ADA isotype, and permits the detection of both high and low affinity antibodies. Additional testing may be conducted with VRC01-based assay if positive ADA is detected to VRC01LS. Evaluation for ADA will be conducted in batches, on samples collected at 4 weeks after each antibody administration. Samples collected at other visits could be tested if there is a clinical indication or a decrease in PK values observed.

Depending upon the concentrations measured in collected specimens, further evaluation of the research samples to assess for functional capacity to neutralize HIV may be conducted by an *in vitro* cell-based virus neutralization assay using the pseudotyped viruses [9-11].

As an exploratory evaluation, subjects may be evaluated for their IgG1 allotypes to determine the potential for theoretical allotype-specific effects on the VRC01LS and VRC01 pharmacokinetics such as reduced half-life or anti-drug antibody response [12-14]. Coded stored samples will be used for evaluation of the genetic sequence of the immunoglobulin heavy chain constant region allotype.

1.4 PREVIOUS HUMAN EXPERIENCE

1.4.1 VRC01LS Safety Data

There was no previous human experience with VRC01LS administration until VRC 606 was opened for accrual and the first subject was enrolled on 11/16/2015. As of 8/15/2016, 30 subjects have been enrolled and received one or more administrations of VRC01LS; 11 subjects completed study participation and 19 subjects completed product administration on the study. Overall, 45 product administrations have been completed, including 25 IV infusions and 20 SC injections.

There have been no serious adverse events (SAEs). Overall, 20 of 30 subjects (66.7%) have had one or more unsolicited adverse events (AEs), with maximum severity being Grade 1 for 11 subjects and Grade 2 for 9 subjects. Four AEs were assessed as related to study product: 2 instances of Grade 1 diarrhea on the day of product administration (Group 1, 5 mg/kg IV; Group 5, 5 mg/kg SC) and resolved on the same day, a Grade 1 dizziness one day after product administration (Group 2, 5 mg/kg SC) that resolved within 10 minutes, and a Grade 1 injection site reaction manifested as induration and swelling at 14 days post product administration (Group 5, 5 mg/kg SC). The injection site reaction resolved at 28 days post administration with sequela of post-inflammatory

hyperpigmentation that is expected to resolve over several months. By the Principal Investigator's (PI) discretion, further injections will not occur and the subject will be followed for safety evaluations.

Product administrations have been generally well tolerated. Overall, 3 of 17 subjects (17.6 %) who received VRC01LS IV and 9 of 11 subjects (81.8%) who received VRC01LS SC and completed diary cards reported solicited local reactions in the week after product administration. These include 8 reports of mild local pain/tenderness by: 1 subject in Group 3 (20 mg/kg, IV), 1 subject in Group 6 (20 mg/kg, IV), by 2 subjects in Group 2 (5 mg/kg, SC), and 4 subjects in Group 5 (5 mg/kg, SC). One subject in Group 5 (5 mg/kg, SC) reported moderate pain, 2 subjects (Group 6, 20 mg/kg IV; Group 2, 5 mg/kg, SC) reported mild bruising, 2 subjects in Group 5 (5 mg/kg SC) reported mild swelling, and 2 subjects (Group 2 and 5, 5 mg/kg SC) reported mild redness.

Observations during product administration included brief reactions of local pain and/or stinging sensations at SC administration sites (12 of 13 subjects, 92.3%) that resolved within 2-5 minutes after injection. These reactions were consistent with known risks of injections and did not meet criteria for reporting as AEs as defined by the Table for Grading Severity of Adverse Events (Appendix IV of the protocol).

With regard to solicited systemic adverse events, 9 of 28 (32.1%) had one or more mild systemic signs or symptoms in the 3 days after product administration. This includes mild malaise (n=6), myalgia (n=5), headache (n=3), and nausea (n=3).

No systemic symptoms were reported during product administration.

1.4.2 VRC01 Safety Data

The antibody VRC01 has been tested in HIV-infected adults (VRC 601, A4340, A5342, and 15-I-0140 studies), in healthy adults (VRC 602, HVTN 104, HVTN 704/HPTN 085, HVTN 703/ HPTN 081 studies), and in infants (P1112). Data on safety, pharmacokinetics and neutralization of VRC01 in HIV infected adults (VRC 601 study, [15]) and in healthy adults (VRC 602 study, [8]) have been summarized in recent publications.

As of July 27, 2016, under the studies described above, approximately 350 adult subjects and 21 HIV uninfected infants have received one or more VRC01 administrations. The number of VRC01 administrations per subject has ranged from 1 to 12, with the 12 administrations for subjects on a schedule that starts with an IV infusion followed by 11 SC administrations at 2 week intervals in HVTN 104.

Cumulatively across all studies to date, there have been no serious adverse events related to VRC01 that required expedited reporting to the Food and Drug Administration (FDA) or other regulatory authorities, no study safety pauses for adverse events. There have been no reactions during the VRC01 administration that resulted in an incomplete administration.

The VRC01 SC administrations were generally associated with mild local reactions during the administration that may include some pruritus (itchiness), redness and swelling, which resolves within a few minutes to a few hours after the administration is completed. Erythema/induration reactions were reported rarely; the largest diameter for erythema or swelling events that were observed during infusions ranged up to about 9 cm. One subject in VRC 602 receiving placebo SC reported mild flushing during administration and one subject in VRC 601 (5 mg/kg SC) reported mild nausea during VRC01 SC administration.

The solicited local and systemic signs and symptoms following administration of VRC01 are generally none to mild. Less than 20% of subjects have reported any moderate or greater solicited reactions after product administration.

There have been no grade 3 or higher AEs attributed to VRC01 administration. Adverse events attributed to study product administration on the basis of temporal relationship have included AST, ALT and creatinine elevation, decreased neutrophil count, diarrhea, herpes zoster, and pruritus at the administration site. These laboratory changes and events have generally been attributed to study product due to temporal relationship, have resolved without clinical sequela, and did not require discontinuation of study product administration.

Adverse events attributed to study product administration (VRC01 or placebo) on the basis of temporal relationship for which the schedule of study product administration was discontinued included one subject with mild chest discomfort and one subject with mild rash in HVTN 104 study. Both AEs resolved without clinical sequela.

Overall, VRC01 infusions in the dose range from 1 to 40 mg/kg IV and at 5 and 40 mg/kg SC have been assessed as well-tolerated and safe for further evaluation.

1.4.3 Antiviral Effect of VRC01

Analysis of the VRC 601 viral load data obtained from 8 viremic adults shows that VRC01 has a statistically significant *in vivo* virological effect on HIV viral load when administered as a single 40 mg/kg IV dose. None of these adults were taking antiretroviral therapy (ART) when enrolled into the study and had not started ART during the time period when the viral load data were collected. Six of the eight adult subjects had ≥1 log_{10} copies/mL decrease in viral load and two subjects had a viral load drop of 0.26 and 0.18 log_{10} copies/mL respectively.

These data indicate the following for a single dose of VRC01 at 40 mg/kg IV:

- A statistically significant change from baseline viral load post-infusion days 5 to 16;
- The median time to reach ≥0.5 log_{10} decrease in viral load is 5 days; and,
- The median time to greatest decrease in viral load is 7 days.

A 0.5 log_{10} copies/mL or greater decrease in viral load is considered to be a positive response to ART [16]. To have clinical benefit, such a change would need to be sustained. In VRC 601, subjects were administered only one dose of VRC01 at 40 mg/kg and, thus, a sustained effect on viral load was not expected. However, the data demonstrate a VRC01 mediated anti-viral effect and that the established benchmarks can be obtained, and support the hypothesis that a schedule with repetitive dosing may have a beneficial clinical effect.

To advance the product development, several studies for VRC01 evaluation in healthy and HIV infected adults are in planning stages or recently began subject enrollment. A first study of VRC01 in infants at risk of mother to child HIV transmission (IMPAACT P1112) was open to accrual in April 2015.

1.5 PHARMACOKINETIC PARAMETERS

The pharmacokinetic (PK) parameters of the passively administered, predecessor MAb VRC01 have been evaluated in a limited number of healthy and HIV-infected adults and can be found in the IB. Work is ongoing to further describe the PK of VRC01 by the IV and SC routes of administration after a single dose and a repeat dosing in adults.

Based on animal studies, VRC01LS has a 2.5- to 3-fold increase in half-life in macaques as compared to VRC01 [5]. The PK parameters of VRC01LS in humans will be evaluated in this study.

2. STUDY AGENTS

The study agents, VRC-HIVMAB080-00-AB (VRC01LS) and VRC-HIVMAB060-00-AB (VRC01) were produced under current Good Manufacturing Practice (cGMP) by VRC/NIAID/NIH at the VRC Pilot Plant operated under contract by the Vaccine Clinical Materials Program (VCMP), Leidos Biomedical Research, Inc., Frederick, MD. Specific manufacturing information is included on the product vial labels and Certificates of Analysis, and can be found in the IB. Quality Assurance (QA) lot release testing by the manufacturer and ongoing stability programs verify conformance to product specifications prior to and throughout use in clinical trials.

2.1 VRC-HIVMAB080-00-AB

VRC-HIVMAB080-00-AB (VRC01LS) is a broadly neutralizing human MAb targeted against the HIV-1 CD4 binding site. It was developed by VRC/NIAID/NIH.

VRC01 was modified by site-directed mutagenesis to increase its binding affinity for the neonatal Fc receptor (FcRn). The resulting antibody is designated VRC01LS. The LS designation specifies methionine to leucine (L) and asparagine to serine (S) (M428L/N434S, referred to as LS) changes within the C-terminus of the heavy chain constant region far outside of the antigen-combining site [7]. The VRC01LS is an IgG1, and the glycosylation pattern is derived from its production in a Chinese Hamster Ovary (CHO) mammalian cell line.

Drug substance was manufactured under cGMP using a stable transfected CHO cell line, purified, and the drug product vials were filled and labeled. Each product vial contains 6.25 mL volume at a concentration of 100 mg/mL VRC01LS in formulation buffer containing 25 mM Sodium Citrate, 50 mM Sodium Chloride, and 150 mM L-Arginine Hydrochloride at pH 5.8.

More details on the VRC-HIVMAB080-00-AB composition and manufacturing can be found in the IB.

2.2 VRC-HIVMAB060-00-AB

VRC-HIVMAB060-00-AB (VRC01) is a broadly neutralizing human MAb targeted against the HIV-1 CD4 binding site. It was developed by VRC/NIAID/NIH. VRC01 is an IgG1 and is highly somatically mutated from the germ-line precursor. The heavy chain CDR3 region is 14 amino acids long, which is an average length relative to natural antibodies, and the glycosylation pattern is derived from its production in a CHO mammalian cell line.

Drug substance was manufactured under cGMP using a stable CHO cell line, purified, and the drug product vials were filled and labeled. Each vial contains 6.25 mL ± 0.10 mL at a concentration of 100 ± 10 mg/mL in formulation buffer containing 25 mM Sodium Citrate, 50 mM Sodium Chloride, and 150 mM L-Arginine Hydrochloride at pH 5.8.

More details on the VRC-HIVMAB060-00-AB composition and manufacturing can be found in the product's IB.

2.3 Preclinical Safety Studies

The *in vitro* preclinical safety studies have been performed to assess potential off target binding by VRC01 and VRC01LS. VRC01LS and VRC01 were compared in a non-GLP tissue cross reactivity (TCR) study using research-grade materials and tissues from a single adult donor. VRC01LS and VRC01 showed nearly identical minimal tissue binding; similar results were reported in the GLP TCR study performed for VRC01 [IND 113,611]. There was no unexpected off target binding.

More information on these and other preclinical studies with VRC01 and VRC01LS can be found in the products' IBs.

2.4 Nonhuman Primate (NHP) Studies

Several non-GLP studies of VRC01LS and VRC01 have been completed in NHP to assess for preclinical evidence of potential efficacy for prevention of HIV infection. More information on these studies can be found in the products' IBs.

3. STUDY OBJECTIVES

3.1 Primary Objectives

- To evaluate the safety and tolerability of VRC-HIVMAB080-00-AB (VRC01LS) administered as a single dose at 5 mg/kg IV, 20 mg/kg IV, 40 mg/kg IV, and 5 mg/kg SC to healthy adults.
- To evaluate the safety and tolerability of VRC-HIVMAB080-00-AB (VRC01LS) administered at 20 mg/kg IV by repeat dosing every 12 weeks for a total of 3 infusions to healthy adults.
- To evaluate the safety and tolerability of VRC-HIVMAB080-00-AB (VRC01LS) administered at 5 mg/kg SC by repeat dosing every 12 weeks for a total of 3 injections to healthy adults.
- To evaluate the safety and tolerability of VRC-HIVMAB060-00-AB (VRC01) administered at 5 mg/kg SC by repeat dosing every 4 weeks for a total of 2 injections to healthy adults.
- To evaluate the safety and tolerability of VRC-HIVMAB060-00-AB (VRC01) administered at 20 mg/kg IV by repeat dosing every 4 weeks for a total of 2 infusions to healthy adults.

3.2 Secondary Objectives

- To evaluate the pharmacokinetics of VRC-HIVMAB080-00-AB (VRC01LS) at each dose level through 24 weeks after the last dose.
- To compare the pharmacokinetics of VRC-HIVMAB080-00-AB (VRC01LS) and VRC-HIVMAB060-00-AB (VRC01) at 5 mg/kg SC and 20 mg/kg IV dose levels through 24 weeks after the last dose.
- To determine whether anti-drug antibody (ADA) to VRC01LS can be detected in recipients of VRC-HIVMAB080-00-AB.

3.3 Exploratory Objectives

- To determine if measurable levels of VRC-HIVMAB080-00-AB (VRC01LS) can be found in rectal and oral secretions of subjects, and in cervical fluid samples for women who receive VRC01LS.
- To determine if measurable levels of VRC-HIVMAB060-00-AB (VRC01) can be found in oral secretions of subjects.
- To evaluate for evidence of functional activity of VRC-HIVMAB080-00-AB (VRC01LS) in samples collected at representative timepoints throughout the study.
- To test subjects for the IgG1 allotypes in order to evaluate allotype-specific effects on the VRC01LS and VRC01 pharmacokinetics.

4. STUDY DESIGN

This is an open-label, dose-escalation study to examine safety, tolerability, dose, and pharmacokinetics of the monoclonal antibody, VRC01LS, in healthy adults. The hypothesis is that the VRC01LS will be safe for administration to healthy adults by the intravenous (IV) and subcutaneous (SC) routes. The secondary hypothesis is that VRC01LS will be detectable in human sera with a definable half-life. With this protocol amendment, Group 7 and Group 8 were added to the protocol Version 2.0 with the goal to evaluate VRC01 and VRC01LS safety and pharmacokinetics in the same study.

The study schema is shown in **Table 4.0**.

Table 4. VRC 606 Study Schema

Group	Subjects	Product	Administration Schedule			
			Day 0	Week 4	Week 12	Week 24
1	3	VRC01LS	5 mg/kg IV			
2	3	VRC01LS	5 mg/kg SC			
3	3	VRC01LS	20 mg/kg IV			
4	5	VRC01LS	40 mg/kg IV			
5	15	VRC01LS	5 mg/kg SC		5 mg/kg SC	5 mg/kg SC
6	10	VRC01LS	20 mg/kg IV		20 mg/kg IV	20 mg/kg IV
7	5	VRC01	5 mg/kg SC	5 mg/kg SC		
8	5	VRC01	20 mg/kg IV	20 mg/kg IV		
*Total	49					

*Enrollment up to a total of 60 subjects is permitted in case there are subjects who do not complete the schedule, if additional PK evaluations are needed, or if an enrollment of additional subjects is necessary for safety evaluations (Section 4.3).

Enrollment will begin in the 5 mg/kg VRC01LS dose groups (Groups 1, 2 and 5). Subjects enrolling into the single dose 5 mg/kg dose groups will be randomized to either Group 1 (5 mg/kg IV) or Group 2 (5 mg/kg SC) in a 1:1 ratio. Only subjects expected to be available for 48 weeks of study participation will be enrolled into Group 5 (5 mg/kg SC by repeat dosing).

For each VRC01LS dose (5 mg/kg, 20 mg/kg and 40 mg/kg), following the first product administration, the study team will wait at least 2 days before administering VRC01LS to a second

subject. Safety review decisions and the status of the enrollment process will be transparent to the PSRT throughout the trial and discussed with the PSRT as part of the weekly safety review process.

Subjects will be randomized into open-label Group 7 and Group 8 at a 1:1 ratio to assess safety and pharmacokinetics of VRC01 at 5 mg/kg SC or at 20 mg/kg IV administered every 4 weeks for a total of 2 administrations per subject.

Safety lab samples will be collected through the study as per Schedule of Evaluations (Appendix III). Subjects will keep a daily diary of solicited systemic symptoms for 3 days after each product administration. Pharmacokinetic (PK) samples will be collected at specified intervals through 24 weeks after the subject's last product administration.

In Groups 2-6, when the subject agrees, the optional oral, rectal and cervical fluid samples will be obtained at specified intervals after each product administration. In Groups 7 and 8, when the subject agrees, the optional oral fluid samples will be obtained at specified intervals.

The study will be conducted by the VRC Clinic at the NIH Clinical Center (NIH CC). The study schedule will not require admission to an inpatient unit or an overnight stay but inpatient administration will be an option.

4.1 STUDY POPULATION

All inclusion and exclusion criteria must be met for eligibility.

4.1.1 Inclusion Criteria

A volunteer must meet all of the following criteria:

1. Able and willing to complete the informed consent process.
2. 18 to 50 years of age.
3. Based on history and examination, must be in general good health without history of any of the conditions listed in the exclusion criteria.
4. Willing to have blood samples collected, stored indefinitely, and used for research purposes.
5. Able to provide proof of identity to the satisfaction of the study clinician completing the enrollment process.
6. Screening laboratory values within 84 days prior to enrollment must meet the following criteria:
    - WBC 2,500-12,000/mm^3.
    - WBC differential either within institutional normal range or accompanied by the Principal Investigator (PI) or designee approval.
    - Platelets = 125,000 – 400,000/mm^3.
    - Hemoglobin within institutional normal range.
    - Creatinine ≤ 1.1 x ULN.
    - ALT ≤1.25 x ULN.
    - Negative for HIV infection by the FDA approved method of detection.

Female-Specific Criteria:

7. If a woman is sexually active with a male partner and has no history of hysterectomy, tubal ligation, or menopause, she agrees to use either a prescription birth control method or barrier

birth control method from the time of study enrollment until the last study visit, or to be monogamous with a partner who has had a vasectomy.

8. Negative β-HCG (human chorionic gonadotropin) pregnancy test (urine or serum) on day of enrollment for women presumed to be of reproductive potential.

4.1.2 Exclusion Criteria

A volunteer will be excluded if one or more of the following conditions apply:

1. Previous receipt of monoclonal antibody whether licensed or investigational.
2. Weight >115 kg.
3. History of a severe allergic reaction with generalized urticaria, angioedema or anaphylaxis within the 2 years prior to enrollment that has a reasonable risk of recurrence.
4. Hypertension that is not well controlled.
5. Woman who is breast-feeding, or planning to become pregnant during the study participation.
6. Receipt of any investigational study agent within 28 days prior to enrollment.
7. Any other chronic or clinically significant medical condition that in the opinion of investigator would jeopardize the safety or rights of the volunteer. Including, but not limited to: diabetes mellitus type I, chronic hepatitis; OR clinically significant forms of: drug or alcohol abuse, asthma, autoimmune disease, psychiatric disorders, heart disease, or cancer.

4.2 Clinical Procedures and Laboratory Assays

Evaluation of safety for this study will include laboratory studies, medical history, and physical assessment by clinicians. The study schedule is provided in Appendix III. Total blood volume drawn from each subject will comply with the NIH Clinical Center Guidelines, which is available on the NIH intranet at the following link: http://cc-internal.cc.nih.gov/policies/PDF/M95-9.pdf.

4.2.1 Screening

Screening for this study will be completed through the Vaccine Research Center's screening protocol, VRC 500 (NIH 11-I-0164). Volunteers will be recruited through Institutional Review Board (IRB)-approved advertising. The evaluations and sample collection that will be included in screening are a medical history, physical exam, any laboratory tests needed to confirm eligibility, and pregnancy test (for females of reproductive potential). Additional assessments of health will be conducted at screening based on clinical judgment. Storage samples of PBMCs, plasma and serum will also be collected. Informed consent documents will be reviewed. Counseling related to potential risks of study agent and pregnancy prevention will be performed. An Assessment of Understanding (AoU) will be completed in association with enrollment into VRC 606. Screening records will be kept to document the reason why an individual was screened but not enrolled into the clinical trial.

4.2.2 Enrollment, Study Days and Visit Numbers

In this study, enrollment is defined as the day of assignment of a study identification number and study group schedule (per Section 6.3.1) in the clinical database. For all groups, a clinician will discuss the timing of the study product administration and PK sample collection before completing

an enrollment to help ensure that the subject can comply with the projected schedule.

Day 0 is defined as the day of first product administration.

For calculating elapsed days, each subsequent calendar date is labeled by the next sequential "Study Day" as shown in the Schedule of Evaluations in Appendix III. Because there may be more than one research sampling timepoint of interest per study day, each sample collection timepoint has its own "Visit Number." For this reason, there may be more than one visit number recorded on the same calendar date.

Medical history and Day 0 evaluations prior to the first study product administration are the baseline for subsequent safety assessments.

The day of first study product administration (Day 0) may occur on the same day as enrollment or up to 6 weeks after the Enrollment Day. If Day 0 does not coincide with enrollment, then the enrollment day may be referred to by a negative number of days (i.e., Day -1 to Day -42). The preferred period between enrollment and infusion is shown in the schedule of evaluations, but in rare cases the period may be increased with approval of the PI.

4.2.3 Product Administration

All study agent administrations will be completed according to the assigned group. For women of childbearing potential, study agent administration may not proceed unless a negative pregnancy test has been obtained within the previous 24 hours. Prior to each administration, temperature, blood pressure, heart rate (pulse) and weight will be collected and a targeted physical examination (based on signs, reported symptoms or interim medical history) may be conducted. In all study groups, the subject will be observed for at least 30 minutes following each product administration. In IV groups, the subject will not be permitted to leave the clinic until the PK samples through 1 hour after administration are collected.

If a subject is assigned to an IV administration group, the IV access will be placed in an arm vein in an aseptic manner. A different site will be used for collection of PK blood samples, however, the same site may be used after flushing the line if another site is not available. VRC01LS and VRC01 will be administered with approximately 100 mL normal saline IV over 15-30 minutes, with a target of about 30 minutes for the initial infusion for each subject. Infusions lasting longer than 30 minutes are allowed.

If assigned to a SC administration group, the administration site(s) to be used will be discussed with the subject and must be assessed as acceptable by the clinician and the subject. The preferred SC administration site is the abdomen, but upper arm or thigh may be used. Given the weight criterion in this study, the maximum volume needed to administer a 5 mg/kg SC dose is not expected to exceed 5.75 mL. The SC dose will be administered by standard needle and syringe SC injection methods with about 2.5 mL per injection site. Up to 3 SC injection sites may be used as judged by the clinician to be the best choice for the subject.

SC administration sites should be at least 2 inches apart. Procedures for product preparation and administration are described in Section 7.0.

4.2.4 Solicited Adverse Events and Clinical Follow-up

Subjects will be given a "Diary Card" to use as a memory aid for solicited adverse events, on which to record temperature and systemic symptoms daily for 3 days after study product administration. Subjects will be trained to use the secure database or complete the paper diary card depending on

their preference. When the 3-day diary card parameters are recorded directly by the subject through a password-protected secure database, the subject's electronic record will be available to clinicians in real time, and will be the source for these data. If concerns arise based on the electronic diary card data, or if a subject uses a paper diary card, clinicians may follow up with additional phone calls during reactogenicity period as needed. The written (paper) diary card may be used as a source document. When neither a written nor electronic diary card is available from the subject, the study clinician will note the source of reactogenicity information recorded in the study database. For this study, solicited adverse events occurring during the 3 days after receipt of study agent will include: unusually tired/feeling unwell, muscles aches, headache, chills, nausea and joint pain. Subjects will also record highest measured temperature daily. The diary cards are reviewed for accuracy and completeness at follow-up visits and reactogenicity is recorded without an attribution assessment. Clinicians will follow and collect resolution information for any reactogenicity symptoms that are not resolved after 3 days.

Clinician assessment of the local IV or SC administrations site will be conducted on day of study agent administration and during the scheduled follow-up timepoints after product administration in all groups. Local reactogenicity parameters will include pain/tenderness, swelling, redness, bruising, and pruritus (itchiness) at the injection site.

Events that may require a clinic visit include rash, urticaria, fever of 38.6˚C (Grade 2) or higher lasting greater than 24 hours or significant impairment in the activities of daily living (such as those consistent with Grade 2 or higher impairment). Additionally, arthralgia or other clinical concerns may prompt a study visit based on the judgment of a study clinician. Clinical laboratory assays and clinical evaluations will assess safety and tolerability at specified intervals after each administration.

4.2.5 Pharmacokinetics Procedures

Pharmacokinetic samples will be collected as close as reasonably possible to the target timepoint. However, actual time of collection is critical for pharmacokinetic analysis and will be recorded for all samples. The pharmacokinetic timepoints are shown in Appendix III.

4.2.6 Mucosal Samples

In order to begin to understand the tissue distribution of the VRC01LS and VRC01, and how long after administration these products may be detectable, this protocol includes exploratory collection of mucosal samples. Mucosal sample collection will be optional and not mandatory. Subjects in Groups 2-6 will be offered the option of participating in the mucosal sampling schedule (oral, rectal and cervical). Subjects in Groups 7 and 8 will be offered only an option for collection of oral mucosal secretions. Samples will be collected using small ophthalmic sponges designed for clinical use.

- Oral swabs may be collected at enrollment and days 7 and 84 after a single VRC01LS administration at 5 mg/kg SC (Group 2), 20 mg/kg IV and 40 mg/kg IV dosages (Groups 3 and 4); **OR** at enrollment and days 84, 168, 196, 280 and 336 when product is administered by repeat dosing (Groups 5 and 6); **OR** at enrollment and days 28, 56, 140, 296 when VRC01 is administered by repeat dosing (Groups 7 and 8).
- Rectal mucosal secretions may be collected at enrollment, days 7 and 84 after a single VRC01LS administration at 5 mg/kg SC (Group 2), 20 mg/kg IV and 40 mg/kg IV dosages (Groups 3 and 4); **OR** at enrollment, days 196 and 280 when VRC01LS is administered by repeat dosing (Groups 5 and 6).

- Cervical mucosal secretions for female subjects who agree to these optional collections will be on the same schedule as the rectal mucosal schedule.

In scheduling women for the study product administration and subsequent mucosal sample collections, consideration will be given to the timing to ensure that the cervical secretions are collected between menstrual periods. If menses occur on a scheduled day of mucosal sample collections, the cervical fluid collection will not be done.

4.2.7 Schedule of Evaluations

Refer to the table in Appendix III for details on the schedule of evaluations and the windows permitted for completion of each visit. Schedule 1 is for the IV dose escalation groups (Groups 1, 3, and 4), Schedule 2 is for Group 2 (5 mg/kg SC). Schedule 3 and Schedule 4 are for the VRC01LS administration by repeat dosing, Group 5 (5 mg/kg SC) and Group 6 (20 mg/kg IV), respectively. Schedule 5 and Schedule 6 are for the VRC01 administration by repeat dosing, Group 7 (5 mg/kg SC) and Group 8 (20 mg/kg IV), respectively. These schedules also include instructions for evaluations of subjects who discontinue product administration. After enrollment, deviations from the visit windows are discouraged and will be recorded as protocol deviations, but will be permitted at the discretion of the PI (or designee).

Additional visits and blood drawing may be scheduled during the study if needed to assess subject safety or for sample collection for immunological testing. After study completion, subjects may be invited to participate in one of the VRC sample collection protocols (VRC 200 or VRC 900) for follow-up sample collection.

Any evaluation for an adverse event or possible exacerbation of a pre-existing condition may be evaluated at study team discretion as a “protocol related” evaluation.

4.2.8 Concomitant Medications

Only routine prescription medications will be entered in the database at the time of enrollment. Subsequently, concomitant medications associated with an adverse event that requires expedited reporting or the development of a new chronic condition requiring ongoing medical management will be recorded. Otherwise, concomitant medications taken throughout the study will be recorded in the subject’s chart as needed for general medical records, but will not be recorded in the study database.

4.3 CRITERIA FOR DOSE ESCALATION

There are two dose escalation reviews in this study. The Protocol Safety Review Team (PSRT, Section 8.9) will conduct an interim safety data review before dose escalation may occur. The PSRT must assess the data as showing no significant safety concerns before proceeding with enrollment of the next dose level.

- The first dose escalation review (from 5 mg/kg to 20 mg/kg of VRC01LS) will occur when at least 3 subjects receiving the 5 mg/kg dose have completed 2 weeks of safety follow up visits. The PSRT review will determine if enrollments in Group 3 and Group 6 (20 mg/kg) may begin.
- The second dose escalation review (from 20 mg/kg to 40 mg/kg of VRC01LS) will occur when at least 3 subjects receiving the 20 mg/kg dose have completed 2 weeks of safety

follow up visits. This review will determine if enrollments in Group 4 (40 mg/kg) may begin.

If a first product administration is not completed or there are discontinuations from the study before there are sufficient data to conduct the dose escalation review for a group, then extra subjects may be enrolled into that group in order to have the requisite data on at least 3 subjects. Additionally, adverse event assessed as related to the study agent at the time of a dose escalation review may be judged by the PSRT to warrant adding additional subjects at a given dose level.

The IRB will be provided with documentation of the safety review process and notification of the dose escalation. Consultation with the IRB and FDA, if needed, as per study pause criteria (Section 4.5) will occur if indicated by the review. One outcome of a dose escalation review may be to recommend evaluation of additional subjects at the current dose level and reassess for safety before proceeding to a higher dose level.

4.4 Discontinuation of Product Administration

Under certain circumstances, a subject may be terminated from participating in study product administrations. Subjects who receive at least one product administration will continue follow-up according to the protocol (Schedule of Evaluations, Appendix III), except that the research sample collections will be discontinued for pregnant women or others in which it is contraindicated. The study team will notify the antiretroviral pregnancy registry (http://www.apregistry.com) of any pregnancies that occur after receiving the study product for any subject still on study. Specific events that will require discontinuing a subject from receiving the study product include:

1. Pregnancy;
2. Grade 3 adverse event assessed as related to the study product (with the exception that self-limited Grade 3 solicited reactogenicity does not require discontinuation of product administration);
3. Grade 4 adverse event assessed as related to the study product;
4. Immediate hypersensitivity reaction associated with the study product;
5. Intercurrent illness that is not expected to resolve prior to the next scheduled study product administration which is assessed by the PI (or designee) to require withdrawal from the product administration;
6. Repeated failure to comply with protocol requirements;
7. Co-enrollment into a study in which other investigational research agents will be administered before the subject has completed the follow-up after the last VRC01LS administration;
8. The IND sponsor or the study PI decide to stop or cancel the study;
9. The IRB, Office for Human Research Protections (OHRP) or the FDA halt the study.

4.5 Protocol Criteria for Pausing the Study and Resuming the Study

Administration of the study agent and new enrollments will be paused by the Principal Investigator (PI) according to the criteria noted below. In the event of a pause, the IND Sponsor Medical Officer (MO) will be promptly notified. Pause criteria are as follows:

One (or more) subject experiences a **Serious Adverse Event** (SAE) that is assessed as related to study agent, or

Two (or more) subjects experience the same **Grade 3 or higher** adverse events (AE) assessed as related to study agent (other than self-limited Grade 3 solicited reactogenicity AEs).

Plan for Review of Pauses and Resuming Rules:

Administration of the study agent and enrollments would resume only if review of the adverse events that caused the pause resulted in a recommendation to permit further study product administrations and study enrollments. The reviews to make this decision will occur as follows:

Pauses for related SAEs: The IND Sponsor, with participation by the PI, will consult with the FDA to conduct the review and make the decision to resume, amend or close the study and notify the IRB accordingly.

Pauses for Grade 3 or higher related AEs: The IND Sponsor MO, in consultation with the PI, will conduct the review and make the decision to resume, amend or close the study for the Grade 3 or higher events that meet the criteria for pausing the study. As part of the pause review, the reviewers will also advise on whether the study needs to be paused again for any subsequent events of the same type. The FDA and the IRB will be notified of Grade 3 or higher pause reviews and the IND sponsor decisions.

5. SAFETY AND ADVERSE EVENT REPORTING

5.1 Adverse Events

An adverse event (AE) is any untoward or unfavorable medical occurrence in a human subject, including any abnormal sign (e.g., abnormal physical exam or laboratory finding), symptom, or disease temporally associated with the use of study treatment, whether or not considered related to the study treatment.

Severity of AEs will be assessed using the Version 2.0 of the *DAIDS Table for Grading the Severity of Adult and Pediatric Adverse Events* [November 2014]. The table is available from: **http://rsc.tech-res.com/clinical-research-sites/safety-reporting/daids-grading-tables**
Additional information can be found in Appendix IV.

Reporting of all AEs will occur during the period from first study agent administration through 56 days after each study agent administration. After this through completion of study participation only SAE and new chronic medical conditions that require ongoing medical management will be recorded as AEs in the study database.

5.2 Serious Adverse Events (SAE)

The term “Serious Adverse Event” (SAE) is defined in 21 CFR 312.32 as follows: “An adverse event or suspected adverse reaction is considered serious if, in the view of either the investigator or the sponsor, it results in any of the following outcomes: Death, a life-threatening adverse event, inpatient hospitalization or prolongation of existing hospitalization, a persistent or significant incapacity or substantial disruption of the ability to conduct normal life functions, or a congenital anomaly/birth defect. Important medical events that may not result in death, be life-threatening, or require hospitalization may be considered serious when, based upon appropriate medical judgment,

they may jeopardize the patient or subject and may require medical or surgical intervention to prevent one of the outcomes listed in this definition. Examples of such medical events include allergic bronchospasm requiring intensive treatment in an emergency room or at home, blood dyscrasias or convulsions that do not result in inpatient hospitalization, or the development of drug dependency or drug abuse."

"Life-threatening" refers to an adverse event that at occurrence represents an immediate risk of death to the subject. An event that may cause death if it occurs in a more severe form is not considered life-threatening. Similarly, a hospital admission for an elective procedure is not considered an SAE. In Section 5.3 the term "Expedited Adverse Event" (EAE) encompasses the events that would be considered an SAE by the 21 CFR 312.32 definition.

5.3 Adverse Event Reporting to the IND Sponsor

5.3.1 Expedited Adverse Event (EAE) Reporting Criteria

Requirements, definitions and methods for expedited reporting of AEs are outlined in Version 2.0 (January 2010) of the *Manual for Expedited Reporting of Adverse Events to DAIDS* (DAIDS EAE Manual), which is available on the Regulatory Support Center (RSC) website at **http://rsc.tech-res.com/clinical-research-sites/safety-reporting/manual** . The SAE Reporting Category will be used for this study.

The internet-based DAIDS Adverse Event Reporting System (DAERS) will be used for expedited AE reporting to DAIDS. In the event of system outages or technical difficulties, expedited AE reports may be submitted via the DAIDS EAE Form. For questions about DAERS, please contact DAIDS-ES at **CRMSSupport@niaid.nih.gov** or from within the DAERS application itself. For questions about expedited AE reporting, please contact the RSC (DAIDSRSCSafetyOffice@tech-res.com).

The study products for which expedited reporting is required is as follows:

- VRC-HIVMBA080-00-AB (VRC01LS MAb)
- VRC-HIVMBA060-00-AB (VRC01 MAb)

The NIAID/DAIDS will report all unexpected SAEs related to the study products observed in this clinical trial to the FDA in accordance with 21 CFR 312.32 (IND Safety Reports).

While the subject is in the study reporting period, as defined in Section 5.1, the SAE Reporting Category will be used.

The EAE Manual should be consulted for further detail. Also ensure that any protocol-specific reporting requirements are met.

An EAE report form will be completed and reported to DAIDS within 3 days of investigator awareness, regardless of relationship to study agent

The SAE criteria are as follows:

- Results in death
- Is life-threatening[1]

- Requires (unplanned) inpatient hospitalization or prolongation of hospitalization[2]
- Results in persistent or significant disabilities/incapacity.
- Is a congenital anomaly/birth defect[3]
- Is an important medical event (may jeopardize the patient or may require intervention to prevent one of the outcomes above)

Footnotes:
[1] "Life-threatening" refers to an event in which the patient was at immediate risk of death at the time of the event. It does NOT refer to an event that hypothetically might have caused death if it were more severe.

[2] Per ICH SAE definition, hospitalization is NOT an adverse event (AE), but is an outcome of the event. DO NOT REPORT: Any admission unrelated to an AE (e.g., for labor/delivery, cosmetic surgery, administrative or social admission for temporary placement for lack of a place to sleep); protocol-specified admission (e.g., for a procedure required by protocol); admission for diagnosis or therapy of a condition that existed before receipt of study agent(s) **and** has not increased in severity or frequency as judged by the clinical investigator. (**NOTE**: A new AIDS-defining event in a subject already known to be HIV-infected would be considered an increase in severity of a pre-existing condition [HIV infection] and would be reportable.)

[3] Clinically insignificant physical findings at births including those regarded as normal variants do NOT meet reporting criteria. If a clinically significant anomaly is reported, all findings (including those of no individual significance) should be included in the same report. For example, do NOT report an isolated finding of polydactyly (extra fingers or toes) or Mongolian spot in an infant. But if either finding occurred with a major cardiac defect, report all findings in the SAE Report.

5.3.2 Attribution Categories

Attribution categories used (i.e. terms used for assessment of relationship of AE to study agent) for this study will be consistent with those described in the DAIDS EAE Manual, Version 2.0 (January 2010), as follows:

- **Related –** There is a reasonable possibility that the AE may be related to the study agent(s).

- **Not Related –** There is not a reasonable possibility that the AE is related to the study agent(s).

If circumstances arise where other attribution categories are used in describing an adverse event, the attribution terms "Definitely", "Probably" and "Possibly" related will be mapped to the "Related" category while the terms "Unlikely," "Probably Not Related" and "Not Related" will be mapped to the "Not Related" category under EAE Manual, Version 2.0.

5.4 REPORTING TO THE INSTITUTIONAL REVIEW BOARD

5.4.1 Unanticipated Problem (UP) Definition

.A serious "Unanticipated Problem (UP)" is defined as any incident, experience, or outcome that meets all three of the following criteria:

- unexpected in nature, severity, or frequency in relation to the research risks that are described in the protocol, informed consent, Investigator's Brochure, other study documents or in consideration of the characteristics of the subject population being studied; **and**
- related to participation in the research; **and**
- suggests that the research places subjects or others at a greater risk of harm (including physical, psychological, economic, or social harm) than was previously known or recognized.

Non-serious UP: An UP that is not an Adverse Event (UPnonAE) is an unanticipated problem that does not fit the definition of an adverse event, but which may, in the opinion of the investigator, involve risk to the subject, affect others in the research study, or significantly impact the integrity of research data. Such events would be considered a non-serious UP. For example, we will report occurrences of breaches of confidentiality, accidental destruction of study records or samples, or unaccounted-for study drug.

5.4.2 Protocol Deviation Definition

A Protocol Deviation is defined as any change, divergence, or departure from the IRB-approved study procedures in a research protocol. Protocol deviations are designated as serious or non-serious and further characterized as

- Those that occur because a member of the research team deviates from the protocol.
- Those that are identified before they occur, but cannot be prevented.
- Those that are discovered after they occur.

Serious Protocol Deviation: A deviation that meets the definition of a SAE or compromises the safety, integrity of the data, welfare or rights of subjects or others.

5.4.3 Non-Compliance Definition

Non-compliance is the failure to comply with applicable NIH Human Research Protections Program (HRPP) policies, IRB requirements, or regulatory requirements for the protection of human subjects. Non-compliance is further characterized as serious, continuing or minor.

"Serious non-compliance" is defined as non-compliance that

- Increases risks, or causes harm, to participants
- Decreases potential benefits to participants
- Compromises the integrity of the NIH-HRPP
- Invalidates the study data

"Continuing non-compliance" is non-compliance that is recurring.

"Minor non-compliance" is non-compliance that is neither serious nor continuing.

5.4.4 Expedited Reporting to the NIAID IRB

The following will be reported within 7 calendar days of investigator awareness:

- Serious and non-serious UP
- Deaths

- Serious protocol deviations
- Serious or continuing non-compliance
- SAEs that are possibly, probably, or definitely related to the research regardless of expectedness

The following waiver applies to reporting anticipated protocol deviations and expected UPnonAEs: Anticipated deviations in the conduct of the protocol will not be reported to the IRB unless they occur at a rate greater than anticipated by the study team. Expected adverse events will not be reported to the IRB unless they occur at a rate greater than that known to occur in healthy adults. If the rate of these events exceeds the rate expected by the study team, the events will be classified and reported as though they are unanticipated problems.

5.4.5 Annual Reporting to the NIAID IRB

The following will be reported to the NIAID IRB in summary at the time of Continuing Review:

- Serious and non-serious UP
- Expected SAEs that are possibly, probably, or definitely related to the research
- SAEs that are not related to the research
- All adverse events, except expected AEs granted a waiver of reporting
- Serious and Non-Serious Protocol Deviations
- Serious, continuing, and minor non-compliance
- Any trends or events which in the opinion of the investigator should be reported

6. STATISTICAL CONSIDERATIONS

6.1 Overview

This is a phase I dose-escalation study of the safety and pharmacokinetics of VRC-HIVMAB080-00-AB (VRC01LS), a human monoclonal antibody with broad HIV-1 neutralizing activity, administered to healthy adults. Groups 7 and 8 were added to the protocol Version 2.0 to evaluate VRC-HIVMAB060-00-AB (VRC01) and VRC-HIVMAB080-00-AB (VRC01LS) safety and pharmacokinetics in the same study.

6.2 Objectives

The primary objective is to evaluate the safety and tolerability of VRC01LS administered at 5 mg/kg IV, 5 mg/kg SC, 20 mg/kg IV and 40 mg/kg IV as a single dose, and administered at 20 mg/kg IV and 5 mg/kg SC by repeat dosing every 12 weeks for a total of 3 product administrations to healthy adults; and to assess VRC01 at 5 mg/kg SC or at 20 mg/kg IV administered every 4 weeks for a total of 2 administrations per subject. The secondary objectives include the pharmacokinetics at each dose level through 24 weeks after the last dose, and testing for the presence of anti-drug antibody against VRC01LS and VRC01. Determination if measurable levels of VRC01LS and VRC01 can be found in mucosal fluids and evaluation for evidence of functional activity of study products in collected samples are exploratory objectives of the study.

6.3 Size and Accrual

Recruitment will target about 49 healthy adults of age 18-50 with 3 subjects in each Group 1-3; 5 subjects in Group 4 (40 mg/kg IV); 15 subjects in Group 5 (5 mg/kg SC); 10 subjects in Group 6

(20 mg/kg IV) with VRC01LS administered by repeat dosing every 12 weeks for a total of 3 infusions; and 5 subjects in each Group 7 (5 mg/kg SC) and Group 8 (20 mg/kg IV) with VRC01 administered every 4 weeks for a total of 2 administrations per subject. The permitted accrual is 60 subjects to allow for additional enrollments in the event that an enrolled subject does not complete the minimum evaluations needed to meet the protocol criteria for the group dose safety or dose escalation evaluation.

6.3.1 Randomization of Treatment Assignments

In this dose-escalation study the subjects are assigned to the dose group that is open to accrual at the time of enrollment, except Groups 1 and 2 (5 mg/kg IV and 5 mg/kg SC, respectively) and Groups 7 and 8 (5 mg/kg SC and 20 mg/kg IV, respectively) that are enrolled simultaneously. To minimize possibility of selection bias, the subjects enrolling while these groups are open to accrual will be randomized to one of the two groups in a 1:1 ratio. While these randomizations are ongoing, neither the study staff nor the subject will know in advance of enrollment whether the subject will be randomized to the IV or SC route, however, both the staff and subject will be informed immediately after the enrollment is completed using the electronic AdvantageEDC system (EMMES Corp, Rockville, MD). The randomization code will be provided by the study statistician.

When a subject is enrolled into a Group but does not begin product administrations, a new eligible subject may be enrolled into the same Group.

If any replacement is needed in case of subject withdrawal, the replacement subject will be assigned to the same treatment as the dropout subject in order to complete the safety dataset as planned.

Dose escalation rules are described in Section 4.3.

6.3.2 Sample Size Considerations

This study is primarily descriptive. For safety analysis, the goal is to identify safety concerns associated with different VRC01LS dosages. There may be as few as 3 to 5 subjects in a group at the time of a dose escalation; therefore, this section considers group sizes of both n=3 and n=5.

The ability to identify serious adverse experiences is best expressed by the maximum true rate of SAE that would unlikely be observed and the minimum true SAE rate that would very likely be observed. Within a group of size n=3, there is a 90% chance of observing at least 1 event if the true rate is no less than 0.536 and a 90% chance of observing no event if the true rate is no bigger than 0.034. Within a group of size n=5, there is a 90% chance of observing at least 1 event if the true rate is no less than 0.37 and a 90% chance of observing no event if the true rate is no bigger than 0.02.

Probabilities of observing 0 or more than 1 event are presented in Table 6-1 for a range of possible true event rates. These calculations provide a complete picture of the sensitivity of this study design to identify potential safety problems with the study agent. For example, within the group of size n=3, if the true event rate is 0.01, then there is a probability of 0.97 to observe no event and a probability of <0.001 to observe more than 1 event; while, within the group of size n=5, if the true event rate is 0.01, then there is a probability of 0.951 to observe no event and a probability of 0.001 to observe more than 1 event.

Table 6-1: Probability of event for different scenarios

True event rate	Within a group (n=3)		Within a group (n=5)	
	Pr(0 event observed)	Pr(more than 1 event observed)	Pr(0 event observed)	Pr(more than 1 event observed)
0.01	0.97	0	0.951	0.001
0.03	0.913	0.003	0.859	0.008
0.05	0.857	0.007	0.774	0.023
0.1	0.729	0.028	0.59	0.081
0.2	0.512	0.104	0.328	0.263
0.3	0.343	0.216	0.168	0.472
0.4	0.216	0.352	0.078	0.663

Tables 6-2 gives the upper and lower bounds for 95% exact binomial confidence intervals for all possible number of observed events within a group. Within the group of size n=3, if no subjects experience the event, the 95% exact 2-sided confidence interval for the true rate has upper bound as 0.708; if all subjects experience the event, the 95% exact 2-sided confidence interval for the true rate has lower bound as 0.292. Within the group of size n=5, if no subjects experience the event, the 95% exact 2-sided confidence interval for the true rate has upper bound as 0.522; if all subjects experience the event, the 95% exact 2-sided confidence interval for the true rate has lower bound as 0.478.

Table 6-2: 95% confidence intervals of the true rate for all possible number of observed events within a group

Within a group (n=3) 95% confidence interval			Within a group (n=5) 95% confidence interval		
Observed rate	Lower bound	Upper bound	Observed rate	Lower bound	Upper Bound
0/3	0	0.708	0/5	0	0.522
1/3	0.008	0.906	1/5	0.005	0.716
2/3	0.094	0.992	2/5	0.053	0.853
3/3	0.292	1	3/5	0.147	0.947
			4/5	0.284	0.995
			5/5	0.478	1

Table 6-1 and 6-2 apply to the secondary and exploratory endpoints as well.

6.4 Statistical Analysis

6.4.1 Analysis Variables

The analysis variables consist of baseline variables, pharmacokinetics and safety variables for primary and secondary objective analyses.

6.4.2 Baseline Demographics

Baseline characteristics including demographics and laboratory measurements will be summarized using descriptive statistics.

6.4.3 Safety Analysis

Summaries of the number and percentage of subjects experiencing any AE or reactogenicity will be tallied by subgroup, and presented along with exact 95% confidence intervals for the proportion.

Solicited Adverse Events:
Solicited adverse event data is collected after each dose administered in this study. The number and percentage of subjects experiencing each type of solicited sign or symptom will be tabulated by severity. For a given sign or symptom, each subject's solicited AEs will be counted once under the maximum severity for all assessments.

Adverse Experiences:
Unsolicited AEs are coded into MedDRA preferred terms. The number and percentages of participants experiencing each specific AE will be tabulated by severity and relationship to treatment. For the calculations in these tables, each participant's adverse experience will be counted once under the maximum severity or strongest recorded causal relationship to treatment.

A complete listing of adverse experiences for each participant will provide details including severity, relationship to treatment type, onset, duration and outcome.

Local laboratory values:
Boxplots of local laboratory values will be generated for baseline values and for values measured during the course of the study. Each boxplot will show the 1st quartile, the median, and the 3rd quartile. Outliers, or values outside the boxplot, will also be plotted. If appropriate, horizontal lines representing boundaries for abnormal values will be plotted.

6.4.4 Tolerability Evaluation

The tolerability of the medical product represents the degree to which overt adverse effects can be tolerated by the subject [17]. VRC 606 is the first trial of VRC01LS in healthy adults. The tolerability evaluation will be mostly descriptive by nature and consist of solicited adverse events that occur during and in the 3 days following each VRC01LS administration and reasons for any withdrawal or discontinuation based upon subject discomfort. This early assessment of tolerability of VRC01LS will inform which parameters should be solicited or routinely assessed to further characterize the tolerability profile in larger number of subjects.

6.4.5 Pharmacokinetics Analysis

Blood samples for PK evaluations will be collected at timepoints defined in the Schedule of Evaluations (Appendix III).

Individual Subject Pharmacokinetic Analysis: A non-compartmental pharmacokinetic analysis will be performed using Phoenix (Centara) or a similar program on the VRC01LS concentration data generated from each subject. Calculated pharmacokinetic parameters for IV Groups 1, 3, 4, and 6 will include: area-under-the-curve (AUC), maximum concentration (Cmax), time to Cmax (Tmax), clearance (CL), volume of distribution (Vdz), terminal elimination rate constant (λz) and the terminal half-life ($T_{1/2}$). For Groups 2 and 5, the PK parameters will include AUC, Cmax, Tmax, apparent clearance (CL/F), apparent volume of distribution (Vdz/F), λz and $T_{1/2}$. Cmax and Tmax will be taken directly from the observed concentration-time data. The terminal slope, λz, will be determined from the log-linear portion of the curve and the $T_{1/2}$ calculated as $0.693/\lambda z$. $AUC_{0\text{-}Clast}$

will be determined using the linear trapezoidal method, where C_{last} is the concentration at 12 weeks after the first two doses in Groups 5-6 and the concentration at 24 weeks after dose in Groups 1, 3 and 4 and final dose in Groups 5 and 6. If the final sample (C_{last}) has measurable VRC01LS concentrations, the remaining AUC after the final concentration ($AUC_{Clast\text{-}inf}$) will be estimated as Clast/λz. Data will be summarized based by each dose Group and overall for IV administration Groups for CL, Vd and $T_{1/2}$. For groups 5 and 6, potential accumulation will be assessed as the ratios of the $AUC_{0\text{-}inf}$ and C_{max} for the first and the last doses. The potential for non-linearity pharmacokinetics among IV Groups will be determined by comparing the dose-adjusted ratios for Cmax and AUC between IV dosing Groups. Additional compartmental analysis will be performed as warranted by the data.

Population Pharmacokinetic Analyses: Population pharmacokinetic analyses will be performed on the VRC01LS pharmacokinetic data following IV and SQ administration to determine compartmental PK parameters with the program NONMEM. One, two and three compartment pharmacokinetic models will be assessed. Based on prior pharmacokinetic studies of antibodies, including VRC01, it is anticipated that a two compartment model will adequately characterize the data. The population analysis will generate estimates for initial and final volumes of distribution (Vd_1 and Vd_2), inter-compartmental clearance (Q), CL and bioavailability (F). Given the small subject numbers, the population PK analysis will not include an exploratory covariate analysis to assess clinical factors as fixed effects associated with VRC01LS PK parameters with the exception of dose level (5 vs 20 vs 40 mg/kg), and first vs subsequent doses as a fixed effects on CL, Vd_1, and Vd_2. The terminal half-life, $t_{1/2\beta}$ will be determined from CL. Vd_1, Vd_2 and Q. Final model selection will be based on changes in the objective function and graphically by goodness of fit plots. The final population model will be assessed using bootstrap analysis and dose normalized visual posterior predictive check. VRC01LS dosing strategies and their ability to achieve and maintain of target VRC01LS concentrations will be performed using the final population pharmacokinetic model and Monte Carlo simulations with at least 5000 replicates.

6.4.6 Interim Analyses

Preliminary analyses of pharmacokinetics may be done once per dose level as the data for each dose level is obtained. This will be used to inform decisions about the dose levels to be administered in studies that may begin while VRC 606 is still in progress.

7. PHARMACY PROCEDURES

The study groups and study agent dosing schedule are shown in Table 4. Refer to the Investigator's Brochure for further information about the investigational study agents.

7.1 STUDY PRODUCTS AND ADMINISTRATION REGIMEN

The study includes an investigational monoclonal antibody products described as follows:

- VRC-HIVMAB080-00-AB (VRC01LS)
- VRC-HIVMAB060-00-AB (VRC01)

VRC01LS and VRC01 vials are filled at a concentration of 100 (± 10) mg/mL. Vials contain a colorless to yellow liquid with no visible particles, which is an isotonic, sterile solution. The formulation buffer is composed of 25 mM sodium citrate, 50 mM sodium chloride, and 150 mM L-

arginine hydrochloride at pH 5.8. Vials are intended for single use only and thus do not contain a preservative.

In calculating the dose to administer and number of vials to thaw, it should be assumed that the concentration is 100 mg/mL and that a volume of at least 6 mL can be withdrawn from a vial. In this trial, dose is limited or established based on subject weight. *For example*, for a subject weighing 115 kg (the upper limit per protocol eligibility) who receives the 40 mg/kg dose, the VRC01LS amount needed is calculated as follows: 115 kg x 40 mg/kg = 4600 mg of VRC01LS, which corresponds to 46 mL of the 100 mg/mL solution. Since each product vial contains at least 6 mL, 8 vials will be needed for the dose preparation for this subject.

Preparation of VRC01LS and VRC01 for IV administration will require a 100 mL bag of 0.9% sodium chloride for injection, USP (normal saline). Note that the normal saline bags referred to as "100 mL bags" in the IV administration instructions will typically have 103 mL volume before any product is added and this is acceptable in the context of the instructions below. Preparation of VRC01LS and VRC01 for SC administration will not require any diluent.

There are 8 different schedules in the study described in Section 4 of the protocol.

7.2 Study Product Storage

The VRC01LS and VRC01 product labels designate the long-term storage temperature as -35°C to -15°C. Clinical site storage in a qualified, continuously monitored, temperature-controlled freezer with a temperature range of -45°C to -10°C is acceptable.

The site pharmacist must promptly report any storage temperature excursions to the IND sponsor's authorized representative (see Appendix II). The affected product must be quarantined in a separate area. The IND Sponsor's authorized representative will notify the site pharmacist if continued clinical use of the product is acceptable.

7.3 Preparation of Study Products for Administration

This section describes how the site pharmacist will prepare the study product for administration and how the clinician will administer the product. Clinician instructions on how to select an administration site are in Section 4.2.3.

Based on results from a stability study, VRC01LS and VRC01 are stable in the vial for at least 8 hours at refrigerated (2°C-8°C) and room temperature (maximum 27°C). The following instructions apply to preparing the product for infusion:

1. Thaw the product vial(s) at room temperature and hold for 30-60 minutes post-thaw (no ice crystals present) prior to use.
2. Keep the material at refrigerated or room temperature during the entire preparation period until use.

Preparation is to be done in a clean preparation unit with limited access using aseptic technique. Assure that only the required vials are present in the preparation unit during dilution, and medication labels are strictly segregated to avoid mix-ups.

If particles are observed in the vialed material at the time of preparation of the IV infusion, the pharmacist must indicate on the bag that an in-line filter infusion set must be used for administration. In-line filters must comply with the following specifications: 1.2 micron PES

(polyethersulfone) filter membrane, DEHP-free, latex-free (equivalent to Braun #473994 filter extension set). Vials with visible particles may be stored as per Section 6.1.3 of the IB and used for product preparation when particles are no longer visible.

After preparation for administration using IV bags or syringes, the product should be administered within 8 hours after removing the vaccine vials from the freezer.

More information on product preparation can be found in the product IBs.

7.3.1 VRC-HIVMAB080-00-AB and VRC-HIVMAB060-00-AB: Preparation for Administration Intravenously

It is expected that each vial will be used for the 6 mL withdrawal volume (600 mg of the product), however, more may be withdrawn if it is possible to do so. For each IV infusion order, the subject's weight and dosage level will be included in the pharmacy order. The IV bag prepared by the pharmacy will include information regarding the total amount (mg) of the product added to the 103 mL normal saline bag, and the final volume of the bag. Prior to IV administration, the nurse responsible for administration and another clinician will each check the bag label and confirm that the identifier is correct and that the correct total mg to be administered is shown based on subject weight and dosage level before beginning the IV administration.

To prepare an IV infusion, the pharmacist will calculate the total mg needed, thaw the minimum number of vials needed to obtain the full dose and add the calculated total mg needed to a 103 mL bag of normal saline using good pharmacy practices to maintain sterility. After thawing, the vials should be gently swirled for 30 seconds to avoid foaming. DO NOT SHAKE THE VIAL. The bag of normal saline has the capacity to accept up to 50 mL of added product and this will be sufficient to accommodate all the planned dose levels for the eligible subjects in this study. Any unused portion of a product vial will not be used for another subject.

The study product solution will typically be administered IV over about 15-30 minutes using a volumetric pump. Therefore, for a 15 to 30 minute administration, the rate of infusion may range from 10-20 mg/kg/hr with the lowest dose group to 80-160 mg/kg/hr with the highest dose group. The mL/hr infusion rate may vary based on the total volume needed to administer the full dose. The total time needed to administer the dose may be longer than 30 minutes, based on factors such as subject tolerance.

7.3.2 VRC-HIVMAB080-00-AB and VRC-HIVMAB060-00-AB: Preparation for Administration Subcutaneously

It is expected that each vial will be used for the 6 mL withdrawal volume (600 mg of the product), however, more may be withdrawn if it is possible to do so. For each SC administration order, the subject's weight and dosage level (5mg/kg SC) will be included in the pharmacy order. To prepare the SC administration order, the pharmacist will calculate the total mg needed and thaw the minimum number of vials needed to obtain the full dose. After thawing, the vials should be gently swirled for 30 seconds to avoid foaming. DO NOT SHAKE THE VIAL. The needed volume will be loaded into 1 to 3 syringes for SC needle and syringe direct injection as indicated by the order submitted to the pharmacy.

When administered by a direct SC injection with needle and syringe, the clinician will use proper SC technique to ensure administration into the SC fatty layer and a slow push to minimize discomfort or the excessive distention of overlying skin.

7.4 Labeling of Study Agents

Vials of study agents will be individually labeled with the name of the material, volume, lot number, concentration, storage instructions, Investigational Use Statement ("Limited by Federal Law to Investigational Use"), and manufacturer information.

7.5 Study Agent Accountability

The study pharmacist will be responsible for maintaining an accurate record of the codes, inventory, and an accountability record of study agent supplies. Electronic documentation as well as paper copies may be used.

7.6 Study Agent Disposition

The empty vials and the unused portion of a vial will be discarded in a biohazard containment bag and incinerated or autoclaved. Any unopened vials that remain at the end of the study will be returned to the production facility or discarded at the discretion of the sponsor in accordance with policies that apply to investigational agents. Partially used vials will not be administered to other subjects or used for *in vitro* experimental studies. These vials will be disposed of in accordance with institutional or pharmacy policy.

8. HUMAN SUBJECT PROTECTIONS AND ETHICAL OBLIGATIONS

This research study will be conducted in compliance with the protocol, Good Clinical Practices (GCP), and all applicable regulatory requirements.

8.1 Informed Consent

The study informed consent is provided in Appendix I. It describes the investigational product to be used and all aspects involved in protocol participation.

Before a subject's participation in the study, it is the investigator's responsibility to obtain written informed consent from the subject, after adequate explanation of the aims, methods, anticipated benefits, and potential hazards of the study and before any protocol-specific procedures are conducted or study agent is administered. The Assessment of Understanding quiz will be completed before the study consent is signed.

The acquisition of informed consent will be documented in the subject's medical records, as required by 21 CFR 312.62. The informed consent form will be signed and personally dated by the subject and the person who conducted the informed consent discussion. The original signed informed consent form will be retained in the medical chart and a copy will be provided to the subject.

8.2 Risks and Benefits

8.2.1 Risks

VRC-HIVMAB060-00-AB (VRC01): First human clinical trials of VRC-HIVMAB060-00-AB MAb determined that VRC01 is safe for further evaluation in HIV-infected and healthy adults. The solicited local and systemic signs and symptoms following administration of VRC01 were generally none to mild. The SC administrations were sometimes associated with mild local reactions (Section 1.4.2).

VRC-HIVMAB080-00-AB: Experience with the VRC01LS administration to the first 26 subjects enrolled in VRC 606 study suggests that VRC01LS has an acceptable safety profile with the solicited local and systemic reactogenicity symptoms being predominantly none to mild. The SC administrations were associated with pain/stinging sensation during VRC01LS injection that resolved within 2-5 minutes after injection (Section 1.4.1).

Administration of MAbs may cause immune reactions such as acute anaphylaxis, serum sickness and the generation of antibodies. However, these reactions are rare and more often associated with MAb targeted to human proteins or with the use of murine monoclonal antibodies which would have a risk of human anti-mouse antibodies [18]. In this regard, as VRC01 and VRC01LS are targeted to a viral antigen and are human monoclonal antibodies; these are expected to have a low risk of such side effects.

Typically, the side effects of MAbs are mild but may include fever, chills, rigors, nausea, vomiting, pain, headache, dizziness, shortness of breath, bronchospasm, hypotension, hypertension, pruritus, rash, urticaria, angioedema, diarrhea, tachycardia or chest pain. Clinical use of MAbs that are targeted to cytokines or antigens associated with human cells may be associated with an increased risk of infections [18]; however, this is not expected to be a risk for a MAb targeted to a viral antigen.

It is known from published experience with human MAb directed against the cell surface targets on lymphocytes, that infusion of a MAb may be associated with cytokine release, causing a reaction known as “cytokine release syndrome” (CRS) [19]. Most infusion-related events occur within the first 24 hours after beginning administration. Severe reactions, such as anaphylaxis, angioedema, bronchospasm, hypotension and hypoxia, are infrequent and more often associated with MAbs targeted to human proteins or when a non-human MAb, such as a murine MAb, is used [18]. Specifically, with regard to CRS reactions, these most commonly occur within the first few hours of beginning the infusion and are more common with the first MAb infusion received. This is because the cytokine release is associated with lysis of the cells targeted by the MAb and the burden of target cells is greatest at the time of the first MAb treatment. With licensed therapeutic MAbs, CRS is managed by temporarily stopping the infusion, administration of histamine blockers and restarting the infusion at a slower rate [20].

Delayed allergic reactions to other MAbs may include a serum sickness type of reaction, which is characterized by urticaria, fever, lymph node enlargement, and joint pains. These symptoms may not appear until several days after the exposure to the MAb and is noted to be more common with chimeric types of MAbs [18].

There are several FDA-licensed MAbs for which reactions related to the rate of IV infusion have been described. Some symptoms may be treated by slowing or stopping the infusion. Supportive treatment may also be indicated for some signs and symptoms.

VRC01 did not cause a positive test result in standard antibody-based HIV-1/2 diagnostic tests performed on HIV-uninfected plasma samples spiked with VRC01 to achieve concentrations of 200, 50 and 1 mcg/mL. No positive HIV-1/2 diagnostic tests have been observed in HIV-uninfected clinical trial participants to date. VRC01LS is not expected to cause positive test results on standard diagnostic tests.

Participation in this study may limit a subject’s eligibility for other future MAb studies.

Risks of Blood Drawing: Blood drawing may cause pain and bruising and may, infrequently, cause a feeling of lightheadedness or fainting. Rarely, it may cause infection at the site where the blood is taken. In this study, an IV line that can be used for the collection of blood may be placed and left in place for several hours on the days when there are frequent PK blood draws. Problems from use of an IV for blood drawing are generally mild and may include pain, bruising, minor swelling or bleeding at the IV site and rarely, infection, vein irritation (called phlebitis), or blood clot.

Risks of Mucosal Sample Collection: Collection of samples by swabs and wicks by rubbing them over the mucosal surfaces can cause momentary discomfort and, in some cases, minor bleeding.

8.2.2 Benefits

There are no direct benefits to study subjects from study participation. Others may benefit from knowledge gained in this study that may aid in the development of HIV prevention or therapeutic methods.

8.3 INSTITUTIONAL REVIEW BOARD

A copy of the protocol, informed consent form, other written subject information, and any advertising material will be submitted to the IRB for written approval.

The investigator must submit and, where necessary, obtain approval from the IRB for all subsequent protocol amendments and changes to the informed consent document. The investigator will notify the IRB of unanticipated problems, non-compliance, deviations from the protocol, and serious SAEs as described in Section 5.4.

The investigator will be responsible for obtaining IRB approval of the annual Continuing Review throughout the duration of the study.

8.4 PROTOCOL REGISTRATION

Prior to implementation of this protocol, and any subsequent full version amendments, the study site must have the protocol and the inform consent form (ICF) approved, as appropriate, by the site IRB/ethics committee (EC) and any other applicable regulatory entity (RE). Upon receiving final approval, the site will submit all required protocol registration documents to the DAIDS Protocol Registration Office (DAIDS PRO) at the Regulatory Support Center (RSC). The DAIDS PRO will review the submitted protocol registration packet to ensure that all of the required documents have been received.

The ICF will be reviewed and approved by the DAIDS PRO and the site will receive an Initial Registration Notification from the DAIDS PRO that indicates successful completion of the protocol registration process. A copy of the Initial Registration Notification should be retained in the site's regulatory files.

Upon receiving final IRB/EC and any other applicable RE approval(s) for an amendment, the site should implement the amendment immediately. The study site is required to submit an amendment registration packet to the DAIDS PRO at the RSC. The DAIDS PRO will review the submitted protocol registration packet to ensure that all the required documents have been received. The ICF will not be reviewed and approved by the DAIDS PRO, and sites will receive an Amendment Registration Notification when the DAIDS PRO receives a complete registration packet. A copy of the Amendment Registration Notification should be retained in the site's regulatory files.

For additional information on the protocol registration process and specific documents required for initial and amendment registrations, refer to the current version of the DAIDS Protocol Registration Manual.

8.5 Subject Confidentiality

The investigator must ensure that no information identifying the subject will be released to any unauthorized party. Individual identifying information will not be included in any reports. Subjects will be identified only by coded numbers. All records will be kept confidential to the extent provided by federal, state and local law. Medical records are made available for review when required by the FDA or other authorized users, such as the study agent manufacturer, only under the guidelines set by the Federal Privacy Act. Direct access includes examining, analyzing, verifying, and reproducing any records and reports that are important to the evaluation of the study. The investigator is obligated to inform the subjects that the above named representatives will review their study-related records without violating the confidentiality of the subjects.

8.6 Plan for Use and Storage of Biological Samples

The plan for use and storage of biological samples from this protocol is as outlined in the following sections.

8.6.1 Use of Samples, Specimens and Data

Samples, specimens and data collected under this protocol may be used to conduct protocol-related safety and immune response evaluations, exploratory laboratory evaluations related to the type of infection the study agent was designed to prevent, exploratory laboratory evaluations related to vaccine or infectious disease research in general and for research assay validation. Genetic testing may be performed in accordance with the genetic testing information that was included in the study informed consent.

8.6.2 Storage and Tracking of Blood Samples and Other Specimens

All of the stored study research samples are labeled by a code (such as a number) that only the VRC Clinic can link to the subject. Samples are stored at the NIAID Vaccine Immune T-Cell and Antibody Laboratory (NVITAL), Gaithersburg, MD or VRC Laboratories in Building 40, which are both secure facilities with limited access. Data will be kept in password-protected computers. Only investigators or their designees will have access to the samples and data. Samples will be tracked in the Laboratory Information Management System (LIMS) database or using another software designed for this purpose (e.g., Freezerworks).

8.6.3 Disposition of Samples, Specimens and Data at Completion of the Protocol

In the future, other investigators (both at NIH and outside) may wish to study these samples and/or data. IRB approval must be sought prior to any sharing of samples. Any clinical information shared about those samples would similarly require prior IRB approval. The research use of stored, unlinked or unidentified samples may be exempt from the need for prospective IRB review and approval. Exemption requests will be submitted in writing to the NIH Office of Human Subjects Research, which is authorized to determine whether a research activity is exempt.

At the time of protocol termination, samples will remain in the NVITAL facility or VRC laboratories or, after IRB approval, transferred to another repository. Regulatory oversight of the stored samples and data may be transferred to a stored samples protocol as part of the IRB-approved

termination plan. Data will be archived by the VRC in compliance with requirements for retention of research records, or after IRB and study sponsor approval, it may be either destroyed or transferred to another repository.

8.6.4 Loss or Destruction of Samples, Specimens or Data

The NIH Intramural Protocol Deviation definition related to loss of or destruction of samples or data will be followed. Any loss or unanticipated destruction of samples (for example, due to freezer malfunction) or data (for example, misplacing a printout of data with identifiers) that compromises the scientific integrity of the study will be reported to the IRB in accordance with institutional policies. The PI will also notify the IRB if the decision is made to destroy the remaining samples.

8.7 Subject Identification and Enrollment of Study Participants

All study activities will be carried out at the NIH CC. Study subjects will be recruited through on-site and off-site advertising done for the screening protocol, VRC 500 (NCT 01375530) (https://clinicaltrials.gov/ct2/show/NCT01375530?term=VRC+500&rank=1). Effort will be made to include women and minorities in proportions similar to that of the community from which they are recruited and will be limited to persons at least 18 years of age and no older than 50 years of age at enrollment.

8.7.1 Participation of Children

Children are not eligible to participate in this clinical trial because the study agent has not been previously evaluated in adults. If the product is assessed as safe for further study other protocols specifically designed for children may be conducted.

8.7.2 Participation of NIH Employees

NIH employees and members of their immediate families may participate in this protocol. We will follow the Guidelines for the Inclusion of Employees in NIH Research Studies and will give each employee a copy of the “NIH Information Sheet on Employee Research Participation” and a copy of the “Leave Policy for NIH Employees Participating in NIH Medical Research Studies.” Neither participation nor refusal to participate will have an effect, either beneficial or adverse, on the participant’s employment or work situation. The NIH information sheet regarding NIH employee research participation will be distributed to all potential subjects who are NIH employees. The employee subject’s privacy and confidentiality will be preserved in accordance with NIH Clinical Center and NIAID policies. For NIH employee subjects, consent will be obtained by an individual who is independent of the employee’s team. If the individual obtaining consent is a co-worker to the subject, independent monitoring of the consent process will be included through the Bioethics Consultation Service. Protocol study staff will be trained on obtaining potentially sensitive and private information from co-workers or subordinates.

8.8 Compensation

Subjects will be compensated for time and inconvenience in accordance with the standards for compensation of the Clinical Research Volunteer Program. The compensation will be $175 for outpatient scheduled visits that include oral swabs, and blood drawing. Study agent administration accompanied by the same day PK blood draws combined will be $325. Study agent administration visits without PK blood draws will be $275. The additional compensation when rectal and/or genital secretion collections are included in a visit will be $150. The compensation is $75 for clinic visits

that do not include a blood draw or procedure, and $25 for completion of electronic diary card.

8.9 Safety Monitoring

Close cooperation between the designated members of the Protocol Team will occur to evaluate and respond to individual AEs in a timely manner. The VRC designated Safety Officer for the day conducts a daily safety review of clinical data per VRC Standard Operating Procedures. The PSRT, comprised of the PI, Associate Investigators, Study Coordinator, Protocol Specialists, other Study Clinicians, and DAIDS Medical Officer will review the summary study safety data reports on a weekly basis through 4 weeks after the last subject receives the last product administration in order to be certain that the study agent has an acceptable safety profile and will continue to monitor the study safety data reports on a monthly basis through completion of the last study visit.

9. ADMINISTRATIVE AND LEGAL OBLIGATIONS

9.1 Protocol Amendments and Study Termination

Protocol Amendments must be made only with the prior approval of the DAIDS/NIAID and VRC. Agreement from the PI and DAIDS Medical Officer (MO) must be obtained for all protocol amendments and amendments to the informed consent document. All study amendments will be submitted to the IRB for approval.

The DAIDS, VRC, the NIAID IRB, the Office of Human Research Protections, the study PI, and FDA reserve the right to terminate the study. The PI will notify the IRB in writing of the study's completion or early termination.

9.2 Study Documentation and Storage

The PI will maintain a list of appropriately qualified persons to whom trial duties have been delegated.

Source documents are original documents, data, and records from which the subject's data are obtained. These include but are not limited to hospital records, clinical and office charts, laboratory and pharmacy records, microfiches, radiographs, and correspondence.

The PI and staff are responsible for maintaining a comprehensive and centralized filing system of all study-related (essential) documentation, suitable for inspection at any time by representatives from the DAIDS/NIAID and VRC, IRB, FDA, and/or applicable regulatory authorities. Elements include:

- Subject files containing completed informed consent forms, and supporting copies of source documentation (if kept)
- Study files containing the protocol with all amendments, Investigator Brochures, copies of all correspondence with the IRB and the NIAID's Division of AIDS and Vaccine Research Center

In addition, all original source documentation must be maintained and be readily available.

All essential documentation should be retained by the institution for the same period of time required for medical records retention. The FDA requires study records to be retained for up to two years after marketing approval or refusal (21 CFR 312.62). No study document should be destroyed

without prior written agreement between the NIAID's Division of AIDS, the Vaccine Research Center, and the investigator. Should the investigator wish to assign the study records to another party or move them to another location, they must notify the DAIDS/NIAID and VRC in writing of the new responsible person and/or the new location.

9.3 Study Monitoring, Data Collection and Data Monitoring

9.3.1 Study Monitoring

The DAIDS/NIAID and VRC regulatory authority inspectors or their authorized representatives are responsible for contacting and visiting the investigator for the purpose of inspecting the facilities and, upon request, inspecting the various records of the trial, provided that subject confidentiality is respected.

Site visits by study monitors will be made in accordance with the IND Sponsor (DAIDS) policy to monitor the following: study operations, the quality of data collected in the research records, the accuracy and timeliness of data entered in the database, and to determine that all process and regulatory requirements are met.

Site investigators will allow the study monitors, the NIAID IRB, and FDA to inspect study documents (e.g., consent forms, drug distribution forms, case report forms) and pertinent hospital or clinic records for confirmation of the study data.

9.3.2 Data Collection

Clinical research data will be collected in a secure electronic data management system through a contract research organization, EMMES (Rockville, MD). Extracted data without patient identifiers will be sent to the PSRT for safety review and to Protocol Statistician for statistical analysis.

9.4 Language

All written information and other material to be used by subjects and investigative staff must use vocabulary and language that are clearly understood.

9.5 Policy Regarding Research-Related Injuries

The NIH Clinical Center will provide short-term medical care for any injury resulting from participation in this research. In general, the National Institutes of Health, the Clinical Center, or the U.S. Federal Government will provide no long-term medical care or financial compensation for research-related injuries.

10. REFERENCES

1. UNAIDS. *Fact sheet: 2014 statistics*. 2014; Available from: http://www.unaids.org/sites/default/files/media_asset/20150714_FS_MDG6_Report_en.pdf.
2. Wu, X., et al., *Rational design of envelope identifies broadly neutralizing human monoclonal antibodies to HIV-1.* Science, 2010. **329**(5993): p. 856-61.
3. Rudicell, R.S., et al., *Enhanced Potency of a Broadly Neutralizing HIV-1 Antibody In Vitro Improves Protection against Lentiviral Infection In Vivo.* J Virol, 2014. **88**(21): p. 12669-82.
4. Kwon, Y., et al., *Structure-guided modification and optimization of antibody VRC07.* Retrovirology, 2012. **9**(Suppl 2): p. O34.
5. Ko, S.Y., et al., *Enhanced neonatal Fc receptor function improves protection against primate SHIV infection.* Nature, 2014. **514**(7524): p. 642-5.
6. Wu, X., et al., *Selection pressure on HIV-1 envelope by broadly neutralizing antibodies to the conserved CD4-binding site.* J Virol, 2012. **86**(10): p. 5844-56.
7. Zalevsky, J., et al., *Enhanced antibody half-life improves in vivo activity.* Nat Biotechnol, 2010. **28**(2): p. 157-9.
8. Ledgerwood, J.E., et al., *Safety, pharmacokinetics and neutralization of the broadly neutralizing HIV-1 human monoclonal antibody VRC01 in healthy adults.* Clin Exp Immunol, 2015.
9. Montefeori, D.C., *Measuring HIV Neutralization in a Luciferase Reporter Gene Assay*, in *HIV Protocols, Second Edition, Vol. 485*, V.R. Prasad, and Kalpana, G.V., Editor. 2009, Humana Press: New York, NY. p. 395-405.
10. Ozaki, D.A., et al., *International technology transfer of a GCLP-compliant HIV-1 neutralizing antibody assay for human clinical trials.* PLoS One, 2012. **7**(1): p. e30963.
11. Todd, C.A., et al., *Development and implementation of an international proficiency testing program for a neutralizing antibody assay for HIV-1 in TZM-bl cells.* J Immunol Methods, 2012. **375**(1-2): p. 57-67.
12. Allen, J.C. and H.G. Kunkel, *Antibodies to genetic types of gamma globulin after multiple transfusions.* Science, 1963. **139**(3553): p. 418-9.
13. Jefferis, R. and M.P. Lefranc, *Human immunoglobulin allotypes: possible implications for immunogenicity.* MAbs, 2009. **1**(4): p. 332-8.
14. Kickler, T.S., et al., *The expression of IgG allotypes on platelets and immunization to IgG allotypes in multitransfused thrombocytopenic patients.* Blood, 1990. **76**(4): p. 849-52.
15. Lynch, R.M., et al., *Virologic effects of broadly neutralizing antibody VRC01 administration during chronic HIV-1 infection.* Sci Transl Med, 2015. **7**(319): p. 319ra206.
16. DHHS. *Panel On Antiretroviral Guidelines for Adults and Adolescents. Guidelines for the use of antiretroviral agents in HIV-1-infected adults and adolescents.* January 28, 2016; Available from: https://aidsinfo.nih.gov/contentfiles/lvguidelines/adultandadolescentgl.pdf.
17. FDA, *Guidance for Industry: E9 Statistical Principles for Clinical Trials*. 1998, U. S. DHHS, FDA, CDER, CBER.
18. Hansel, T.T., et al., *The safety and side effects of monoclonal antibodies.* Nature reviews. Drug discovery, 2010. **9**(4): p. 325-38.
19. Bugelski, P.J., et al., *Monoclonal antibody-induced cytokine-release syndrome.* Expert review of clinical immunology, 2009. **5**(5): p. 499-521.
20. Vogel, W.H., *Infusion reactions: diagnosis, assessment, and management.* Clin J Oncol Nurs, 2010. **14**(2): p. E10-21.

APPENDIX I:
STUDY INFORMED CONSENT FORM
Text to be included in the NIH Clinical Center Informed Consent Template

TITLE: VRC 606: A Phase 1, Dose-Escalation Study of the Safety and Pharmacokinetics of a Human Monoclonal Antibody, VRC-HIVMAB080-00-AB (VRC01LS), and VRC-HIVMAB060-00-AB (VRC01), Administered Intravenously or Subcutaneously to Healthy Adults.

INTRODUCTION

We invite you to take part in a research study at the National Institutes of Health (NIH). First, we want you to know that:

> Taking part in NIH research is entirely voluntary.
>
> You may choose not to take part, or you may withdraw from the study at any time. In either case, you will not lose any benefits to which you are otherwise entitled. However, to receive care at the NIH, you must be taking part in a study or be under evaluation for study participation.
>
> You may receive no benefit from taking part. The research may give us knowledge that may help people in the future.

Second, some people have personal, religious or ethical beliefs that may limit the kinds of medical or research treatments they would want to receive (such as blood transfusions). If you have such beliefs, please discuss them with your NIH doctors or research team before you agree to the study.

Now we will describe this research study. Before you decide to take part, please take as much time as you need to ask any questions and discuss this study with anyone at NIH, or with family, friends or your personal physician or other health professional.

PURPOSE AND PLAN OF THE STUDY

This is the study of two experimental products called “VRC01LS” and “VRC01”. The U.S. Food and Drug Administration (FDA) allows these products to be used for research only. VRC01LS and VRC01 are human antibodies directed against HIV virus. The human body uses antibodies as one way to help fight infection. The main purpose of this study is to see if the experimental products VRC01LS and VRC01 are safe and well-tolerated. We will study the amount of VRC01LS and VRC01 in the body and how it changes over time. We will check to see if people who get VRC01LS or VRC01 develop immune response (antibody) to these products.

About 49 to 60 people will participate in this study at the NIH Clinical Center in Bethesda, Maryland. The study will have about 13-26 clinic visits over 24-48 weeks for each person, depending on the study group.

STUDY PRODUCTS

VRC01 is a monoclonal antibody (“MAb”). “Monoclonal” means that all the antibodies in the product are the same. The formal name for the product is “VRC-HIVMAB060-00-AB.” As of July 27, 2016, about 350 adults and 21 HIV-uninfected infants have received one or more doses

of VRC01 by IV or SC routes. VRC01 was found to be well tolerated and safe for further evaluation.

VRC01LS is also a MAb. The formal name for the product is “VRC-HIVMAB080-00-AB.” VRC01LS is a new version of the monoclonal antibody VRC01 that is identical to VRC01 except for a small structural change. The purpose of this change is to make VRC01LS last longer in the body.

VRC01 and VRC01LS are human antibodies and synthetic products based on an antibody that was first found in an HIV-infected person. Although the antibody was found in a human, the VRC01 and VRC01LS products are not made by collecting these from a human. VRC01 and VRC01LS were developed by the Vaccine Research Center (VRC), NIH and made in a drug manufacturing laboratory. This is the first study to give VRC01LS to humans.

VRC01 and VRC01LS will not protect participants from HIV infection. There is currently no cure for or vaccine to prevent HIV. You cannot get HIV from VRC01 or VRC01LS.

In laboratory and animal studies, VRC01 and VRC01LS were shown to attach to and inactivate many types of HIV viruses. It is not known if these products will act the same way when given to humans. It will take many studies to learn if the product will be useful for preventing or treating HIV. This study alone will not answer this question.

ELIGIBILITY

You are eligible to participate in this study because you have completed the screening process and are known to be the following:

- 18 to 50 years old
- In general good health without significant medical problems as determined at screening
- Willing to receive VRC01LS or VRC01 monoclonal antibodies
- Willing to donate blood samples for future research
- Willing to be tested for HIV infection
- And if female and able to become pregnant: willing to use birth control for the duration of the study.

STUDY PROCEDURES

The study will have 8 groups. Each group will have about 3-15 people in it. The groups are defined by the product administered, the dose of the product and how it is given. Most participants will get the product by the intravenous (IV) route, meaning into a vein. Some participants will get the product by the subcutaneous (SC) route, meaning into the fatty tissue under the skin. Both ways will use a needle. The product administration visit(s) may last about 4-8 hours. Other clinic visits, including mucosal sampling during the study, will take about 2 hours. Study participation will last about 24 - 48 weeks depending on the study group.

If you agree to take part in this study, you will get 1 to 3 doses of the product, depending on the group. You will know how many doses you will get. Each person gets an amount of study product based on their own body weight. We will measure your weight on the day the study product is given to calculate the dose.

The Study Groups are shown in the following table:

<table>
<tr><th colspan="8">VRC 606 Study Schema</th></tr>
<tr><th colspan="2" rowspan="2">Group</th><th rowspan="2">Product</th><th rowspan="2">Subjects</th><th colspan="4">Schedule</th></tr>
<tr><th>Day 0</th><th>Week 4</th><th>Week 12</th><th>Week 24</th></tr>
<tr><td rowspan="2">Randomized</td><td>1</td><td>VRC01LS</td><td>3</td><td>5 mg/kg IV</td><td></td><td></td><td></td></tr>
<tr><td>2</td><td>VRC01LS</td><td>3</td><td>5 mg/kg SC</td><td></td><td></td><td></td></tr>
<tr><td colspan="2">3</td><td>VRC01LS</td><td>3</td><td>20 mg/kg IV</td><td></td><td></td><td></td></tr>
<tr><td colspan="2">4</td><td>VRC01LS</td><td>5</td><td>40 mg/kg IV</td><td></td><td></td><td></td></tr>
<tr><td colspan="2">5</td><td>VRC01LS</td><td>15</td><td>5 mg/kg SC</td><td></td><td>5 mg/kg SC</td><td>5 mg/kg SC</td></tr>
<tr><td colspan="2">6</td><td>VRC01LS</td><td>10</td><td>20 mg/kg IV</td><td></td><td>20 mg/kg IV</td><td>20 mg/kg IV</td></tr>
<tr><td rowspan="2">Randomized</td><td>7</td><td>VRC01</td><td>5</td><td>5 mg/kg SC</td><td>5 mg/kg SC</td><td></td><td></td></tr>
<tr><td>8</td><td>VRC01</td><td>5</td><td>20 mg/kg IV</td><td>20 mg/kg IV</td><td></td><td></td></tr>
<tr><td colspan="3">Total</td><td>49</td><td colspan="4">More people may be enrolled, if needed, to reach the study goals.</td></tr>
</table>

The study will start by assigning participants to get the lowest dose of VRC01LS in Groups 1, 2 or 5. Groups 1 and 2 will be assigned randomly (like flipping a coin). Those who are available for 48 weeks of study participation may enroll into Group 5. If participants in the lower dose groups tolerate VRC01LS, the next dose group may enroll. This pattern will continue until we reach the highest VRC01LS dose. Participants in Groups 7 and 8 will receive VRC01 and will also be assigned randomly (like flipping a coin).

- <u>IV doses</u>: If you are in a group getting the product by IV infusion, we may place a thin tube or IV line in a vein on your arm on the day you get the study product. The product will be mixed into a bag of liquid called “normal saline” or “salt water.” The mix of normal saline and the product will be given directly into your vein. A pump will control how fast the study product goes into the vein. The goal is to give it over about 15-30 minutes. If you have side effects, the rate of the infusion may be slowed down or stopped. At the end of the infusion(s) we will monitor you for 30 minutes and collect blood samples.
- <u>SC doses</u>: If you are in a group getting the product by SC injection, we will put a small needle into the fatty tissue under your skin. The abdominal area is usually where the needle will be inserted in your body. It is possible we may use your arm or thigh area instead. The product will not be mixed with any other liquid. You will get the SC doses by injection with a standard needle and syringe in 1-3 SC sites on your body. At the end of the injection(s) we will monitor you for 30 minutes.

If you are female and able to become pregnant, a pregnancy test will be done before each product administration and the result of the test must be negative to continue.

For IV doses, we will collect blood samples from you before the infusion, at the end of the infusion and about one hour, 3 hours, and 6 hours after you get the product for the first time. We will collect blood samples from you before the infusion, at the end of the infusion, and about one hour after you get the product at the next product infusions. For SC doses, no blood collection is required after the injection on the day of product administration. After you get the study product, you will need to come back to the clinic again 1 to 3 times during that first week for sample

collection, depending on the group. Groups 5 and 6 will come back to the clinic 4 to 5 times between the second and third dose, and follow-up visits will continue to week 48. If you are getting only one dose (Groups 1-4), follow-up visits will continue to week 24. Groups 7 and 8 will come back to the clinic 5-6 times between the first and the second infusions, and follow-up visits will continue to Week 28.

For 3 days after you receive the study product, we will ask you to check your temperature with a thermometer we give you and write it down. We will ask you to write down any symptoms you may have. You will receive a password to a secure website to record this information on an electronic form or “diary.” If you prefer, you will have the option to use a paper diary instead. If you have any side effects, you should tell a study physician or a nurse as soon as possible. You can reach the clinic staff by phone 24 hours a day. If you have symptoms, you may be asked to come into the clinic for an examination before your next scheduled visit. It is very important that you follow the instructions you get from the clinic staff.

At each visit, we will check you for any health changes or problems. We will ask you how you are feeling and if you have taken any medications. We will draw your blood at scheduled study visits and we may also ask for other types of bodily samples (explained below). We will tell you right away if any of your test results show a health problem. We will use some blood samples to study if your body develops immune response (antibody) to the study product. Results of these tests are not for checking on your health and we will not give you these results during the study. We may ask you to come into clinic for additional blood collection. After the study completion, we may invite you to participate in another study for follow-up sample collection.

Experimental studies follow a set schedule. This helps us answer the research questions. Scheduling for your visits allows some flexibility, but it is important that you work with the staff to follow the schedule. You should try to not miss any visits.

We will draw about 1 to 9 tubes of blood from you, depending on the visit. You might need to have extra clinic visits and laboratory tests if you have health changes that need to be checked.

Collection of Oral (mouth), Rectal and Genital Fluid Secretions for Research

If you are enrolled in Groups 2-6 and if you are willing, we will collect samples of mouth, rectal and genital fluid secretions for research at some clinic visits. If you are enrolled in Groups 7 and 8 and if you are willing, we will collect samples of mouth fluid secretions for research at some clinic visits. Collection of these samples is encouraged but not required. You may choose not to donate these samples but still take part in the study.

These samples are collected to see how the study product spreads throughout your body. These sample collections will not be for checking your health and do not replace routine health care.

- Oral samples may be collected 3-6 times depending on the study group.
- Rectal fluid samples may be collected at 3 visits depending on the study group.
- Vaginal fluid secretions samples may be collected at the same visits that rectal fluid samples are collected. Before a vaginal fluid sample is collected, a pregnancy test will be done for women who are able to have children. If menses occur on a scheduled day of vaginal sample collections, the collection will not be done.

Oral, rectal and vaginal fluid secretions will be collected with small disposable sponges made for

this purpose. The sponges are safe for use in sensitive areas of the body, and each sponge is new and sterile. The design is similar to a "Q-tip" that can be placed carefully so the sponge end absorbs liquid.

HIV TESTING

As part of your participation in this study, we will test you for HIV infection and possibly for other viral infections if needed to check your eligibility for the study. If you are infected with HIV, you will not be able to participate in this study. We will tell you what the results mean, how to find care, how to avoid infecting others, how we report HIV infection, and the importance of informing your partners that may be at risk because of your HIV infection.

If you have questions regarding the HIV testing, you are encouraged to discuss them with the study nurse or doctor, or you may call a NIH Clinical Center HIV counselor at 301-496-2381.

MONITORING OF THE STUDY

A group of physicians and scientists at NIH will monitor this study. This group will review the information from the study and will pay close attention to possible harmful reactions. If serious side effects occur, product administrations may be delayed or canceled.

GENETIC TESTING

Some of the blood drawn from you as part of this study will be used for genetic tests. Some genetic tests are done in research studies to see if genetic differences in people cause different types of immune responses. Your blood sample used in these genetic tests will not have your name on it and the results will not be in your medical record. These tests are not used to check your health and we will not tell you the results.

A special genetic test, called HLA typing, may be ordered through the NIH Clinical Center medical laboratory. If this test is done at the NIH Clinical Center, your HLA type results will be in your medical record. These results are not used to check your health. Any genetic testing, including HLA testing, is for research purposes only. Any genetic information collected or learned about you will be kept confidential. Medical records, including HLA test results are kept securely. We will not give any genetic information that is in your medical record to anyone without your permission.

STORED SAMPLES

We will collect samples (including blood, and possibly oral, rectal and vaginal secretions) from you during the study. We will keep these samples for future research to learn more about monoclonal antibodies, vaccines, the immune system, and/or other medical conditions. Results from research done with your stored samples will not be in your medical record or reported to you.

Labeling of Stored Samples: We will label your stored samples by a code (like a number). Only the study team can link this code to you. Any identifying information about you will be kept confidential as much as the law allows. Despite protections, there is a small chance that information identifying you will be given to someone who should not get it.

Risks from Stored Samples: There is a risk of unplanned release of information from your medical records. The chance that this information will be given to an unauthorized person without your permission is very small. Possible problems with the unplanned release of

information include discrimination when applying for insurance and employment. Similar problems may occur if you give information about yourself or agree to have your medical records released.

Future studies: In the future, other investigators (at NIH or outside of NIH) may wish to study your stored samples. When your stored samples are shared, they will be marked with a code. Your samples will not have any identifying information on them. Some information about you, such as your gender, age, health history, or ethnicity may also be shared with other researchers.

Any future research studies using your samples will be conducted in a way that protects the rights and privacy of study participants.

Your stored samples will be used only for research and will not be sold. The research done with your materials may be used to develop new products in the future but you will not receive payment for such products.

Making your Choice: You cannot take part in this study if you do not want us to collect or store your blood samples. If you agree to take part in this study, you must also agree to let us keep any of your samples for future research. If you decide not to take part in this study, you may still take part in other studies at NIH.

POSSIBLE STUDY RISKS

Risks from IV infusions or SC injections:

It is possible that you may have some side effects. General risks of methods that use a needle include stinging, discomfort, pain, soreness, redness, bruising, swelling or a tiny cut at the needle insertion site.

Risks of VRC01 and VRC01LS: This study is the first time that VRC01LS is being given to healthy people.

VRC01LS is almost identical to VRC01. About 350 adults and 21 HIV-uninfected infants have received one or more doses of VRC01 by IV or SC routes as of July 27, 2016. There were no safety concerns and no concerning reactions to the product. However, VRC01LS may have additional unknown risks and side effects.

There are several antibody products that are permitted for use in people. Other antibody products have been given safely by both the IV and the SC route. Local reactions at the site of the SC injections are common, but these reactions are usually mild and resolve in a few days. Most side effects tend to occur within the first 24 hours.

Side effects to study product infusions may include fever, chills, shaking, nausea, vomiting, pain, headache, dizziness, trouble breathing, high or low blood pressure, itchiness, rash, hives, lip or face swelling, diarrhea, racing heart or chest pain. These reactions may be related to how fast the antibody product is given. However, we rarely saw these reactions when VRC01 was given. When reactions were reported, they were usually mild. VRC01 given by the SC route has sometimes caused mild itchiness, redness and/or swelling at site of injection. These symptoms usually cleared within a few minutes to hours after the product was given. In an ongoing study, additional product injections were discontinued for one subject who developed mild chest discomfort and for one subject who developed mild rash that were associated with study product administration (VRC01 or placebo).

We are giving VRC01 and VRC01LS at a controlled rate. If symptoms occur while the product is being given, tell the nurse. Slowing or stopping the flow rate may help improve the symptoms.

Some antibody products have a risk of serious allergic reactions that can be life-threatening.

- Anaphylaxis is one type of allergic reaction that may happen soon after an antibody product is given. This reaction can include difficulty breathing, low blood pressure, hives or rash, swelling in the mouth and face.
- Serum sickness is a delayed type of allergic reaction that may happen several days to three weeks after an antibody product is given. This reaction can include hives or rash, fever, enlarged lymph nodes, muscle pains, joint pains, chest discomfort and shortness of breath.

Some antibodies of the type that attack human proteins can increase the risk of serious infections. VRC01LS is not expected to increase the risk of serious infections because it attacks a virus and not a human protein.

In addition to the possible risks that are listed above, VRC01 and VRC01LS may have other side effects that we do not know about yet. Participation in this study may affect your eligibility for future MAb studies.

We will give you any new information about risks or other information that becomes available that may affect your decision to continue in the study.

Risks of Blood Drawing: Blood drawing may cause pain and bruising and rarely, may cause a feeling of lightheadedness or fainting. Rarely, it may cause infection at the site where the blood is taken. In this study on the day of an infusion, an IV line may be placed in your vein and left for a few hours. Problems at the IV site are usually mild and may include pain, bruising, minor swelling or bleeding. Rarely, there may be an infection, vein irritation, or a blood clot.

Risks of Mucosal Sample Collection: Collection of samples by swabs and wicks by rubbing them over the mucosal surfaces can cause brief discomfort and, rarely, a little bleeding.

Risk of a False Positive HIV Antibody Test Caused by VRC01 and VRC01LS: An HIV antibody test is the usual way to test for HIV infection. VRC01 and VRC01LS are antibodies against HIV. Based on laboratory testing, VRC01 does not cause a positive HIV antibody test in standard diagnostic tests. Since VRC01LS is very similar to VRC01, we also expect that VRC01LS does not cause a positive HIV antibody test. However, if you need an HIV test for any reason while you are on the study, we prefer that you ask the VRC Clinic to do the test. We will let you know your HIV test results.

Risks during Pregnancy: We do not know what effects VRC01 and VRC01LS may have on a fetus or nursing infant. Women who are able to have children must agree to not get pregnant during study participation. We will discuss effective birth control methods with you.

You must notify the clinic staff right away if you have become pregnant during this study or think that you might be pregnant. If you become pregnant, you will not get any more VRC01 administrations, and we will not collect any more blood for research or mucosal samples. However, you will be asked to continue with study follow-up visits to check on your health and to report the outcome of the pregnancy to us, which will be reported to the antiretroviral pregnancy registry (http://www.apregistry.com).

POSSIBLE BENEFITS

This study will not provide you with any direct health benefit. You and others may benefit in the future from the information that we learn from the study.

COSTS TO YOU FOR YOUR PARTICIPATION

There are no costs to you for participating in this study. You or your health insurance will have to pay for all medical costs for medical care that you get outside this study. It is possible that you may have some expenses that are not covered by the study compensation provided.

COMPENSATION TO YOU FOR YOUR PARTICIPATION

You will be compensated for your time and inconvenience in accordance with the NIH Clinical Research Volunteer Program. It is possible that you may have some expenses that are not covered by the compensation provided.

The compensation is $175 for scheduled visits with blood drawing and oral swabs; $325 for IV product administration visit(s) that includes follow-up blood sample collections on the same day, and $275 for SC product administration visit(s) without follow-up sample collections on the same day. Compensation for timely completion of all 3 days of an electronic diary will be $25 total. Additional compensation when rectal and/or vaginal secretion collections are done will be $150, and clinic visits that do not include research blood sample collection will be $75.

Total compensation for completion of the study is estimated to range from $2425 to $5375. Actual compensation is based on the number and type of study visits you complete. Your compensation may need to be reported to the internal revenue service (IRS) as taxable income.

REASONS FOR REMOVING YOU FROM THE STUDY WITHOUT YOUR CONSENT

You may be stopped from getting the study product for several different reasons, including:

- You don't keep appointments or follow study procedures.
- You get a serious illness that needs ongoing medical care.
- You have a serious side effect thought to be due to the study product.
- You enroll in another research study at the same time you are in this study.
- You become pregnant.
- The study is stopped or canceled.

A study may be stopped or canceled by a study sponsor, a regulatory agency or by the study investigators. If this happens you will be told the reason why.

You may choose to stop participating in the study at any time. If you got any doses of VRC01LS or VRC01, you will be asked to keep follow-up visits so we can monitor your health. Collection of samples that are for research purposes only may be stopped.

ALTERNATIVES

This study is not designed to treat or prevent any disease. You may choose to not participate in this study. You may be eligible for other studies.

CONFLICT OF INTEREST

The NIH research staff is checked yearly for conflicts of interest. You may ask the research team for more information on this process. This study may have investigators who are not NIH

employees. Non-NIH investigators are expected to follow the principles of the Protocol Review Guide but are not required to report their personal financial holdings to the NIH.

The NIH, including some members of the VRC scientific staff, developed the investigational product being used in this research study. The results of this study could play a role in whether the FDA will approve the study product for sale at some time in the future. If approved, the future sale of the study product could lead to payments to NIH and some NIH scientists. By U.S. law, government scientists are required to receive such payments for their inventions. You will not receive any money from the development or sale of the product.

Manufacturing process of the investigational agent used in this trial is currently not available as a stock option by any commercial entity.

CLINICALTRIALS.GOV

A description of this clinical trial will be available on *http://www.ClinicalTrials.gov*. This Web site will not include information that can identify you. At most, the website will include a summary of the results. You can search this website at any time.

OTHER PERTINENT INFORMATION

1. Confidentiality. When results of an NIH research study are reported in medical journals or at scientific meetings, the people who take part are not named and identified. In most cases, the NIH will not release any information about your research involvement without your written permission. However, if you sign a release of information form, for example, for an insurance company, the NIH will give the insurance company information from your medical record. This information might affect (either favorably or unfavorably) the willingness of the insurance company to sell you insurance.

The Federal Privacy Act protects the confidentiality of your NIH medical records. However, you should know that the Act allows release of some information from your medical record without your permission, for example, if it is required by the Food and Drug Administration (FDA), members of Congress, law enforcement officials, or other authorized people.

2. Policy Regarding Research-Related Injuries. The Clinical Center will provide short-term medical care for any injury resulting from your participation in research here. In general, no long-term medical care or financial compensation for research-related injuries will be provided by the National Institutes of Health, the Clinical Center, or the Federal Government. However, you have the right to pursue legal remedy if you believe that your injury justifies such action.

3. Payments. The amount of payment to research volunteers is guided by the National Institutes of Health policies.

4. Problems or Questions. If you have any problems or questions about this study or about any research-related injury, contact the Principal Investigator, Julie Ledgerwood, D.O., or the Study Coordinator, Jamie Saunders, RN, BSN at 301-451-8715.

If you have any questions about your rights as a research subject, you may call the Clinical Center Patient Representative at 301-496-2626.

5. Consent Document. Please keep a copy of this document in case you want to read it again.

COMPLETE APPROPRIATE ITEM(S) BELOW:

Adult Study Participant's Consent

I have read the explanation about this study and have been given the opportunity to discuss it and to ask questions. I hereby consent to take part in this study.

Time

Signature of Adult Participant/Legal Representative Date

Print Name

THIS CONSENT DOCUMENT HAS BEEN APPROVED FOR USE FROM XXXXXX THROUGH XXXXXX.

Signature of Investigator/Person Obtaining Consent Date

Print Name

Signature of Witness Date

Print Name

APPENDIX II
CONTACT INFORMATION

**Principal Investigator**:<br>Julie Ledgerwood, D.O. 301-594-8502<br>Vaccine Research Center, NIAID, NIH<br>Bethesda, MD 20892-3017<br><br>**Associate Investigators:**<br>Joseph Casazza, M.D., Ph.D. 301-594-8627<br>Barney S. Graham, M.D., Ph.D. 301-594-8468<br>Grace Chen, M.D., MPH 240-669-2809<br>Martin Gaudinski, M.D. 301-761-7094<br>Cynthia Starr Hendel, CRNP 301-402-1341<br>LaSonji Holman, FNP 301-402-8641<br>Sarah Plummer, RN, MSN, NP 301-402-8640<br><br>**Study Coordinators/Research Nurses**<br>Jamie Saunders, RN, BSN, Study Coordinator<br>All Clinic Staff: 301-451-8715<br>Ingelise Gordon, RN<br>Pamela Costner, RN, BSN<br>Jennifer Cunningham, RN, BSN<br>Brenda Larkin, RN, BSN, CCRC<br>Carol Levinson, RN, BSN, CMSRN<br>Floreliz Mendoza, RN<br>Laura Novik, RN, MA, CCRC<br>William Whalen, RN, BSN<br>Kathryn Zephir, RN, BSN, MS<br>Xioalin Wang, RN<br><br>**DAIDS Medical Officer:**<br>Margarita Gomez Lorenzo, M.D.<br>Division of AIDS, NIAID, NIH<br>5601 Fishers Lane, 9C40<br>Bethesda, MD 20892<br><br>**Protocol Statistician**:<br>Zonghui Hu, Ph.D., 301-451-2434<br>Biostatistics Research Branch<br>Division of Clinical Research, NIAID, NIH<br><br>**Pharmacokinetics Consultant:**<br>Edmund Capparelli, Pharm.D. 858-246-0009<br>University of California, San Diego<br><br>**Data Management**:<br>Vaccine Research Center, NIAID, NIH and<br>EMMES Corporation, Rockville, MD<br><br>**Study Site**:<br>National Institutes of Health Clinical Center<br>Bethesda, MD 20892<br><br>**Product Manufacturer**: VRC Production Plant, operated by Vaccine Clinical Materials Program, Leidos Biomedical Research, Inc., Frederick, MD	**Scientific and Laboratory Collaborators**:<br>Vaccine Research Center, NIAID, NIH:<br>John Mascola, M.D., 301-594-8490<br>Richard Koup, M.D., 301-594-8585<br>Daniel Douek, M.D., 301-594-8484<br>Robert Bailer, Ph.D., 301-594-8481<br>Emily Coates, Ph.D., 301-402-4581<br><br>Medical University of South Carolina:<br>Janardan P. Pandey, PhD, 843-792-4360<br><br>**HLA Immunogenetics Laboratory**<br>Mary Carrington, Ph.D.; 301-846-1390<br>Laboratory of Experimental Immunology<br>National Cancer Institute, Frederick, MD 21702<br><br>**Research Immunology Central Laboratory:**<br>NVITAL (NIAID Vaccine Immune T-Cell and Antibody Laboratory)<br>9 West Watkins Mill Road, Suite 150<br>Gaithersburg, MD 20878<br><br>**Pharmacy:**<br>Hope DeCederfelt, R.Ph. 301-496-1031<br>Pharmaceutical Development Section<br>Clinical Center, Building 10/1N257<br>Bethesda, MD 20892<br><br>**VRC Production and Regulatory Affairs:**<br>*Michelle Conan-Cibotti, Ph.D., 240-292-4938<br>(*sponsor authorized representative for this IND)<br>KC Cheng, Ph.D., 301-761-7326<br>Florence Kaltovich, M.S., 301-761-6914<br>Judy Stein, MPH, MBA, 734-763-7753<br><br>**VRC Protocol Operations:**<br>Galina Yamshchikov, M.S., 301-761-7056<br>Nina Berkowitz, MPH, 240-747-7940<br>Ro Shauna Rothwell, PhD, 301-761-7465<br>Maria Burgos Florez, M.Sc., 301-761-7338<br>Olga Vasilenko, M.S., 301-402-8646<br>Iris Pittman, BA, CCRP, 301-761-6994<br><br>**DAIDS, Regulatory Support Center:**<br>SAE Phone: 1-800-537-9979 or 301-897-1709<br>SAE Fax: 1-800-275-7619 or 301-897-1710<br>SAE e-mail: DAIDSRSCSafetyOffice@tech-res.com<br>Protocol Registration<br>Fax: 1-800-418-3544 or 301-897-1701<br>Phone: 301-897-1707<br>e-mail: protocol@tech-res.com<br><br>**Site And Data Monitoring:**<br>PPD, Wilmington, NC

APPENDIX III
SCHEDULE OF EVALUATIONS

Schedule 1: VRC01LS; Groups 1 (5mg/kg IV), 3 (20mg/kg IV), and 4 (40mg/kg IV)

Visit Number			01R	02	02A	02B	02C	02D	03	04	06	07	08	09	10	11	15	16	17
Time After Day 0 Infusion				Pre	EOI	1hr	3h	6h	24hr	48hr	Wk1	Wk2	Wk3	Wk4	Wk8	Wk12	Wk16	Wk20	Wk24
[1]Day of Study			-42 to -1	D0	D0	D0	D0	D0	D1	D2	D7	D14	D21	D28	D56	D84	D112	D140	D168
**Clinical**	Tube	Screen	Enroll	Day of infusion															
VRC 500 Screening Consent		X																	
VRC 606 AoU; Consent			X																
[2] Screen: Physical exam, ht, wt; Other: targeted exam, BP, pulse, temp; also wt at visit 02		X	X	X	X				X	X	X	X	X	X	X	X	X	X	X
Complete med history at screen; then interim med history		X	X	X					X	X	X	X	X	X	X	X	X	X	X
[3]VRC01LS Administration				X															
Begin 3-day Solicited Systemic AEs				X															
CBC / differential	EDTA	3		3						3	3	3		3					
ALT, AST, ALP, creatinine	GLT	4		4						4	4	4		4					
Urine protein		X		X								X							
[4]Pregnancy test: urine or serum		X	X	X							[X]	X				[X]	X		X
[4]Pregnancy prevention counseling/ Reproductive Information Form		X	X	X							[X]	X				[X]	X		X
HIV EIA (other tests, if needed)	SST	4									4								
[6]HLA type	EDTA															20			
**Research Samples**																			
Timed PK samples	SST			4	4	4	4	4	4	4	4	4	4	4	4	4	4	4	4
[5]Oral sample (all participants)			[X]								[X]					[X]			
[5]Rectal sample (all participants); Cervical (females)			[X]								[X]					[X]			
PBMC and plasma	EDTA	20	20											20					
Serum	SST	24	24	16						16	16	16	16	16	16	16	16	16	16
**Daily Volume (mL)**		**55**	**44**	**27**	**4**	**4**	**4**	**4**	**4**	**27**	**31**	**27**	**20**	**47**	**20**	**40**	**20**	**20**	**20**
**Cumulative Volume (mL)**		**55**	**99**	**126**	**130**	**134**	**138**	**142**	**146**	**173**	**204**	**231**	**251**	**298**	**318**	**358**	**378**	**398**	**418**

[1] Day 0=day of first product administration. Day 0 is preferably scheduled within 14 days after enrollment, but may be scheduled up to 42 days after enrollment to allow for the possibility of study pauses or scheduling difficulty. Day 0 evaluations prior to VRC01LS administration are the baseline for assessing subsequent AEs.

[2] Screening includes physical exam with vital signs, height (ht) and weight (wt). At other visits, if medically indicated, a targeted exam is performed. Otherwise only blood pressure (BP), pulse, and temperature are required, except at Visit 02 when the current weight is also obtained to use for ordering the study agent dosed on a "mg/kg" basis.

[3]The PK blood draw "visits," defined by hours after an infusion, are relative to the exact time of the end of infusion (EOI). The exact start and end times of product administration, and the time of each PK draw are recorded to ensure accurate PK analysis.

[4] Negative pregnancy test results must be confirmed for women of reproductive potential prior to each study agent administration and prior to cervical mucosal sample collections. Complete the Reproductive Information Form when pregnancy test is performed.

[5]Mucosal sample collection is encouraged but not mandatory. Carefully schedule the mucosal sample collection visits for women so that they will occur between menstrual periods; cervical sample collection may be skipped at other mucosal timepoints if blood contamination is likely. Mucosal samples will not be collected for Group 1 participants.

[6]HLA type blood sample is collected once at any timepoint in the study and is shown as a Visit 11 evaluation for convenience; however, if HLA type is already available in the medical record it does not need to be repeated. HLA type may also be obtained from a frozen sample.

Visit windows: Visits 02A, 02B and 02C (±10 min); Visit 02D (-2 hrs), Visits 03, 04 (± 6 hrs); Visits 06, 07, 08, 09 (±2 days), and Visits 10, 11, 15, 16 and 17 (±7 days). Visits 05, 12, 13 and 14 are not applicable to Schedule 1.

Schedule 2: VRC01LS; Group 2 (5mg/kg SC)

Visit Number			01R	02	02A	03	04	05	06	07	08	09	10	11	15	16	17
Time After Day 0 Infusion				Pre	EOI	24hr	48hr	72hr	Wk1	Wk2	Wk3	Wk4	Wk8	Wk12	Wk16	Wk20	Wk24
[1]Day of Study			-42 to -1	D0	D0	D1	D2	D3	D7	D14	D21	D28	D56	D84	D112	D140	D168
**Clinical**	Tube	Screen	Enroll	Day of injection													
VRC 500 Screening Consent		X															
VRC 606 AoU; Consent			X														
[2] Screen: Physical exam, ht, wt; Other: targeted exam, BP, pulse, temp; also wt at visit 02		X	X	X	X	X	X	X	X	X	X	X	X	X	X	X	X
Complete med history at screen; then interim med history		X	X	X		X	X	X	X	X	X	X	X	X	X	X	X
[3]VRC01LS Administration				X													
Begin 3-day Solicited Systemic AEs				X													
CBC / differential	EDTA	3		3			3		3	3		3					
ALT, AST, ALP, creatinine	GLT	4		4			4		4	4		4					
Urine protein		X		X						X							
[4]Pregnancy test: urine or serum		X	X	X					[X]	X				[X]	X		X
[4]Pregnancy prevention counseling/ Reproductive Information Form		X	X	X					[X]	X				[X]	X		X
HIV EIA (other tests, if needed)	SST	4						4									
[6]HLA type	EDTA													20			
**Research Samples**																	
Timed PK samples	SST			4		4	4	4	4	4	4	4	4	4	4	4	4
[5]Oral sample (all participants)			[X]						[X]					[X]			
[5]Rectal sample (all participants); Cervical (females)			[X]						[X]					[X]			
PBMC and plasma	EDTA	20	20									20					
Serum	SST	24	24	16			16	16	16	16	16	16	16	16	16	16	16
**Daily Volume (mL)**		**55**	**44**	**27**	**0**	**4**	**27**	**24**	**27**	**27**	**20**	**47**	**20**	**40**	**20**	**20**	**20**
**Cumulative Volume (mL)**		**55**	**99**	**126**	**126**	**130**	**157**	**181**	**208**	**235**	**255**	**302**	**322**	**362**	**382**	**402**	**422**

[1] Day 0=day of first product administration. Day 0 is preferably scheduled within 14 days after enrollment, but may be scheduled up to 42 days after enrollment to allow for the possibility of study pauses or scheduling difficulty. Day 0 evaluations prior to VRC01LS administration are the baseline for assessing subsequent AEs.

[2] Screening includes physical exam with vital signs, height (ht) and weight (wt). At other visits, if medically indicated, a targeted exam is performed. Otherwise only blood pressure (BP), pulse, and temperature are required, except at Visit 02 when the current weight is also obtained to use for ordering the study agent dosed on a "mg/kg" basis.

[3]The PK blood draw "visits," defined by hours after an infusion, are relative to the exact time of the end of infusion (EOI). The exact start and end times of product administration, and the time of each PK draw are recorded to ensure accurate PK analysis.

[4] Negative pregnancy test results must be confirmed for women of reproductive potential prior to each study agent administration and prior to cervical mucosal sample collections. Complete the Reproductive Information Form when pregnancy test is performed.

[5]Mucosal sample collection is encouraged but not mandatory. Carefully schedule the mucosal sample collection visits for women so that they will occur between menstrual periods; cervical sample collection may be skipped at other mucosal timepoints if blood contamination is likely.

[6]HLA type blood sample is collected once at any timepoint in the study and is shown as a Visit 11 evaluation for convenience; however, if HLA type is already available in the medical record it does not need to be repeated. HLA type may also be obtained from a frozen sample.

<u>Visit windows</u>: Visit 02A (±10 min); Visits 03, 04, 05 (± 6 hrs); Visits 06, 07, 08, 09 (±2 days); Visits 10, 11, 15, 16, 17 (±7 days). Visits 12-14 are not applicable to Schedule 2.

Schedule 3: VRC01LS; Group 5 (5 mg/kg SC by repeat dosing)																							
Visit Number			01R	02	02A	03	04	05	06	07	09	10	11	11A	11E	12	13	14	15	16	17	17A	17E
Time After Day 0 Infusion				Pre D0	EOI D0	24hr	48h	72hr	1wk	Wk2	Wk4	Wk8	Pre Wk12	EOI Wk12	Wk12	72hr	Wk13	Wk14	Wk16	Wk20	Pre Wk24	EOI Wk24	Wk24
[1]Day of Study			-42 to -1	D0	D0	D1	D2	D3	D7	D14	D28	D56	D84	D84	D85	D87	D91	D98	D112	D140	D168	D168	D169
**Clinical**	Tube	Screen	Enroll	Day of injection									Day of injection								Day of injection		
VRC 500 Screening Consent		X																					
VRC 606 AoU; Consent			X																				
[2] Screen: Physical exam, ht, wt; Other: targeted exam, BP, pulse, temp; also wt at visits 02, 11, and 17		X	X	X	X	X	X	X	X	X	X	X	X	X		X	X	X	X	X	X	X	
Complete med history at screen; then interim med hx		X	X	X		X	X	X	X	X	X	X	X			X	X	X	X	X	X		
[3]VRC01LS Administration				X									X								X		
Begin 3-day Solicited Systemic AEs				X									X								X		
Phone contact; clinic visit if indicated															X								X
CBC / diff	EDTA	3		3			3		3	3		3	3				3	3		3	3		
ALT, AST, ALP, creatinine	GLT	4		4			4		4	4		4	4				4	4		4	4		
Urine protein		X		X						X			X					X			X		
[4]Pregnancy test: urine or serum		X	X	X						X			X					X			X		
[4]Pregnancy prevention counseling/ Reproductive Information Form		X	X	X						X			X					X			X		
HIV EIA (other tests, if needed)	SST	4						4								4							
**Research Samples**																							
Timed PK samples	SST			4		4	4	4	4	4	4	4	4			4	4	4	4	4	4		
[5]Oral sample (all participants)			[X]										[X]								[X]		
[5]Rectal sample (all participants); Cervical (females)			[X]																				
PBMC and plasma	EDTA	20	20																				
Serum	SST	24	24	16			16	16	16	16	16	16	16				16	16	16	16	16		
**Daily Volume (mL)**		**55**	**44**	**27**	**0**	**4**	**27**	**24**	**27**	**27**	**20**	**27**	**27**	**0**	**0**	**8**	**27**	**27**	**20**	**27**	**27**	**0**	**0**
**Cumulative Volume (mL)**		**55**	**99**	**126**	**126**	**130**	**157**	**181**	**208**	**235**	**255**	**282**	**309**	**309**	**309**	**317**	**344**	**371**	**391**	**418**	**445**	**445**	**445**

[1] Day 0=day of first product administration. Day 0 is preferably scheduled within 14 days after enrollment, but may be scheduled up to 42 days after enrollment to allow for the possibility of study pauses or scheduling difficulty. Day 0 evaluations prior to VRC01LS administration are the baseline for assessing subsequent AEs.
[2] Screening includes physical exam with vital signs, height (ht) and weight (wt). At other visits, if medically indicated, a targeted exam is performed. Otherwise only blood pressure (BP) and temperature are required, except at Visits 02, 11, and 16 when the current weight is also obtained to use for ordering the study agent dosed on a "mg/kg" basis.
[3]The PK blood draw "visits," defined by hours after an injection, are relative to the exact time of the end of injection (EOI). The exact start and end times of product administration and the time of each PK draw are recorded to ensure accurate PK analysis.
[4] Negative pregnancy test results must be confirmed for women of reproductive potential prior to each study agent administration and prior to cervical mucosal sample collections. Complete reproductive information form when pregnancy test is performed.
[5] Mucosal sample collection is encouraged but not mandatory. Carefully schedule the mucosal sample collection visits for women so that they will occur between menstrual periods; cervical sample collection may be skipped at other mucosal timepoints if blood contamination is likely.
* Subjects who discontinue VRC01LS may be followed weekly for 4 weeks and then monthly up to 24 weeks after the last injection as per Schedule 3 (continuation).

Visit windows: Visit A (±10 min); Visit E (+ 1 day); Visits 03, 04, 05, 12, and 18 (± 6 hrs); Visits 06, 07, 13, 14 (±2 days); Visits 09, 10, 11, 15, 16 and 17 (±7 days). Visits 08, 11B, 11C, 17B, and 17C are not applicable to Schedule 3.

Schedule 3 (continuation): VRC01LS; Group 5 (5 mg/kg SC by repeat dosing)											
Visit Number		18	19	20	21	22	23	24	25	26	27
Time After Day 0 Infusion		72 hr	Wk25	Wk26	Wk27	Wk28	Wk32	Wk36	Wk40	Wk44	Wk48
Day of Study		D171	D175	D182	D189	D196	D224	D252	D280	D308	D336
**Clinical**	Tube										
VRC 500 Screening Consent											
VRC 606 AoU; Consent											
[2] Screen: Physical exam, ht, wt; Other: targeted exam, BP, pulse, temp		X	X	X	X	X	X	X	X	X	X
Complete med history at screen; then interim med hx		X	X	X	X	X	X	X	X	X	X
CBC / diff	EDTA		3	3		3					
ALT, AST, ALP, creatinine	GLT		4	4		4					
Urine protein				X							
[4]Pregnancy test: urine or serum				X		[X]		X	[X]		X
[4]Pregnancy prevention counseling / Reproductive Information Form				X		[X]		X	[X]		X
HIV EIA (other tests, if needed)	SST	4									
[6]HLA type	EDTA			20							
**Research Samples**											
Timed PK samples	SST	4	4	4	4	4	4	4	4	4	4
[5]Oral sample (all participants)						[X]			[X]		[X]
[5]Rectal sample (all participants); Cervical (females)						[X]			[X]		
PBMC and plasma	EDTA			20							
Serum	SST		16	16	16	16	16	16	16	16	16
**Daily Volume (mL)**		**8**	**27**	**67**	**20**	**27**	**20**	**20**	**20**	**20**	**20**
**Cumulative Volume (mL)**		**453**	**480**	**547**	**567**	**594**	**614**	**634**	**654**	**674**	**694**

[2] Screening includes physical exam with vital signs, height (ht) and weight (wt). At other visits, if medically indicated, a targeted exam is performed. Otherwise only blood pressure (BP) and temperature are required, except at Visits 02, 11, and 16 when the current weight is also obtained to use for ordering the study agent dosed on a "mg/kg" basis.
[4] Negative pregnancy test results must be confirmed for women of reproductive potential prior to each study agent administration and prior to cervical mucosal sample collections. Complete the Reproductive Information Form when pregnancy test is performed.
[5] Mucosal sample collection is encouraged but not mandatory. Carefully schedule the mucosal sample collection visits for women so that they will occur between menstrual periods; cervical sample collection may be skipped at other mucosal timepoints if blood contamination is likely.
[6]HLA type blood sample is collected once at any timepoint in the study and is shown as a Visit 18 evaluation for convenience; however, if HLA type is already available in the medical record it does not need to be repeated. HLA type may also be obtained from a frozen sample.
* Subjects who discontinue VRC01LS may be followed weekly for 4 weeks and then monthly up to 24 weeks after the last injection as per Schedule 3 (continuation).
Visit windows: Visits 19-22 (±2 days), and Visits 23-27 (±7 days).

Schedule 4: VRC01LS; Group 6 (20 mg/kg IV by repeat dosing)

Visit Number			01R	02	02A	02B	02C	02D	03	04	06	07	09	10	11	11A	11B	11E	13	14	15	16	17	17A	17B
Time After Day 0 Infusion				Pre D0	EOI D0	1hr	3h	6h	24hr	48h	Wk1	Wk2	Wk4	Wk8	Pre Wk12	EOI Wk12	1hr	Wk12	Wk13	Wk14	Wk16	Wk20	Pre Wk24	EOI Wk24	1hr
[1]Day of Study			-42 to -1	D0	D0	D0	D0	D0	D1	D2	D7	D14	D28	D56	D84	D84	D84	D85	D91	D98	D112	D140	D168	D168	D168
**Clinical**	Tube	Screen	Enroll	Day of infusion											Day of infusion								Day of Infusion		
VRC 500 Screening Consent		X																							
VRC 606 AoU; Consent			X																						
[2] Screen: Physical exam, ht, wt; Other: targeted exam, BP, pulse, temp; also wt at visits 02, 11, 17		X	X	X	X				X	X	X	X	X	X	X	X			X	X	X	X	X	X	
Complete med history at screen; then interim med hx		X	X	X					X	X	X	X	X	X	X				X	X	X	X	X		
[3]VRC01LS Administration				X											X								X		
Begin 3-day Solicit Systemic AE				X											X								X		
Phone contact; clinic visit if indicated																		X							
CBC / diff	EDTA	3		3						3	3	3		3	3				3	3		3	3		
ALT, AST, ALP, creatinine	GLT	4		4						4	4	4		4	4				4	4		4	4		
Urine protein		X		X								X			X					X			X		
[4]Pregnancy test: urine or serum		X	X	X								X			X					X			X		
[4]Pregnancy prevention counseling/ Reproductive Information Form		X	X	X								X			X					X			X		
HIV EIA (other tests, if needed)	SST	4									4								4						
**Research Samples**																									
Timed PK samples	SST			4	4	4	4	4	4	4	4	4	4	4	4	4	4		4	4	4	4	4	4	4
[5]Oral sample (all participants)			[X]												[X]								[X]		
[5]Rectal sample (all participants); Cervical (females)			[X]																						
PBMC and plasma	EDTA	20	20																						
Serum	SST	24	24	16						16	16	16	16	16	16				16	16	16	16	16		
**Daily Volume (mL)**		**55**	**44**	**27**	**4**	**4**	**4**	**4**	**4**	**27**	**31**	**27**	**20**	**27**	**27**	**4**	**4**	**0**	**31**	**27**	**20**	**27**	**27**	**4**	**4**
**Cumulative Volume (mL)**		**55**	**99**	**126**	**130**	**134**	**138**	**142**	**146**	**173**	**204**	**231**	**251**	**278**	**305**	**309**	**313**	**313**	**344**	**371**	**391**	**418**	**445**	**449**	**453**

[1] Day 0=day of first product administration. Day 0 is preferably scheduled within 14 days after enrollment, but may be scheduled up to 42 days after enrollment to allow for the possibility of study pauses or scheduling difficulty. Day 0 evaluations prior to VRC01LS administration are the baseline for assessing subsequent AEs.

[2] Screening includes physical exam with vital signs, height (ht) and weight (wt). At other visits, if medically indicated, a targeted exam is performed. Otherwise only blood pressure (BP), pulse, and temperature are required, except at Visits 02, 11, and 16 when the current weight is also obtained to use for ordering the study agent dosed on a "mg/kg" basis.

[3]The PK blood draw "visits," defined by hours after an injection, are relative to the exact time of the end of injection (EOI). The exact start and end times of product administration , and the time of each PK draw are recorded to ensure accurate PK analysis.

[4] Negative pregnancy test results must be confirmed for women of reproductive potential prior to each study agent administration and prior to cervical mucosal sample collections.

[5] Mucosal sample collection is encouraged but not mandatory. Carefully schedule the mucosal sample collection visits for women so that they will occur between menstrual periods; cervical sample collection may be skipped at other mucosal timepoints if blood contamination is likely.

* Subjects who discontinue VRC01LS may be followed weekly for 4 weeks and then monthly up to 24 weeks after the last infusion as per Schedule 4 (continuation).

Visit windows: Visits A, B, and C (±10 min); Visit 02D (-2 hrs); Visit E (+ 1 day); Visits 03, and 04 (± 6 hrs); Visits 06, 07, 13 and 14 (±2 days); Visits 09, 10, 11, 15, 16 and 17 (±7 days). Visits 05, 08, 12 and 18 are not applicable to Schedule 4.

Schedule 4 (continuation): VRC01LS; Group 6 (20 mg/kg IV by repeat dosing)											
Visit Number		17E	19	20	21	22	23	24	25	26	27
Time After Day 0 Infusion		Wk24	Wk25	Wk26	Wk27	Wk28	Wk32	Wk36	Wk40	Wk44	Wk48
Day of Study		D169	D175	D182	D189	D196	D224	D252	D280	D308	D336
**Clinical**	Tube										
VRC 500 Screening Consent											
VRC 602 AoU; Consent											
[2] Screen: Physical exam, ht, wt; Other: targeted exam, BP, pulse, temp			X	X	X	X	X	X	X	X	X
Complete med history at screen; then interim med hx			X	X	X	X	X	X	X	X	X
Phone contact; clinic visit if indicated		X									
CBC / diff	EDTA		3	3		3	3				
ALT, AST, ALP, creatinine	GLT		4	4		4	4				
Urine protein				X							
[4]Pregnancy test: urine or serum				X		[X]		X	[X]		X
[4]Pregnancy prevention counseling / Reproductive Information Form				X		[X]		X	[X]		X
HIV EIA (other tests, if needed)	SST		4								
[6]HLA type	EDTA			20							
**Research Samples**											
Timed PK samples	SST		4	4	4	4	4	4	4	4	4
[5]Oral sample (all participants)						[X]			[X]		[X]
[5]Rectal sample (all participants); Cervical (females)						[X]			[X]		
PBMC and plasma	EDTA			20							
Serum	SST		16	16	16	16	16	16	16	16	16
**Daily Volume (mL)**		**0**	**31**	**67**	**20**	**27**	**27**	**20**	**20**	**20**	**20**
**Cumulative Volume (mL)**		**453**	**484**	**551**	**571**	**598**	**625**	**645**	**665**	**685**	**705**

[2] Screening includes physical exam with vital signs, height (ht) and weight (wt). At other visits, if medically indicated, a targeted exam is performed. Otherwise only blood pressure (BP) and temperature are required, except at Visits 02, 11, and 16 when the current weight is also obtained to use for ordering the study agent dosed on a "mg/kg" basis.
[4] Negative pregnancy test results must be confirmed for women of reproductive potential prior to each study agent administration and prior to cervical mucosal sample collections. Complete the Reproductive Information Form when pregnancy test is performed.
[5] Mucosal sample collection is encouraged but not mandatory. Carefully schedule the mucosal sample collection visits for women so that they will occur between menstrual periods; cervical sample collection may be skipped at other mucosal timepoints if blood contamination is likely.
[6]HLA type blood sample is collected once at any timepoint in the study and is shown as a Visit 18 evaluation for convenience; however, if HLA type is already available in the medical record, it does not need to be repeated. HLA type may also be obtained from a frozen sample.
* Subjects who discontinue VRC01LS may be followed weekly for 4 weeks and then monthly up to 24 weeks after the last infusion as per Schedule 4 (continuation).
Visit windows: Visit 17E (+ 1 day); Visits 19-22 (±2 days), and Visits 23-27 (±7 days).

Schedule 5: VRC01; Group 7 (5 mg/kg SC by repeat dosing)

Visit Number			01R	02	02A	03	04	05	06	07	08	09	09A	09E	09F	09G	09H	09I	10	11	15	16	17	22
Time After Day 0 Infusion				Pre D0	EOI D0	24hr	48h	72hr	Wk1	Wk2	Wk3	Pre Wk4	EOI Wk4	24h	72hr	Wk5	Wk6	Wk7	Wk8	Wk12	Wk16	Wk20	Wk24	Wk28
[1]Day of Study			-42 to -1	D0	D0	D1	D2	D3	D7	D14	D21	D28	D28	D29	D31	D35	D42	D49	D56	D84	D112	D140	D168	D296
**Clinical**	Tube	Screen	Enroll	Day of injection								Day of injection												
VRC 500 Screening Consent		X																						
VRC 606 AoU; Consent			X																					
[2] Screen: Physical exam, ht, wt; Other: targeted exam, BP, pulse, temp; also wt at visits 02, and 09		X	X	X	X	X	X	X	X	X	X	X	X		X	X	X	X	X	X	X	X	X	X
Complete med history at screen; then interim med hx		X	X	X	X	X	X	X	X	X	X	X			X	X	X	X	X	X	X	X	X	X
[3]VRC01 Administration				X								X												
Begin 3-day Solicited Systemic AEs				X								X												
Phone contact; clinic visit if indicated														X										
CBC / diff	EDTA	3		3			3		3	3		3				3	3		3					
ALT, AST, ALP, creatinine	GLT	4		4			4		4	4		4				4	4		4					
Urine protein		X		X						X		X					X							
[4]Pregnancy test: urine or serum		X	X	X						X		X					X				X			X
[4]Pregnancy prevention counseling/ Reproductive Information Form		X	X	X						X		X					X				X			X
HIV EIA (other tests, if needed)	SST	4																						
[6]HLA type	EDTA																			20				
**Research Samples**																								
Timed PK samples	SST			4		4	4	4	4	4	4	4			4	4	4	4	4	4	4	4	4	4
[5]Oral mucosal sample			[X]									[X]							[X]			[X]		[X]
PBMC and plasma	EDTA	20	20														20							
Serum	SST	24	24	16			16	16	16	16	16	16				16	16	16	16	16	16	16	16	16
**Daily Volume (mL)**		**55**	**44**	**27**	**0**	**4**	**27**	**20**	**27**	**27**	**20**	**27**	**0**	**0**	**4**	**27**	**47**	**20**	**27**	**40**	**20**	**20**	**20**	**20**
**Cumulative Volume (mL)**		**55**	**99**	**126**	**126**	**130**	**157**	**177**	**204**	**231**	**251**	**278**	**278**	**278**	**282**	**309**	**356**	**376**	**403**	**443**	**463**	**483**	**503**	**523**

[1] Day 0=day of first product administration. Day 0 is preferably scheduled within 14 days after enrollment, but may be scheduled up to 42 days after enrollment to allow for the possibility of study pauses or scheduling difficulty. Day 0 evaluations prior to VRC01 administration are the baseline for assessing subsequent AEs.
[2] Screening includes physical exam with vital signs, height (ht) and weight (wt). At other visits, if medically indicated, a targeted exam is performed. Otherwise only blood pressure (BP) and temperature are required, except at Visits 02 and 09 when the current weight is also obtained to use for ordering the study agent dosed on a “mg/kg” basis.
[3]The PK blood draw “visits,” defined by hours after an injection, are relative to the exact time of the end of injection (EOI). The exact start and end times of product administration and the time of each PK draw are recorded to ensure accurate PK analysis.
[4] Negative pregnancy test results must be confirmed for women of reproductive potential prior to each study agent administration. Complete the reproductive information form when the pregnancy test is performed.
[5] Mucosal sample collection is encouraged but not mandatory.
[6]HLA type blood sample is collected once at any timepoint in the study and is shown as a Visit 11 evaluation for convenience; however, if HLA type is already available in the medical record, it does not need to be repeated. HLA type may also be obtained from a frozen sample.
* Subjects who discontinue VRC01 may be followed weekly for 4 weeks and then monthly up to 24 weeks after the last injection as per Schedule 3 (continuation).

Visit windows: Visits 02A and 09A (±10 min); Visit 09E (+ 1 day); Visits 03, 04, 05, and 09F (± 6 hrs); Visits 06, 07, 08, 09G, 09H, 09I, and 10 (±2 days); Visits 09, 11, 15, 16, 17, and 22 (±7 days, with not less than 21 days between injections). Visits 12, 13, 14, 18, 19, 20 and 21 are not applicable to Schedule 5.

Schedule 6: VRC01; Group 8 (20 mg/kg IV by repeat dosing)																										
Visit Number			01R	02	02A	02B	02C	02D	03	04	06	07	08	09	09A	09B	09E	09G	09H	09I	10	11	15	16	17	22
Time After Day 0 Infusion				Pre D0	EOI D0	1hr	3h	6h	24hr	48h	Wk1	Wk2	Wk3	Pre Wk4	EOI Wk4	1hr	Wk4	Wk5	Wk6	Wk7	Wk8	Wk12	Wk16	Wk20	Wk24	Wk28
[1]Day of Study			-42 to -1	D0	D0	D0	D0	D0	D1	D2	D7	D14	D21	D28	D28	D28	D29	D35	D42	D49	D56	D84	D112	D140	D168	D296
**Clinical**	Tube	Screen	Enroll	Day of infusion										Day of infusion												
VRC 500 Screening Consent		X																								
VRC 606 AoU; Consent			X																							
[2] Screen: Physical exam, ht, wt; Other: targeted exam, BP, pulse, temp; also wt at visits 02 and 09		X	X	X	X				X	X	X	X	X	X	X			X	X	X	X	X	X	X	X	X
Complete med history at screen; then interim med hx		X	X	X	X				X	X	X	X	X	X				X	X	X	X	X	X	X	X	X
[3]VRC01 Administration				X										X												
Begin 3-day Solicited Systemic AEs				X										X												
Phone contact; clinic visit if indicated																	X									
CBC / diff	EDTA	3		3						3	3	3		3				3	3		3					
ALT, AST, ALP, creatinine	GLT	4		4						4	4	4		4				4	4		4					
Urine protein		X		X								X		X					X							
[4]Pregnancy test: urine or serum		X	X	X								X		X					X				X			X
[4]Pregnancy prevention counseling/ Reproductive Information Form		X	X	X								X		X					X				X			X
HIV EIA (other tests, if needed)	SST	4																								
[6]HLA type	EDTA																		20							
**Research Samples**																										
Timed PK samples	SST			4	4	4	4	4	4	4	4	4	4	4	4	4		4	4	4	4	4	4	4	4	4
[5]Oral mucosal sample			[X]											[X]							[X]			[X]		[X]
PBMC and plasma	EDTA	20	20																			20				
Serum	SST	24	24	16						16	16	16	16	16				16	16	16	16	16	16	16	16	16
**Daily Volume (mL)**		**55**	**44**	**27**	**4**	**4**	**4**	**4**	**4**	**27**	**27**	**27**	**20**	**27**	**4**	**4**	**0**	**27**	**47**	**20**	**27**	**40**	**20**	**20**	**20**	**20**
**Cumulative Volume (mL)**		**55**	**99**	**126**	**130**	**134**	**138**	**142**	**146**	**173**	**200**	**227**	**247**	**274**	**278**	**282**	**282**	**309**	**356**	**376**	**403**	**443**	**463**	**483**	**503**	**523**

[1] Day 0=day of first product administration. Day 0 is preferably scheduled within 14 days after enrollment, but may be scheduled up to 42 days after enrollment to allow for the possibility of study pauses or scheduling difficulty. Day 0 evaluations prior to VRC01 administration are the baseline for assessing subsequent AEs.
[2] Screening includes physical exam with vital signs, height (ht) and weight (wt). At other visits, if medically indicated, a targeted exam is performed. Otherwise only blood pressure (BP) and temperature are required, except at Visits 02 and 09 when the current weight is also obtained to use for ordering the study agent dosed on a "mg/kg" basis.
[3]The PK blood draw "visits," defined by hours after an injection, are relative to the exact time of the end of injection (EOI). The exact start and end times of product administration and the time of each PK draw are recorded to ensure accurate PK analysis.
[4] Negative pregnancy test results must be confirmed for women of reproductive potential prior to each study agent administration. Complete the reproductive information form when the pregnancy test is performed.
[5] Mucosal sample collection is encouraged but not mandatory.
[6]HLA type blood sample is collected once at any timepoint in the study and is shown as a Visit 09H evaluation for convenience; however, if HLA type is already available in the medical record, it does not need to be repeated. HLA type may also be obtained from a frozen sample.
* Subjects who discontinue VRC01 may be followed weekly for 4 weeks and then monthly up to 24 weeks after the last injection as per Schedule 3 (continuation).
Visit windows: Visit 02A, 09A, 02B, 09B and 02C (±10 min); Visit 02D (-2 hrs); Visit 09E (+ 1 day); Visits 03 and 04 (± 6 hrs); Visits 06, 07, 08, 09G, 09H, 09I, and 10 (±2 days); Visits 09, 11, 15, 16, 17, and 22 (±7 days, with not less than 21 days between injections). Visits 05, 12, 13, 14, 18, 19, 20 and 21 are not applicable to Schedule 6.

APPENDIX IV
TABLE FOR GRADING SEVERITY OF ADVERSE EVENTS

The U.S. Department of Health and Human Services, National Institutes of Health, National Institute of Allergy and Infectious Diseases, Division of AIDS. Division of AIDS (DAIDS) Table for Grading the Severity of Adult and Pediatric Adverse Events, Version 2.0. [November 2014] will be used in this study. The table is available at the following link:
http://rsc.tech-res.com/docs/default-source/safety/daids_ae_grading_table_v2_nov2014.pdf

The grading table, the Manual for Expedited Reporting of Adverse Events to DAIDS, and supplementary tutorial and tools can be found on the Division of AIDS Regulatory Support Center (RSC) website:

http://rsc.tech-res.com/

The Table will be used as posted at the link above with the following exemptions:

- Weight loss will be recorded as an adverse event only if it is considered deleterious to the participant’s health.
- For severity grading of the solicited bruising parameter at the product administration site, the definitions based on size of the largest diameter and listed for the “Injection Site Erythema or Redness” will be used. The severity grade definition for “Bruising” provided under the Dermatologic Clinical Conditions will be used only for unsolicited adverse events involving bruising at other body locations.
